# Constructing xenobiotic maps of metabolism to predict enzymes catalyzing metabolites capable of binding to DNA

**DOI:** 10.1186/s12859-021-04363-6

**Published:** 2021-09-21

**Authors:** Mael Conan, Nathalie Théret, Sophie Langouet, Anne Siegel

**Affiliations:** 1 Institut de Recherche en Santé, Environnement et Travail, Univ Rennes, Inserm, EHESP, IRSET, Rennes, France; 2 Institut de Recherche en Informatique et Systèmes Aléatoires, Univ Rennes, Inria, CNRS, IRISA, Rennes, France

**Keywords:** Metabolism, Heterocyclic aromatic amines, Xenobiotics, DNA binding ability, Site of metabolism

## Abstract

**Background:**

The liver plays a major role in the metabolic activation of xenobiotics (drugs, chemicals such as pollutants, pesticides, food additives...). Among environmental contaminants of concern, heterocyclic aromatic amines (HAA) are xenobiotics classified by IARC as possible or probable carcinogens (2A or 2B). There exist little information about the effect of these HAA in humans. While HAA is a family of more than thirty identified chemicals, the metabolic activation and possible DNA adduct formation have been fully characterized in human liver for only a few of them (MeIQx, PhIP, A$\alpha$C).

**Results:**

We have developed a modeling approach in order to predict all the possible metabolites of a xenobiotic and enzymatic profiles that are linked to the production of metabolites able to bind DNA. Our prediction of metabolites approach relies on the construction of an enriched and annotated map of metabolites from an input metabolite.The pipeline assembles reaction prediction tools (SyGMa), sites of metabolism prediction tools (Way2Drug, SOMP and Fame 3), a tool to estimate the ability of a xenobotics to form DNA adducts (XenoSite Reactivity V1), and a filtering procedure based on Bayesian framework. This prediction pipeline was evaluated using caffeine and then applied to HAA. The method was applied to determine enzymes profiles associated with the maximization of metabolites derived from each HAA which are able to bind to DNA. The classification of HAA according to enzymatic profiles was consistent with their chemical structures.

**Conclusions:**

Overall, a predictive toxicological model based on an *in silico* systems biology approach opens perspectives to estimate the genotoxicity of various chemical classes of environmental contaminants. Moreover, our approach based on enzymes profile determination opens the possibility of predicting various xenobiotics metabolites susceptible to bind to DNA in both normal and physiopathological situations.

**Supplementary Information:**

The online version contains supplementary material available at 10.1186/s12859-021-04363-6.

## Background

*Heterocyclic Aromatic Amines (HAA) and their metabolites* The liver plays a major role in the metabolic activation of xenobiotics (drugs, pollutants, pesticides, food additives...). HAA are environmental contaminants formed during the cooking of meat or fish, in cigarette smoke or exhaust gas [1–3]. HAA are compounds of concern because previous studies have shown that they are mutagenic in bacteria, carcinogenic in animals and due to a lack of epidemiological studies they are classified as possible and probable carcinogens by the International Agency for Research on Cancer [4].

Over 30 HAA have been identified so far. The pyrolytic HAA, such as A$\alpha$C (*2-amino-9H*-pyrido[2,3-*b*]indole), are formed by a pyrolysis reaction of amino acids at temperature greater than 250C. Aminoimidazoarene HAA, such as MeIQx (2-amino-3,8-dimethylimidazo[4,5-*f*]quinoxaline), PhIP (2-amino-1-methyl-6-phenylimidazo[4,5-*b*]pyridine) and IQ (2-amino-3-methylimidazo[4,5-*f*]quinoline), are formed by Maillard reaction between hexose and amino acids at a temperature greater than 150C [5].

**Fig. 1:**
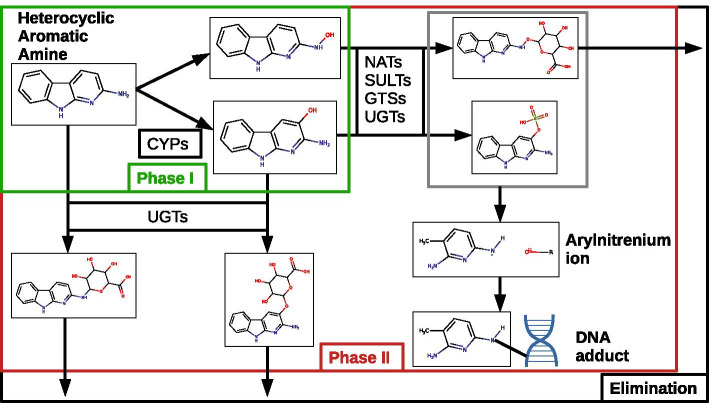
Representation of Heterocyclic Aromatic Amine Metabolism. This metabolism is divided into two steps: Phase I (in green) is known as “oxidation reaction” and catalyzed by cytochromes P450 (CYPs) and Phase II (in red) is known as a “conjugation reaction”, catalyzed by UDP-glucuronyltransferases (UGTs), N-actetyltransferases (NATs), Sulfotransferases (SULTs) or Glutathione S-transferase (GSTs)

In human beings, as illustrated in Fig. 1, HAA are first biotransformed by phase I xenobiotic metabolism enzymes which consist of an oxidation mainly catalyzed by cytochromes P450 (CYPs). The oxidated metabolite is then conjugated by phase II xenobiotic metabolic enzymes such as UDP glucuronyl transferase (UGTs), glutathione S transferase (GSTs), N-acetyltransferase (NATs) and sulfotransferase (SULTs). Conjugate metabolites can either be excreted or cleaved to form an aryl nitrenium ion, which react to DNA and therefore can form DNA adducts [2, 4].

In order to predict the genotoxicity of a metabolite, different tools infer the possibility of a compound binding to DNA (potential DNA adduct). One strategy is to search for specific chemical structures assumed to bind to DNA because a known compound, with a similar structure, has been shown to form DNA-adducts [6–8]. Another strategy to determine if a compound can form DNA adducts is based on a Quantitative Structure-Toxicity Relationship (QSAR) score that models toxicity according to molecular descriptors of compounds [6, 7, 9]. More recent tools use deep learning to infer from the descriptors of each atom if it can bind to DNA [10, 11]. A site of reactivity (SOR) score is associated to each atom of a compound and represent the probability of binding to DNA.

The main cornerstone of using toxicity prediction tools on HAA is that compounds which may bind to DNA result from one or several metabolic transformations, which are unstable and cannot be experimentally characterized. As a consequence, the bioactivation metabolism and DNA adduct formation are fully characterized for only three HAA i.e., A$\alpha$C, MeIQx and PhIP in human liver [12–15]. This advocates for the use of in silico methods to predict HAA metabolites and potential DNA adducts derived from HAA bioactivation in order to drive research about the toxicity of the HAA family.

Prediction of metabolites To overcome the lack of information about metabolic bioactivation of HAA and potential formation of DNA adducts, tools for prediction of metabolism have been developed. They allow for the identification of potential biomarkers of exposure in humans. Methods for the prediction of metabolites and reactions use biochemical transformation rules describing chemical reactions and link an input chemical structure to an output chemical structure. For a given compound, the prediction tool searches for chemical structures matching with such input structures and when they are found, the rule is applied and the resulting chemical structures are predicted as metabolites. Several tools implement such methods of prediction including MetaSite, METEOR, META, PROXIMAL, TIMES, UM-PPS BioTransformer or SyGMa [6, 16–22]. These predictions are often represented as a *metabolism map* containing the predicted metabolites and the reactions that link them. The main drawback of this type of approach is that the use of a high number of transformation rules can lead to a great number of predictions with a high number of unknown metabolites [23].

Prediction of sites of metabolism (SOM) Another method for predicting metabolite structures uses the prediction of sites of metabolism (SOM) that can reduce the high number of unknown predicted metabolites in a metabolism map. SOM-based tools predict the reaction of an atom by using a set of specific reactions. This set is generated by associating reactions catalysed by the same enzyme. It results in models that predict the probability for an atom to interact with specific enzymes or isoforms. These methods use molecular descriptors which describe different parameters of each atom of a compound. Some tools such as QMBO, CypScore, SMARTCyp or MetaSite [16, 24–28] rely on the hydrogen abstraction reaction which is the energy necessary to remove an hydrogen linked to the atom. Other tools such as Way2Drug SOMP, FAst MEtabolizer (FAME) or XenoSite Metabolism 1.0 [10, 11, 29–31] use structure parameters such as the atom nature and the nearest neighbour atoms. In these tools, machine learning methods are used to determine a score based on atom molecular descriptors, which represents the probability of an atom to be a SOM. Others SOM predictors such as IDSite, IMPACTS or MLite [32–34] use docking methods and similarities between ligand structure and structure of the compound of interest.

The literature highlights two strategies for using SOM prediction to predict metabolic maps. A first strategy classifies and evaluates the confidence of different predicted pathways by interpreting SOM as the probability of a reaction occurring in the map. Ranking pathways with these probabilities allows for the analysis of the predicted metabolites and reactions. To the state of our knowledge, this recent method was only applied to the metabolism of Terbinafine (TBF), allowing for the detection of a new pathway which can explain the formation of TBF-A from TBF [35]. Another strategy, detailed in [36] uses SOM predictions to filter metabolic maps by removing predicted reactions which are not supported by an accurate SOM prediction. In this study, the SOM-filter threshold is determined by using a training set of analog chemicals to the chemicals of interest. The method was applied to predict the metabolism of HAA using SOM predictors of CYPs and UGTs enzymes. Metabolites predictions and DNA reactivity prediction were then used to predict potential DNA adducts derived from each HAA. The potential of each HAA to form DNA adducts was finally characterized by the ratio between the number of metabolites predicted to bind to DNA and the number of total predicted metabolites. The main limitation of this approach is that the filtration of the metabolic maps relied on SOM scores associated with the reaction producing the putative DNA binding metabolites, getting rid of both the predecessor reactions which are required to produce intermediary metabolites and the possible multiple pathways that produce the same metabolite, as evidenced in [35].

Contribution To advance the ability to predict the formation of DNA adducts by HAA, we have introduced a new method which combines the concept of a filtered metabolic map introduced in [36] and the concept of ranked pathways introduced in [35]. Instead of filtering metabolic maps according to individual reaction SOM scores, we have created a *production probability score* which describes the probability of a metabolite to be produced according to one or several chains of reactions weighted by SOM scores.

Our method consists of a three steps pipeline: the first step is the prediction of metabolites of the compound of interest, the second step is the annotation of the resulted *metabolic map* using SOM scores and the third step is the computation of the production probability score for each metabolite using Bayesian networks in order to rank and filter metabolite maps.

We used caffeine to validate our modeling approach based on SOM predictions of phase I and phase II xenobiotic metabolism enzymes. Indeed, caffeine metabolism is well described and shares enzymes with HAA metabolism such as phase I enzyme especially CYP1A2, the main enzyme of caffeine metabolism, but also CYP3A4, CYP2E1, CYP2D6 and phase II enzymes including NATs. In addition, some caffeine metabolites can be produced through distinct pathways similarly to HAA predicted metabolites. After validation of the method using caffeine, the method was applied to HAA to predict enzymes involved in the formation of metabolites capable of binding to DNA.

## Results

### Definition and construction of enriched metabolic maps

**Fig. 2:**
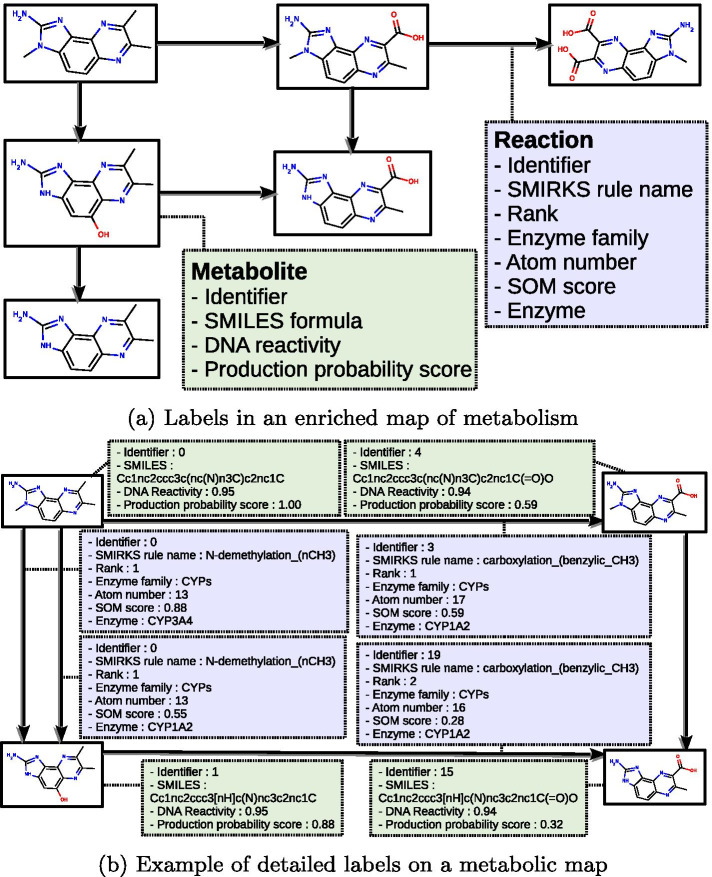
Labels of a map of metabolism. **a** Metabolites are depicted by their 2D structure in black squares. Arrows between metabolites represent reactions, which consume a metabolite in order to produce another one. Left side: labels are shown in dashed squares (green for metabolites and blue for reactions). **b** Examples of values of labels for a part of the above network. Each (a) and (b) are shown in plain page in Additional file 1

Map of metabolism We define the concept of *enriched maps of metabolism* to be oriented graphs where nodes represent chemical compounds and edges represent reactions that model the transformation of the input compound into the output compound. In these maps, different information is added to label reactions and nodes in order to enable the exploration of predicted metabolism results. As detailed below, in enriched metabolic maps, nodes are labeled by *SMILES formula*, *DNA reactivity label* and *production probability score* and edges are labeled by *rule name*, *atom number*, *rank label*, *enzyme name* and *enzyme family*. Consequently, two edges with the same enzyme family but which are different enzymes are associated with different edges. An example is shown in Fig. 2.

More precisely, nodes of enriched maps of metabolism represent chemical compounds. They are associated with a SMILES formula, which is interpreted in a 2D structure allowing for the labeling of atoms with numbers according to the International Union of Pure and Applied Chemistry (IUPAC) standard conventions [37]. Another label of the node is its *DNA reactivity label*, which is provided by site of reactivity (SOR) predictors.

Edges are first labeled by a unique identifier and by a *rule name* that refers to a SMIRKS rule which encodes the transformation [38]. Each reaction is also associated with an *atom number of reaction*, the label of the atom in 2D structure of the input compound which is transformed by the reaction to form the output compound. Finally, each edge is labeled with an *enzyme name*, which catalyzes the reaction. The *production probability score* of the edge is determined by using site of metabolism (SOM) predictors (see methods for details) and the atom number of reaction.

A specific node is identified in the graph, named the *original compound*. It is defined as the source of the map of metabolism, and the compound is described by its SMILES canonical formula available in PubChem database [39]. We then introduce a *rank* label for each edge, which describes the position of the reaction in the graph with respect to the original compound: *first rank reactions* correspond to edges having the original compound as input, *second rank reactions* corresponds to edges whose input is the output of a first rank reaction, etc...

Pipeline for building a map of metabolism Maps of metabolism were built using a combination of several tools, which are precisely described in the Methods section.

**Fig. 3:**
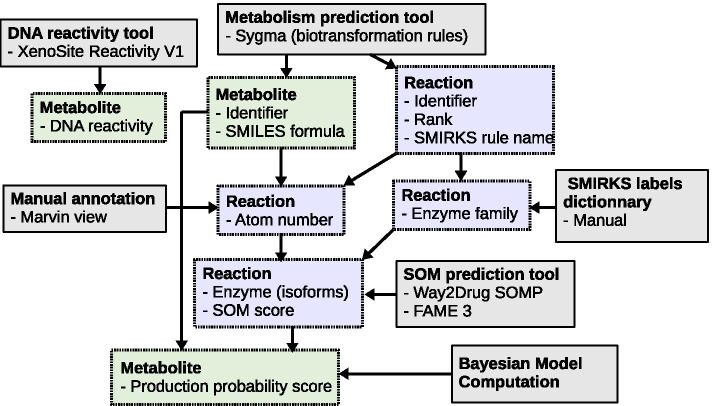
Pipeline for building an enriched map of metabolism. Scheme representation of the pipeline to obtain labels to enrich a metabolism map. Labels are depicted in green (metabolites) and blue (reactions) squares. They are computed using the various tools and methods (grey squares) which require as input the information provided by other labels. SMIRKS labels dictionary is detailed in Additional file 2

The pipeline is shown in Fig. 3 and starts with the selection of an original compound described by its SMILES canonical formula available in PubChem database. In this paper, the method was applied to 31 original compounds: caffeine (for the sake of validation of the method), and 30 HAA, see Results below.

For each original compound, the SyGMa python package [22] is applied to compute nodes (e.g, metabolites of the original compound) and edges (e.g., transformations between metabolites) of the associated map of metabolism. In our studies, SyGMa was iterated twice, in order to predict first-rank and second-rank reactions with respect to the original compound, e.g, all possible metabolites of the original compound with at most two transformations.

The *SMILES label* (and its associated 2D structure) of each node is generated by the RDKit package implemented in SyGMa. To define the *DNA reactivity label* of each node, we consider that a node is reactive with DNA if at least one of the atoms of the metabolite has a score of reactivity computed by XenoSite Reactivity [10] greater than 0.85.

For each edge, a manual curation procedure is performed to determine the *atom number of reaction*. This label represents the number of the atom, according to IUPAC numbering [37], of the input metabolite of the edge, on which the reaction occurs to produce the output metabolite of the edge. These atom numbers of reactions are obtained by manually comparing the structure of the input and output metabolites of each reaction provided by the MarvinView tool [40], in order to identify the IUPAC numbering of the transformed atom.

The next step of the pipeline consists of annotating edges with rank, rule name and enzyme labels. For each edge, the rank label is defined to be the number of iterations of SyGMa from the original compound allowing the prediction of the reaction. The rule name label is also provided by SyGMa, according to a catalogue of 176 SMIRKS rules (149 for phase I xenobiotic metabolism reactions and 27 for phase II xenobiotic metabolism reactions).

In order to label each edge with an enzyme, we created a dictionary mapping every SMIRKS rule label with an *enzyme family label* (see Additional file 2). The 149 SMIRKS rules corresponding to phase I reactions are mapped to the *CYPs* enzyme family. Among the 27 SMIRKS rule labels corresponding to phase II reactions, 25 label rules are associated with the *UGTs* (13 SMIRKS labels), *NATs* (5 SMIRKS labels), *SULTs* (6 SMIRKS labels) and *GSTs* enzyme family (1 SMIRKS labels). The two remaining SMIRKS rule labels are not related to CYPs, UGTs, NATs, GSTs or SULTs and are out of the scope of the method. The corresponding edges are removed from the metabolic map. In addition, nodes appearing to be isolated in the map after this curation are also removed from the map.

The pipeline continues with a procedure used to annotate each edge with a site of metabolism (SOM) prediction score. The procedure selects the SOM prediction tools to annotate reactions of metabolic maps following the hypothesis that reactions of first-rank can be considered mostly as phase I reactions and reactions of second-rank should contain most of the reactions of phase II. This assumption is motivated by the fact that xenobiotics metabolism is divided in two phases (Fig. 1) [2, 4, 5], a phase I, oxidative, and a phase II, conjugation. This implies that in major cases phase II reactions need first a phase I reaction to occur. In practice, our procedure depends on the *rank*, *enzyme label* and *atom number of reaction*. The main steps are (a) Reactions of first-rank can be considered mostly as phase I reactions, catalyzed by different isoforms of CYPs. Therefore, the tool Way2Drug SOMP [29] is used to compute SOM scores for edges of first-rank, because it provides refined annotations of CYP isoforms (CYP1A2, CYP3A4, CYP2D6, CYP2C9 and CYP2C19), involved in phase I metabolism. As the tool also provides annotations for reactions catalyzed by UGTs, the predicted scores for such reactions are also conserved. (b) Assuming that reactions of second-rank can be considered mostly as reactions of phase II (catalyzed by SULT, UGT, NAT, GST), the tool FAME3 [31] is used to annotate reactions of second rank, because it is associated with the largest family of phase II enzymes. Note that reactions of second-rank catalyzed by UGT are annotated with a different score than reactions of first-rank catalyzed by UGTs, in order to have homogeneous and comparable scores for reactions with the same rank. (c) Notably, when an edge can be annotated with SOMs associated with different enzymes (especially for isoform predictions), the edge is duplicated for each enzyme to avoid confusion. All reactions which could not be annotated with a SOM score are removed from the metabolic map, as well as the resulting isolated nodes.

As a final step, all the metabolites of the map are associated with a SOM-based pathway production probability score (production probability score). This score depicts the probability for each node to be formed for each metabolite, according to all the annotations of the reactions of the metabolic map. This approach relies on the formalism of Bayesian networks [41], a relevant framework to ensure that all possible production pathways contribute to the probability of metabolite production (See methods for details).

### Validation of the method: construction and analysis of a map of metabolism for caffeine

In order to validate our modeling approach based on SOM predictions of phase I and phase II xenobiotics metabolism enzymes, we applied the pipeline to caffeine. Indeed, caffeine metabolism is well described and shares enzymes with HAA metabolism, linked to xenobiotics metabolism enzymes, such as CYP1A2, the main enzyme of caffeine metabolism, but also CYP3A4, CYP2E1 and CYP2D6 [42]. Caffeine metabolism is also known to involve metabolites produced by NATs, the phase II enzymes of xenobiotic metabolism.

**Fig. 4:**
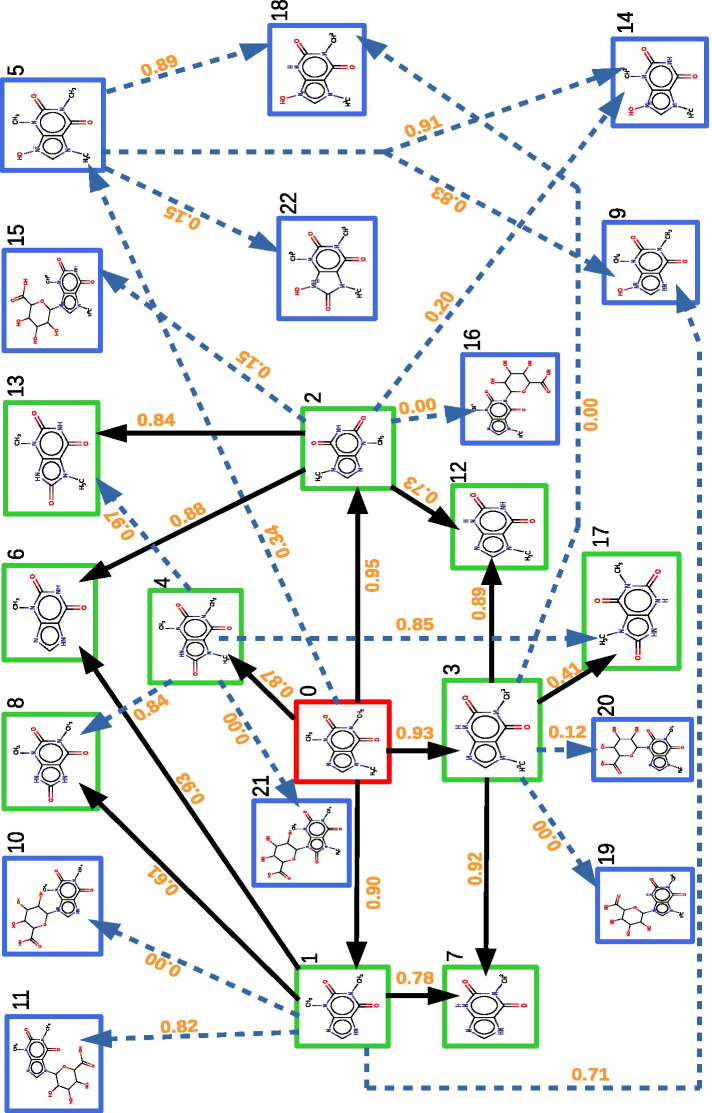
Map of metabolism of caffeine predicted by the tools SyGMa. Among the 23 metabolites, the caffeine node is shown in red and metabolites which have been experimentally observed in previous studies are shown in green. Among the 31 reactions depicted by arrows, the 13 black arrows depict transformations with literature-based evidence. Site of metabolism (SOM) score annotating reactions are shown in orange bolt on arrows. They are provided by SOM prediction tools (Way2Drug SOMP, FAME 3). The figure is available in a plain page format in Additional file 3

Figure 4 shows maps of metabolism obtained as several steps of the pipeline were applied to the caffeine molecule, modelled by its SMILES formula extracted from pubchem [39]. The first step of the pipeline consisted of the prediction of caffeine metabolites according to two transformation steps using SyGMa [22]. SyGMa can make chemical structures predictions using two parameters that define a scenario, for more information refer to the methods section. We chose to use all sets of reactions available in SyGMa that are related to xenobiotics metabolism. We also chose to set the number of SyGMa iteration at 2 due to the fact that the first two reactions of xenobiotics metabolism [2] (phase I and phase II) are the main biotransformation steps. The resulting map contains 23 metabolites and 31 reactions shown in Fig. 4.

Predicted metabolites Our results are consistent with the knowledge in the literature [42–46], considering that 11 of the 16 known metabolites of caffeine, including caffeine itself, are effectively recovered according to two steps of reactions associated with phase I and phase II reactions. These metabolites are shown as green in Fig. 4, except for caffeine which is red in the figure. Only two metabolites, 5-acetylamino-6-formylamino-3-methyluracil, i.e., AFMU, and 6-amino-5-(N-formylmethylamino)-1,3-dimethyluracil, i.e., 137-TAU, are not identified and are both associated with a NAT catalysed reaction. The other known metabolites of caffeine, such as 3-methyluric acid (i.e., 3MU), 7-methyluric acid (i.e., 7MU), and 1-methyluric acid (i.e., 1MU), are out of the scope of the method because they can not be predicted with chosen parameters (number of reaction steps).

The method predicted 16 reactions between known metabolites (green nodes), including the 13 reactions supported by the literature (black arrows) [42, 45, 46]. Therefore, our method predicted three new possible transformations from 137U (1,3,7-trimethyluric acid, node 4 in the figure) to 13U (node 8), 17U (node 17) and 37U (node 13), through a demethylation. The latter is similar to other reactions of the model. This suggests that the metabolite 137U could be transformed into several metabolites although this hypothesis has not been tested because of the low quantities produced in human metabolism and its elimination [42].

In addition to 11 known metabolites of caffeine, the method predicted that 12 other compounds are potential caffeine metabolites (blue nodes). Indeed the metabolite node 5 ( Cn1c(=O)c2c(n(C)c1=O)[n+](O)cn2C ) is predicted to be derived from caffeine after one step reaction. Ten other compounds are predicted to be derived from caffeine after two step reactions. Among these ten metabolites, three (nodes 9, 14, 18) are also predicted to be metabolites of node 5. A single metabolite (node 22) is predicted to be derived only from the newly metabolite node 5, which has the specificity to lose the aromatic structure of the imidazole.

Production probability scores of predicted metabolites In order to estimate the confidence of the predicted metabolites, we computed production probability scores for each of them. As detailed in the Methods section, the production probability score first takes into account scores associated with reactions, which are computed from several site of metabolism (SOM) scores predicted by dedicated tools (Way2Drug SOMP, FAME3), e.g. the chance that a transformation occurs on a given atom. In addition, the production probability score for the metabolites also takes into account the different pathways, e.g. chains of reaction, from caffeine to the considered metabolite, combined according to a Bayesian framework.

**Fig. 5:**
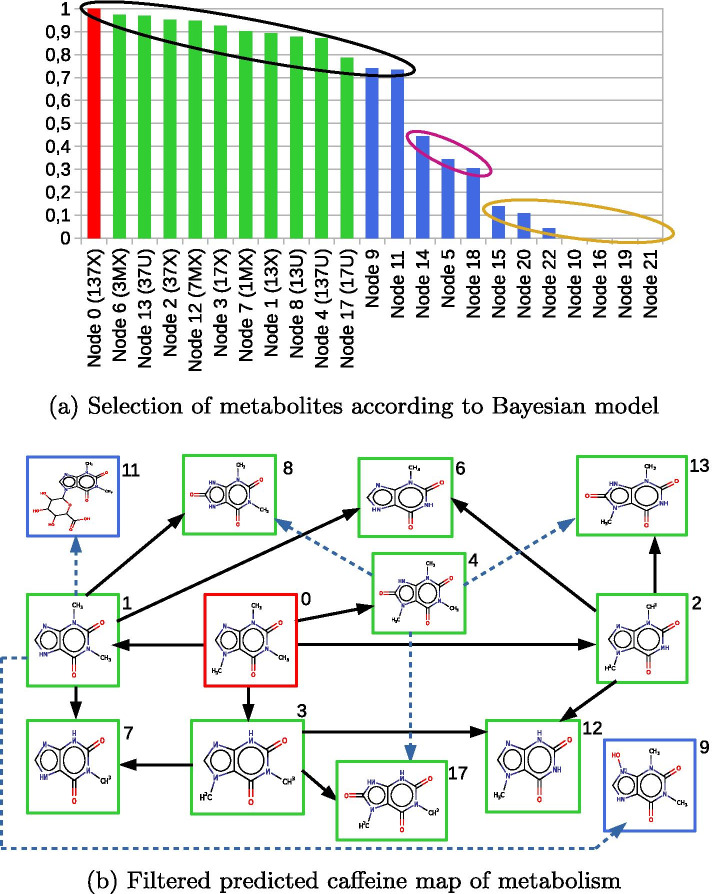
Filtered map of metabolism for caffeine. **a** Production probability score of predicted metabolites. The production probability score of each metabolite is computed according to a Bayesian model based on the reaction production probability scores. Red, green and blue bars are associated with caffeine, known and unknown metabolites, respectively. Metabolites are grouped into three groups according to their range of scores (black, red and yellow ellipses). **b** Filtered map of metabolism of caffeine. This map is the filtration of the previous predicted map in order to keep metabolites with a high production probability score (black ellipse, score > 0.70). The map contains 13 metabolites and 17 reactions. Two unknown metabolites (nodes 9 and 11) are predicted to be nearly as likely to be produced as the known metabolites according to the production probability scores. Each (a) and (b) figure are available in a plain page format in Additional file 4

The SOM score associated with reactions are indicated as an orange label on arrows in Fig. 4. We notice that five reactions have a null score. The production probability score for metabolites are shown in Fig. 5a, where metabolites are ordered from top-score metabolites to lowest scores.

The 11 known metabolites (green nodes) have the largest scores. The 12 unknown predicted metabolites are represented with a blue bar.

We observe that these predicted metabolites can be gathered into three groups, each of them is shown by an ellipse with a specific color. The first group (black ellipse) describes metabolites with a score greater than 0.70: it contains all known metabolites predicted by our method and two unknown metabolites, node 9 (Cn1c(=O)c2[nH]c[n+](O)c2n(C)c1=O) and node 11 (Cn1c(=O)c2c(ncn2C2OC(C(=O)O)C(O)C(O)C2O)n(C)c1=O). The second group (violet ellipse) contains three metabolites with a medium score (between 0.20 and 0.70) corresponding to the unknown metabolites node 14, node 5 and node 18. The last group of seven unknown metabolites (yellow ellipse) with low score (< 0.20) corresponding to the nodes 15, 20, 22, 10, 16, 19 and 21. The remaining nodes 10, 16, 19 and 21 have a score of 0.00, which is explained by the fact that all the pathways which produce them contain at least one reaction with a null score.

Filtered caffeine metabolic map The production probability scores were used to filter the caffeine map of metabolism as follows. The metabolites of the filtered map are all metabolites of the first group (black ellipse) in Fig. 5a, and the reactions in the map are those of Fig. 4 transforming nodes in the black ellipse group. The filtered map is shown in Fig. 5b. We observe that all the known 11 metabolites and 13 reactions belong to this map and therefore were conserved by the filtration procedure.

The filtering procedure removed metabolites associated with nodes 5, 14, 18 and 22, which have a specific configuration regarding nitrogen atoms on the imidazole part. These four metabolites are the only ones that are oxidized metabolites of caffeine with a pattern N=C-N or N-C-N such that one of the nitrogens is methylated and the other nitrogen is linked to an oxygen atom.

The other metabolites that were eliminated by the filtration procedures are nodes 10, 15, 16, 19, 20, and 21. They are all glucuronyl-conjugates of caffeine and caffeine metabolites whose glucoronyl group is not associated with the methylated nitrogen atom of the caffeine imidazole part. The fact that most of the glucuronyl conjugates are filtered is consistent with the literature because there is no glucuronyl-conjugate metabolite of caffeine described in humans yet. The only glucuronyl-conjugate appearing in the final map is node 11 (with SMILES formula : Cn1c(=O)c2c(ncn2C2OC(C(=O)O)C(O)C(O)C2O)n(C)c1=O, or IUPAC name: 6–(1,3–dimethyl–2,6–dioxo–2,3,6,7–tetrahydro–1*H*–purin–7–yl)–3,4,5 –trihydroxyoxane–2–carboxylic acid), whose glucoronyl group is linked to the imidazole part.

This suggests that node 1 (theophylline or 13x), which is already known to be metabolized into nodes 6 (3-methylxanthine or 3MX), 7 (1-methylxanthine or 1MX) and 8 (1,3-dimethyluric acid or 13U) could also have the potential to be biotransformed into another new metabolite. However the 13U and 1MX which are the most observed metabolites from 13X [42], could compete with this new metabolite and make it undetectable.

Apart from node 11, the only unknown metabolite of the initial map conserved after the filtration is node 9 (Cn1c(=O)c2[nH]c[n+](O)c2n(C)c1=O or 9–hydroxy–1,3–dimethyl–2,6–dioxo–2,3,6,7–tetrahydro–1*H* –purin–9–ium). According to our method, this metabolite is formed from node 1, e.g. 1,3-dimethylxanthine (theophylline or 13x), with a SOM score predicted by Way2Drug of 0.714 for enzyme CYP3A4. This reaction from node 1 is an oxidation on an nitrogen atom of the imidazole part. Contrary to the oxidation of the carbon atom between the nitrogen atoms in the imidazole part which produced node 8 (13U) from node 1 with a predicted SOM score of 0.606 for enzyme CYP2D6, with the same order of magnitude, this reaction occurs on an un-methylated nitrogen atom of the imidazole part. This suggests that the oxidation on nitrogen atoms of caffeine could theoretically occur although it has never been experimentally observed.

### Application to heterocyclic aromatc amines (HAA) and DNA reactivity predictions

The caffeine example suggests that there is an added value to building a map of metabolism by combining several approaches such as assembling reaction prediction tools, predicting sites of metabolism and filtering the map according to a production probability score. Based on this validation, we have further investigated how this method may facilitate the prediction of DNA adducts formation derived from xenobiotics. In this vein, we first constructed the predicted maps of metabolism of the 30 human HAA (see Additional file 5for details). Afterwards, we annotated maps of metabolism for six HAA of interest in order to study the prediction of DNA adduct formation.

Unfiltered maps of metabolism of HAA The pipeline was applied to predict maps of metabolism of the 30 identified HAA. The characteristics of the maps predicted by the tool SygMa are described in Table 1. HAA are ordered according to the number of metabolites in the maps predicted by the SyGMa tool. HAA associated with the largest map are 4-CH_2_OH-8-MeIQx and 4,7,8-TriMeIQx (194 predicted metabolites). The smallest maps correspond to the HAA Harman (70 metabolites) and norharman (50 metabolites). The size of these maps could be explained by the chemical structure of both HAA that have few sub-structures on which SyGMa transformation rules can be applied. The maps predicted for two of the three well characterized HAA in primary human hepatocytes [12, 13, 15], MeIQx—155 metabolites—and PhIP—128 metabolites, have a medium size. On the contrary, A$\alpha$C, the third one, is associated with one of the smallest maps of metabolism.

**Table 1:** Characteristics of the maps of metabolism predicted according to biotransformations rules by the SyGMa tool for 30 HAA

HAA	Metabolites	Reactions	Metabolites which are reactive to DNA	Ratio of metabolites reactive to DNA
4,7,8-TriMeIQx	194	282	91	46.9
4-CH_2_OH-8-MeIQx	189	266	80	42.3
7,8-DiMeIQx	174	250	81	46.6
7,9-DiMeIgQx	174	250	81	46.6
4,8-DiMeIQx	174	250	79	45.4
6,7-DiMeIgQx	169	245	76	45.0
AMPNH	157	225	16	10.1
7-MeIgQx	155	220	70	45.2
MeIQx	155	220	70	45.2
GluP1	142	202	64	45.1
IQx	137	192	62	45.3
IgQx	133	188	56	42.1
TrP1	129	179	61	47.3
PhIP	128	177	57	44.5
MeIQ	125	175	64	51.2
4’-OH-PhIP	123	166	49	39.8
3,5,6-TMIP	122	172	57	46.7
APNH	120	165	10	8.3
MeA$\alpha$C	113	154	48	42.5
TrP2	113	154	49	43.4
GluP2	110	151	46	41.8
IQ	109	150	59	54.1
IQ[4,5-*b*]	109	150	59	54.1
1,5,6-TMIP	107	153	59	55.1
1,6-DMIP	95	132	53	55.8
IFP	90	123	37	41.1
A$\alpha$C	85	111	33	38.8
PheP1	76	97	35	46.1
Harman	70	98	9	12.7
norharman	50	65	0	0.0
Average	127,6	178.7	53.7	41.0
Median	124	173.5	58	45.2

We also noticed that the maps associated with pairs of HAA such as 7,8-DiMeIQx and 7,9-DiMeIgQx, MeIQx and 7,MeIgQx or IQ and IQ[4,5-*b*] are associated with maps with similar characteristics: they have the same number of metabolites, reactions and metabolites reactive to DNA. The closeness of the chemical structure of isomers could explain such a similarity between maps: as planar isomers are composed of the same chemical substructures, the transformation rules contained in the SyGMa database have a high probability to occur equivalently on the metabolites of both HAA.

For each HAA, we estimated the number of metabolites reactive to DNA by assuming that each metabolite with a *XenoSite Reactivity* score greater than 0.85 is reactive to DNA, following the criteria introduced in [36]. The four HAA, 4-CH_2_OH-8-MeIQx, 7,8-DiMeIQx, 7,9-DiMeIgQx and 4,7,8-TriMeIQx have both the largest map and the greatest amount of metabolites reactive to DNA (91, 80, 81 and 81). norharman characterized by the smallest map contains no metabolite reactive to DNA. Harman, AMPNH and APNH are also associated with a very small ratio of metabolites reactive to DNA (12.7%, 10.1% and 8.1%). The ratio for the other HAA ranges from 38.8 to 55.8%. MeIQx and PhIP have a relatively high ratio of metabolites reactive to DNA (45.2% and 44.5%) while A$\alpha$C has a lower ratio (38.8%) in spite of its known higher reactivity towards DNA compared with MeIQx or PhIP [15]. This observation might be related to its smallest map of metabolism (average and median of numbers of metabolites and reactions in the map). Several hypotheses can be made about the variability of the sizes of the maps of metabolism: (a) the metabolites reactive to DNA do not have the same importance *in vitro*, (b) the reaction predictions performed by SyGMa may be incomplete, as we observed it for NAT2 reactions in the case of caffeine, (c) the reactions performed by SyGMa may not be homogeneous. As detailed below, the analysis of the production probability scores of the maps suggests that the last two hypotheses are highly probable.

Manual annotation and filtering of six maps of metabolism As the pipeline for the study of maps of metabolism encompasses a part of manual annotations for atom number of reactions, we applied the pipeline to a selection of six HAA among the 30 HAA. We first selected A$\alpha$C, PhIP and MeIQx, three HAA which are well described in human hepatocytes [12–15]. We complemented this list with the two HAA with the largest map (4-CH_2_OH-8-MeIQx and 4,7,8-TriMeIQx). We finally selected 7,8-DiMeIQx which represents the pair of isomers (7,8-DiMeIQx and 7,9-DiMeIgQx) having large maps. Note that the three selected HAA (4-CH_2_OH-8-MeIQx, 4,7,8-TriMeIQx and 7,8-DiMeIQx) have also the largest number of metabolites reactive with DNA. We applied the prediction pipeline and the annotated maps of metabolism obtained were explored to identify enzyme families and isoforms associated with reactions. We confirmed that most enzyme families (SULTs, NATs, CYPs, UGTs) and CYPs isoforms (CYP1A2, CYP2C19, CYP2C9, CYP2D6 and CYP3A4) annotate at least one reaction in each map with the exception of GSTs.

The annotated maps of metabolism obtained from A$\alpha$C, PhIP and MeIQx were explored in order to identify the metabolites corresponding to the metabolites described in humans of these three HAA (see Additional file 6for more details). Upon the 11, 10 and 9 metabolites experimentally shown for A$\alpha$C, MeIQx and PhIP, respectively, 9, 6 and 7, respectively are found in the annotated maps of metabolism. Among the 8 known metabolites not present in annotated maps of metabolism, three are N-sulfonyl metabolites of each HAA and three are N-acetoxy metabolites of each HAA. This suggests that N-sulfonyl and N-acetoxy metabolites may not be predicted using SyGMa’s SMIRKS rules, supporting the hypothesis (b) above. The last two known metabolites of MeIQx, 7-oxo-MeIQx and N-desmethyl-7-oxo-MeIQx are not present in annotated maps of metabolism but we identified two metabolites, with SMILES formula Cc1nc2c(ccc3c2nc(N)n3C)nc1O and Cc1nc2c(ccc3[nH]c(N)nc32)nc1O, which are close to these known missing metabolites. The main structural difference between those metabolites and 7-oxo-MeIQx and N-desmethyl-7-oxo-MeIQx is that the ketone group of the 7-oxo-MeIQx part is replaced by a hydroxyl group. This result suggests also that SyGMa’s SMIRKS rules are not able to predict the ketone group linked to a carbon of a heterocycle, which still supports the hypothesis (b) above.

**Fig. 6:**
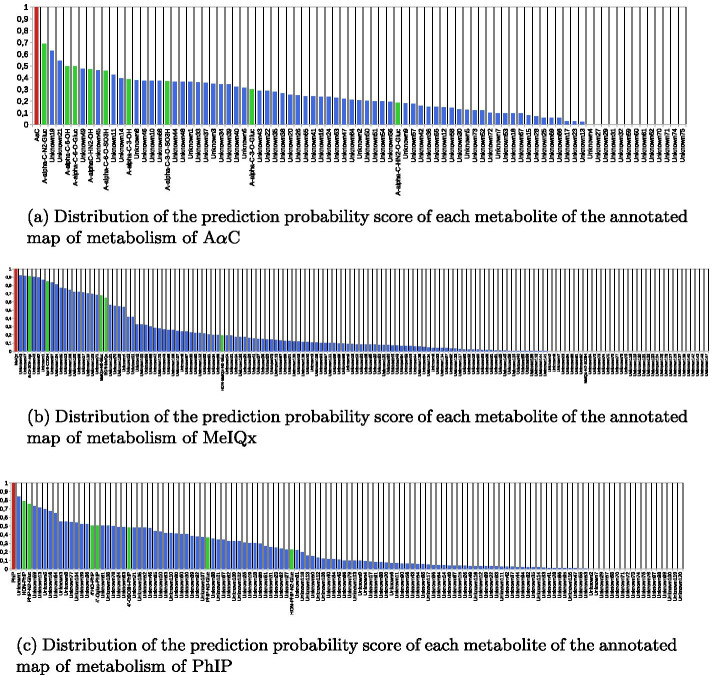
Distribution of prediction probability scores in annotated maps of metabolism. Distribution of prediction probability score of A$\alpha$C (**a**), MeIQx (**b**) and PhIP (**c**). The original compound of each map is shown with a red bar. Metabolites which have been experimentally observed are indicated by a green bar and other metabolites are indicated with a blue bar. The names of metabolites shown in the X-axis are either the name found for known metabolites or a two parts name: *Unknown* + *X* where X describes the identifier of the node in the map associated with this metabolite. All elements of this figure are available in Additional file 7in full page format

Figure 6a–c, show that the distribution of the production probability scores of the known metabolites of each HAA is rather scattered with scores ranging from 0.9 to 0.2. Surprisingly, the metabolite MeIQx-N^2^-SO3H, in the map of metabolism of MeIQx, is the only experimentally identified metabolite associated with a production probability score of 0.0. A null score suggests that the metabolic pathways leading to the metabolite contains at least one reaction that has not been annotated either by Way2Drug for a reaction of rank 1, or by FAME 3 for a reaction of rank 2.

In general, we notice that PhIP and MeIQx have a high amount of metabolites associated with a low production probability score, compared to A$\alpha$C. 83 metabolites (53.5% of the map) of the MeIQx map have a score lower than 0.1, including 36 metabolites with a nul score (23.2% map). Similarily, 65 metabolites (50.8% of the map) of the PhIP map have a score lower than 0.1 including 18 metabolites with a nul score (14.1% map) and no known metabolite. On the contrary, only 26 metabolites (30.6% of the map) of the A$\alpha$C map have a score lower than 0.1, including 13 metabolites with a nul score (15.3% map). Based on this remark, and after comparing the effect of different thresholds, we used the value 0.10 to filter metabolites with little support according to our model.

Table 2 describes the characteristics of the six selected filtered maps of metabolism. Details about metabolites contained in these maps are provided in Additional file 8 and the 6 maps are shown in Additional files 9–14 We observe that the filtration procedure has a strong impact on sizes of the maps of metabolism. 126 metabolites derived from 4-CH_2_OH-8-MeIQx map of metabolism are filtered representing 66% of the map and more than half of metabolites are filtered in the maps of PhIP, MeIQx, 7,8-DiMeIQx and 4,7,8-TriMeIQx. By contrast, the filtration procedure had a lower impact on A$\alpha$C since only 26 metabolites are filtered. This suggests that many reactions which were predicted according to existing transformation rules were currently not supported by sites of metabolism.This over-approximation is consistent with the fact that methods predicting metabolites using rule-based approaches are known to generate a large number of potential metabolites [23, 47].

We observe that the filtering procedure based on prediction probability scores tends to homogenize the size of the final maps of metabolism while keeping a similar ratio of metabolites predicted to be reactive to DNA (between 40 and 50%). This suggests that there is a strong interest in using SOM scores (as included in the production probability scores) to homogenize maps of metabolism and eliminate unsupported DNA reactive metabolites.

**Table 2:** Characteristics of six HAA maps of metabolism filtrated according to production probability scores computed after the annotation of all metabolites of each map

HAA	Metabolites after filtration	Filtered metabolites	Reactions after filtration	Filtered reactions	DNA reactive metabolites after filtration	Filtered DNA reactive metabolites
4,7,8-TriMeIQx	87	107	120	162	41	50
7,8-DiMeIQx	90	84	116	134	43	38
MeIQx	72	83	90	130	27	43
PhIP	63	65	74	103	22	35
4-CH2OH-8-MeIQx	63	126	78	188	31	49
AaC	59	26	72	39	20	13

Optimal enzymatic signature in terms of DNA reactivity As described previously, the pipeline relies on the computation of SOM scores on reactions involving manually annotated metabolites to reduce the maps of metabolism according to a Bayesian prediction probability. As SOM scores are directly related to enzymes, the production probability score is influenced by enzyme availability. We define *enzymatic contexts* to be tables describing all the possible combinations of enzymes that may be considered to be available. In our study, there are 512 such different enzymatic contexts. Each enzymatic context is associated with a specific distribution of production probability scores. Indeed, when an enzyme is described as *unavailable* in an enzymatic context, the reactions annotated with this enzyme cannot be taken in account for the calculation of the production probability scores.

Based on this assumption, for each of the six filtered maps of metabolism described above, and for each of the 512 enzymatic contexts, we computed the production probability scores of all metabolites of the map. This allowed us to determine the *global reactivity score of an HAA in a given context* that we defined as the sum of the production probability score (in the considered context) of all metabolites reactive to DNA in the considered map of metabolism.

In this framework, it becomes possible to define *optimal enzymatic signatures in terms of reactivity*, which are all the enzymatic contexts where the *global reactivity score* is maximized while they contain the smallest number of activated enzymes. Intuitively, optimal enzymatic signatures therefore correspond to enzymatic contexts where the chance to obtain at least one metabolite reactive to DNA is maximal according to our models.

**Fig. 7:**
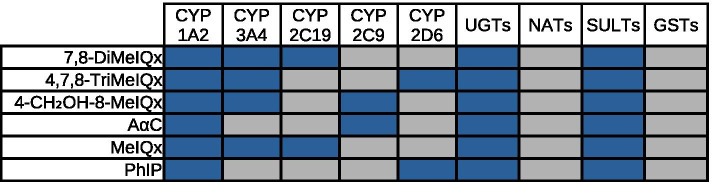
Optimal enzymatic signatures in terms of reactivity. A blue cell corresponds to an available enzyme. A grey cell corresponds to an unavailable enzyme. The threshold to determine if a metabolite is reactive to DNA was 0.85

Impact of enzymatic context to the production probability score and application on DNA adduct formation of HAA Fig. 7 shows all optimal enzymatic signatures for the six HAA.

We observe that the enzymes UGTs, SULTs and CYP1A2 are present in all optimal signatures of the six HAA. This is consistent with the literature which describes the implication of SULTs and CYP1A2 in the formation of HAA DNA adducts [2, 48]. In addition, it has been recently shown that UGTs are also involved in a pathway leading to DNA adduct formation for A$\alpha$C [15].

Conversely, the enzymes NATs and GSTs are absent in all optimal signatures. While the absence of GSTs is explained by the fact that annotated maps of metabolism do not contain any GSTs, the absence of NATs suggest that the resulting metabolites are not reactive to DNA since NATs are present in all maps of metabolism. However, NAT2 has been previously involved in formation of DNA adducts derived from HAA [2, 48]. We hypothesise that NATs reaction implicates other isoforms in the maps of metabolism. In accordance with this hypothesis, NAT2 enzymes did not appear in the map of metabolism predicted for caffeine since it is involved in the only known missing metabolite (AFMU).

Figure 7 also suggests that HAA is characterized by a specific profile of enzymes. For example, CYP3A4 belongs to the signature of four HAA which all have a chemical structure close to that of MeIQx.. This suggests that the involvement of CYPs, other than CYP1A2, in the formation of reactive metabolites depends on the chemical structure of HAA.

**Fig. 8:**
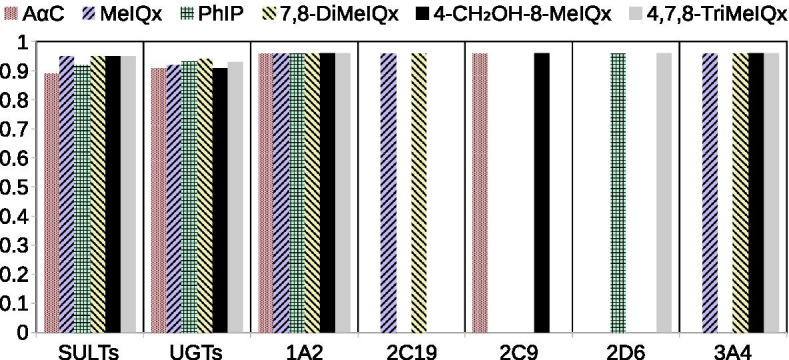
Optimal enzymatic signatures in terms of reactivity for each reactivity score thresholds. Each line describes an optimal enzymatic signature for each HAA for a reactivity score threshold

Impact of the XenoSite Reactivity threshold In order to test the impact of the reactivity threshold chosen to characterize all the compounds of a DNA reactive map, we decided to explore all the thresholds from 0 to 1.0. For the 101 threshold values considered (step of 0.01), we recalculated all the metabolites considered to be “reactive” (i.e. associated with a XenoSite Reactivity score greater than or equal to the threshold), and then calculated the optimal signatures for reactivity to DNA. According to our previous results we did not consider NATs and GSTs. As shown in Fig. 8, the enzymes CYP1A2, UGTs and SULTs are present in the optimal signature whatever the threshold thereby suggesting that the result described in Fig. 7 for the reactivity threshold value 0.85 is robust. Figure 8 also confirms the specificity of CYP3A4 in optimal signatures of HAA with an MeIQx chemical structure.

We further observed a low variability between HAA since the largest optimal signature is reached at a reactivity threshold of 0.89 for A$\alpha$C, 0.91 for 4-CH_2_OH-8-MeIQx, 0.92 for MeIQx and PhIP, 0.93 for 4,7,8-TriMeIQx and 0.95 for 7,8-DiMeIQx. In addition, the use of any reactivity threshold lower than 0.89 returns the same optimal signature for each HAA. This suggests that enzymes involved in metabolic pathways leading to the most reactive metabolites (given by XenoSite Reactivity scores), are sufficient for activating all the pathways leading to the less reactive metabolites.

**Fig. 9:**
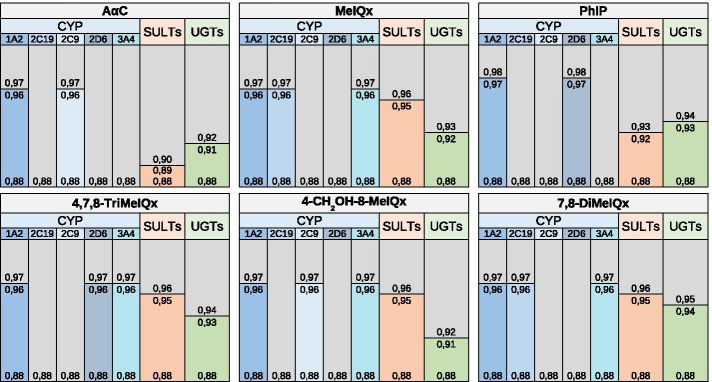
Optimal signatures in terms of reactivity for the highest reactivity thresholds. A grey cell means that the enzyme is not a part of the optimal signature and a colored cell means that the enzyme is *available* in the optimal signature. Each line represents the optimal signature at a specific reactivity score threshold. Thresholds marking the integration of new enzymes in the optimal signature are annotated on each bar

Figure 9 is the counterpart of Fig. 8 to compare the values of XenoSite reactivity associated with the appearance of each enzyme in an optimal signature. When considering high XenoSite Reactivity thresholds, we observed that cytochromes P450 isoforms are the only enzymes present in all optimal signatures (thresholds from 0.96 to 0.97). This suggests that CYPs are responsible for the production of most of DNA-reactive metabolites, especially CYP1A2 found in all HAA. Therefore, reactive metabolites derived from CYPs-annotated reactions may form DNA adducts more easily than reactive metabolites derived from phase II enzyme annotated reactions. In addition, we have observed that the enzymes UGTs and SULTs were present in the optimal signatures for reactivity thresholds between 0.95 and 0.89. For MeIQx, 4-CH_2_OH-8-MeIQx, 7,8-DiMeIQx and 4,7,8-TriMeIQx, we noted that the reactivity thresholds for which SULTs is present in the optimal signature is greater than the one for UGTs. This suggests that SULTs-associated metabolites are more likely to form DNA adducts than UGTs-associated metabolites when the chemical structure of the HAA is close to the MeIQx structure.

## Discussion and conclusion

In this study, we introduced a pipeline for predicting the metabolism of xenobiotics in humans. The pipeline was applied to six HAA of interest as well as caffeine. Our prediction pipeline is based on the construction of so-called *enriched maps of metabolism*. The main specificity of the pipeline is to use a *production probability score* to sort metabolites according to both the prediction of site of metabolisms and the topology of the maps predicted by bio-transformation rules. This score allows for the comparison of metabolite production in different physiopathological conditions which permit the exploration of the role of enzymes in the production of specific metabolites. In this study, we focus on metabolites and their DNA adduct formation capacity.

The pipeline was used to reconstruct the maps of caffeine metabolism and of six HAA. Among the four xenobiotics for which the metabolism was known i.e;, MeIQx, PhIP, A$\alpha$C and caffeine, the majority of the metabolites described in humans were found. Among these known metabolites, not one was removed from the maps after the filtration on the production probability scores, with the exception of MeIQx-N^2^-SO3H.

In the maps of metabolism predicted by our pipeline, most experimentally identified caffeine and HAA metabolites are associated with a high prediction probability score. On the contrary, a large number of predicted metabolites, which can be considered as over-predicted metabolites, are not supported by site of metabolism predictions.

This fact allowed us to use prediction probability scores as filters that importantly reduced the size of the maps. In the case of HAA, half of the predicted metabolites were filtered out. In this way, maps of metabolism produced by other tools could be filtered by this production probability score.

The prediction of the map of caffeine and HAA metabolism highlighted areas for improvement in our pipeline. First, the study of the caffeine map of metabolism evidenced that a metabolite (AFMU), resulting from a NAT2-directed reaction, is not predicted by the SyGMa tool, suggesting that the tool is incomplete in predicting reactions catalyzed by N-acetyl transferases. This assumption is also supported by the analysis of the optimal HAA signatures as detailed in Fig. 7. The prediction of HAA maps of metabolism also suggested lacks of prediction of N-Sulfonyl and N-Acetoxy metabolites of A$\alpha$C, PhIP and MeIQx, which were not predicted because there are no SMIRKS rules in SyGMa adapted to the prediction of these metabolites. These different unpredicted metabolites advocate for the addition of new SMIRKS biotransformation rules to SyGMa’s predictions rules in order to complete the maps of metabolism with relevant enzymes.

We designed our methods for the study of HAA. As presented in Fig. 1 the metabolism of HAA is basically divided into two phases of reactions. This is why we choose to make two iterations of SyGMa to predict metabolites obtained by the two phases. However, it could be relevant to use a third iteration of the rules used in the paper when applying the method to another metabolite than HAA. In addition, we notice that the metabolic map of HAA could be extended with the prediction of chemical compounds able to bind to DNA by covalent binding. This was impossible because SyGMa does not contain the corresponding SMIRKS rules. However, we notice that SyGMa is a flexible tool that allows the user to add SMIRKS rules to predict other metabolites; it could therefore be possible to enrich the metabolic map by adding to SyGMa the SMIRKS rules associated with DNA binding and extend the maps with a third iteration of the method to identify DNA adducts of HAA.

A characteristic of the map of metabolism of MeIQx is that MeIQx-N^2^-SO3H, an experimentally identified metabolite, is associated with a nul prediction probability score. This is explained by the fact that the reaction producing MeIQx-N^2^-SO3H is labeled as a rank 1 reaction, catalyzed by the SULTs. Our pipeline differentiate rank 1 and rank 2 reactions and could not annotate reactions of rank 1 with a SOM score only available for tools annotating reactions of rank 2. To overcome this issue, we plan on differentiating the SOMs prediction tools according to the enzymes annotating the reactions instead of the rank of the reactions. This, however will require the homogenization of the level of information about isoforms.

The production probability score that we defined allowed us to analyze the influence of enzymes on the production of DNA reactive metabolites and to propose a specificity of the CYP3A4 enzyme in the production of DNA adducts derived from AHAs close to MeIQx, which will be the subject of further experiments.

In conclusion, our study describes a new method for the construction and analysis of maps of metabolism by combining prediction of biotransformation rules, predictions of site of metabolisms, and prediction of reactivity to DNA. The method was validated and applied to six xenobiotics. Further study will consist of applying the pipeline to the 24 other human HAA, which requires the automatization of the annotation of metabolites predicted by transformation rules. Moreover, our approach based on enzymes profile determination opens the perspective to predict various xenobiotics derived metabolites susceptible to bind to DNA adducts in both normal and physiopathological situations. The enzymatic contexts extracted from data repositories such as TCGA [49] and GTEX [50] make this goal achievable.

## Methods

### Tool for the prediction of edges and nodes of maps of metabolism: SyGMa

The SyGMa python package (Systematic Generation of potential Metabolites) [22] is a rule-based method to predict metabolite (e.g. derivative chemical compounds) from an input chemical compound. In the paper, the input compound was either caffeine or HAA. The method relies on an internal set of metabolic reactions (biotransformation rules) in SMIRKS format which can be applied to the input compound. If the input chemical structure of a SMIRKS reaction is detected in the compound, the reaction is applied and the resulting structure obtained is a predicted metabolite.

The first parameter required by SyGMa to make predictions is the set of SMIRKS reactions to use. In this paper, we used the two sets of SMIRKS reactions, named “phase I” and “phase II”, obtained by data mining the Metabolite Database [22] and corresponding to reactions involved in phase I and II metabolism of xenobiotics.

The second parameter required by SyGMa is the number of reactions (which we call *rank*) that separates the original compound and a metabolite. If this maximal rank number is greater than 1, the metabolites obtained after a first iteration are used as a source for new reactions to obtain second rank metabolites, until the maximal rank is reached. In the paper, the maximal rank number was equal to 2. This allows the reproduction of the main observed xenobiotic metabolism with a first reaction associated to phase I enzymes of xenobiotic metabolism and a second reaction that conjugates the oxidized metabolite by enzymes of phase II metabolism of xenobiotics.

### Tools for the prediction of sites of metabolisms (SOM)

We used tools for site of metabolism (SOM) prediction tools to annotate reactions that could be catalysed by phase I or phase II enzymes of xenobiotics metabolism. Each method used for predicting SOMs estimates the capability of an enzyme to react with an atom according to a reference database of reactions and their metabolites. Using these models, each method can evaluate each atom of a compound to see if it can be involved in a reaction similar to those described in the reaction database. This analysis results in a score for each atom of a compound that describes the probability to be transformed by each reaction. Therefore, the methods differ according to the parameters describing the atom configuration and the enzymes involved in the reactions (different enzyme family and/or isoforms).

In order to take advantage of the panel of existing methods, our procedure included different tools to annotate each edge (reaction) with a site of metabolism (SOM) prediction score depending on its *rank*, *enzyme label* and *atom number of reaction* (see results section).

Way2Drug SOMP [29] is used to predict SOMs associated with reactions applied to the original compound as source (first-rank reactions). This SOM predictor has different CYP models and can provide a SOM score for each atom of a compound for five of the main cytochrome isoforms: 1A2, 3A4, 2D6, 2C9 and 2C19. It also predicts SOM scores for the UGT enzyme family but does not specify any isoforms.

When input of a reaction is not the original compound, we used FAME 3 [31] tool, which provides SOM score for phase II enzymes of xenobiotics metabolism. We use specific models restrained to specific reactions catalyzed by the five enzyme families: N-acetyltransferase (NATs), Sulfotransferases (SULTs) and Glutathione S-transferases (GTSs), UDP-glucuronosyltransferase (UGTs) and cytochromes P450 (CYPs).

### Prediction of SOR

XenoSite Reactivity [10, 11] is used to annotate the DNA reactivity of each metabolite. Based on the SOM scores, this tool computes a score of reactivity (SOR) for each atom of the metabolite meaning the ability of the atom to bind DNA. It also relies on the atom configuration, described by molecular descriptors, and uses deep-learning to infer a model that predicts the probability of an atom to be involved in a specific set of reactions that are DNA-binding reactions. To determine if a metabolite is considered as reactive to DNA, we apply a threshold on the SOR scores if at least one SOR score of an atom of the metabolite is retained, the metabolite is considered to be reactive to DNA. We use the same threshold as in [36] where XenoSite Reactivity where a threshold of 0.85 was learned according to metabolites known to be reactive to DNA.

Scoring metabolites with a SOM-based pathway production probability score A specific method was designed to compute a probability for each node to be formed for each metabolite according to all the annotations of the reactions of the metabolic map of metabolism. The approach relied on the formalism of Bayesian networks [41] which are probabilistic graphical models which represent a set of variables and their conditional dependencies via a directed acyclic graph (DAG). Bayesian networks were used to predict the the production probability score of each metabolite of the metabolic map, assuming that all possible production pathways were contributing factors to this score.

The graph (DAG) on which probabilities were computed was derived from the map of metabolism according to two principles. (1) The first principle applies when several isoforms of the same enzyme can transform a compound to another. In this case, the total affinity of the compound with the enzyme family is approximated by the maximal isoform enzyme affinity. (2) The second principle applies when several members of different enzyme families are competing to produce the same target metabolite from different input metabolites. In this case, the recruitment of enzymes in reactions is assumed to follow an exclusivity rule: each enzyme family can catalyze the production of the targeted metabolite with at most one reaction.

**Fig. 10:**
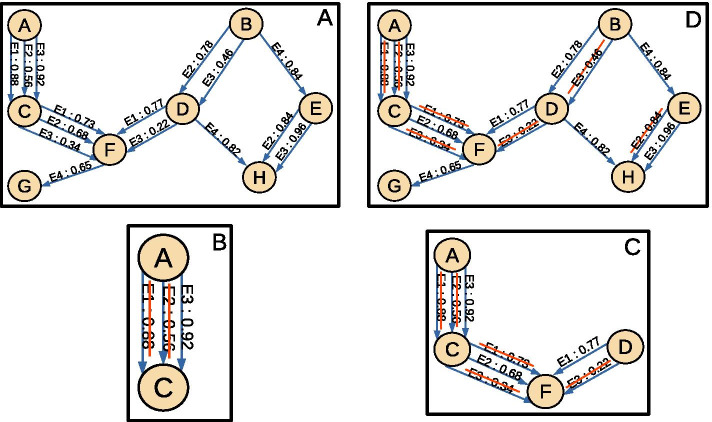
Creating a Bayesian network from an annotated map of metabolism. Considering **a** An annotated map of metabolism with enzymes named E1, E2, E3 and E4 and a SOM score associated to each enzyme for each reactions. **b** Illustrate the application of the rule: “only one enzyme can catalyze a reaction”. There is a reaction from A to C, divided in three reactions one for each enzyme annotating the reaction. Those reactions need to be reduced in one with a SOM score used as probability for the Bayesian Network. SOM score annotating reactions are indicators of enzymatic affinity so the reaction with maximal SOM score is selected. Here is the reaction annotated by E3. **c** Illustrate a different case where there are reactions from D and from C leading to production of F. This is the application of “if an enzyme is recruited by a reaction, another enzyme has to be recruited for a second reaction with the same product”. Here the maximal SOM score annotating reactions is 0.77 for the reaction from D to F annotated by E1. Because this reaction is the retain reaction from D to F, E1 is no more allowed to annotate the reduced reaction from C to F. The reaction retained from C to F, is the second reaction with the maximal SOM score which is the reaction annotated by E2. **d** Illustrate the final reduced graph used as Bayesian Network to calculate production probability score of metabolites

Consequently, a variable of the Bayesian model was built for each node (e.g. metabolites) of the map of metabolism. It was therefore associated with the event *production of the metabolite*. The DAG was built according to the following rules, which are illustrated in Fig. 10. (a) For reactions which have the same input and output and therefore varied only by the isoform of their enzyme family (in our case, CYPs), we selected the edge with the maximal SOM score and removed the other edges of the graph. (b) For each metabolite which is the output of several edges with different input, we selected the enzyme family (UGTs, NATs, SULTs, CYPs, GSTs) with the largest SOM score and removed all the other edges producing the targeted metabolite with the same enzyme family. We then selected the remaining enzyme family producing the targeted metabolite with the second largest score and again removed from the graph all the edges leading to the targeted metabolite with an enzyme of the same enzyme family. This was repeated until each enzyme family could produce the targeted metabolite from at most an input compound.

For each edge, the SOM score was interpreted as a conditional probability, which is the probability of getting the output compound of the edge assuming the presence of input compound. The structure of the graph, which is both acyclic (because the pipeline for building metabolic maps cannot create cycles) and such that enzymes do not compete for the production of the same metabolite, yields that conditional probabilities are independent. This allowed creating a complete probability table associated with each metabolite production event.

The *production probability score* of each metabolite was defined to be the probability of a metabolite production. These scores were computed by using the conditional probability tables to expand joint probability function (Bayes formula) [41].

## Supplementary information

**Additional file 1.**: Extended Figure 2 The file provides the figure 2 (a) and (b) in a plain page to make structures and text more readable.

**Additional file 2.**: Dictionary of SMIRKS rules The file provides a manually constructed catalogue of SMIRK rules required to map SMIRKS rules to enzyme family labels by taking into account the rank of the reaction in the pipeline for building enriched maps of metabolism.

**Additional file 3.**: Extended Figure 4 The file provides the figure 4 in a plain page to make structures and text more readable.

**Additional file 4.**: Extended Figure 5 The file provides the figure 5 in a plain page to make structures and text more readable.

**Additional file 5.**: Description of thirty HAA and Caffeine structure used as pipeline input This pdf file describes the SMILES formula and 2D-structure of each of the thirty HAA and the Caffeine studied in the paper.

**Additional file 6.**: Metabolites of A*α*C, PhIP and MeIQx founded in predicted metabolism maps The file provides a table describing for each metabolite experimentally described if they are predicted by SyGMa and if they are known to produce DNA adduct.

**Additional file 7.**: Distribution of 3 HAA production probability score The file provides the distribution of production probability scores used in Fig. 6 but in full page format.

**Additional file 8.**: Description of six HAA maps of metabolism The file provides a detailed description of metabolites for the six HAA filtered maps of metabolism built in the paper. For each metabolite of each map, the file provides the identifier of the metabolite, its SMILES formula, its production probability score, its reactivity to DNA and the score of XenoSite Reactivity.

**Additional file 9.**: Metabolism map of A*α*C A representation of the filtered metabolism map of A*α*C with chemical structures.

**Additional file 10.**: Metabolism map of MeIQx A representation of the filtered metabolism map of MeIQx with chemical structures.

**Additional file 11.**: Metabolism map of PhIP A representation of the filtered metabolism map of PhIP with chemical structures.

**Additional file 12.**: Metabolism map of 4,7,8-TriMeIQx A representation of the filtered metabolism map of 4,7,8-TriMeIQx with chemical structures.

**Additional file 13.**: Metabolism map of 4-CH_2_OH-8-MeIQx A representation of the filtered metabolism map of 4-CH_2_OH-8-MeIQx with chemical structures.

**Additional file 14.**: Metabolism map of 7,8-DiMeIQx A representation of the filtered metabolism map of 7,8-DiMeIQx with chemical structures.

## Data Availability

The description of HAA by SMILES formula were provided in [36]. The SMILES formula of caffeine was extracted from PubChem: https://pubchem.ncbi.nlm.nih.gov/. They are all available in the Additional file 5.
