# Supplementary material for: Constructing xenobiotic maps of metabolism to predict enzymes catalyzing metabolites capable of binding to DNA
Source: BMC Bioinformatics. 2021 Sep 21;22:450. doi: 10.1186/s12859-021-04363-6 (PMC8454073; doi:10.1186/s12859-021-04363-6)
Supplement: Supplementary file 1 — Additional file 1.: Extended Figure 2 The file provides the figure 2 (a) and (b) in a plain page to make structures and text more readable. [file 12859_2021_4363_MOESM1_ESM.pdf]

Constructing xenobiotic maps of metabolism to predict enzymes catalyzing metabolites capable of binding to DNA.

Conan M., Théret N., Langouet S. and Siegel, A

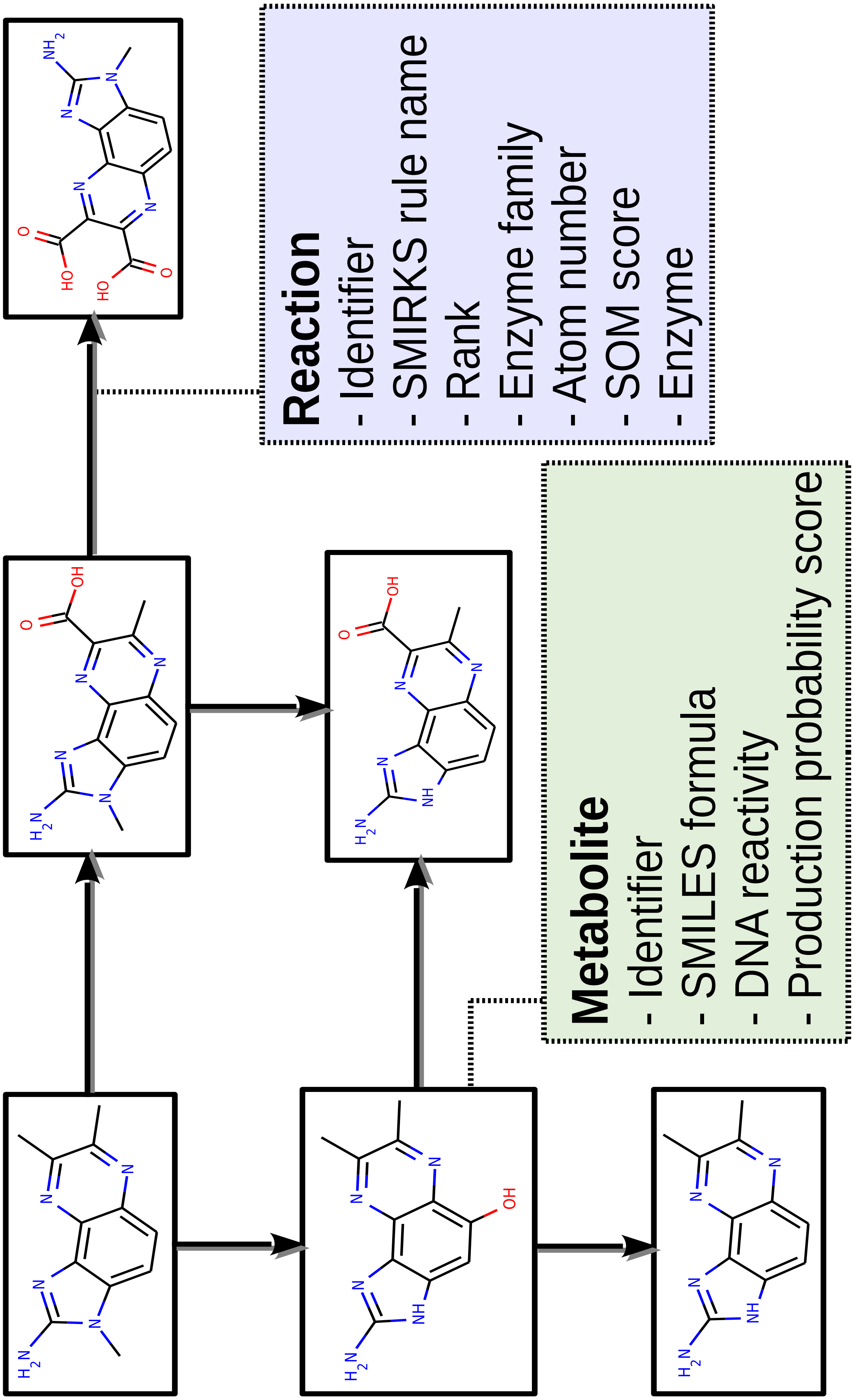

Enlarged version of Figure 2 (a) Metabolites are depicted by their 2D structure in black squares. Arrows between metabolites represent reactions, which consume a metabolite in order to produce another one. Left side: labels are shown in dashed squares (green for metabolites and blue for reactions

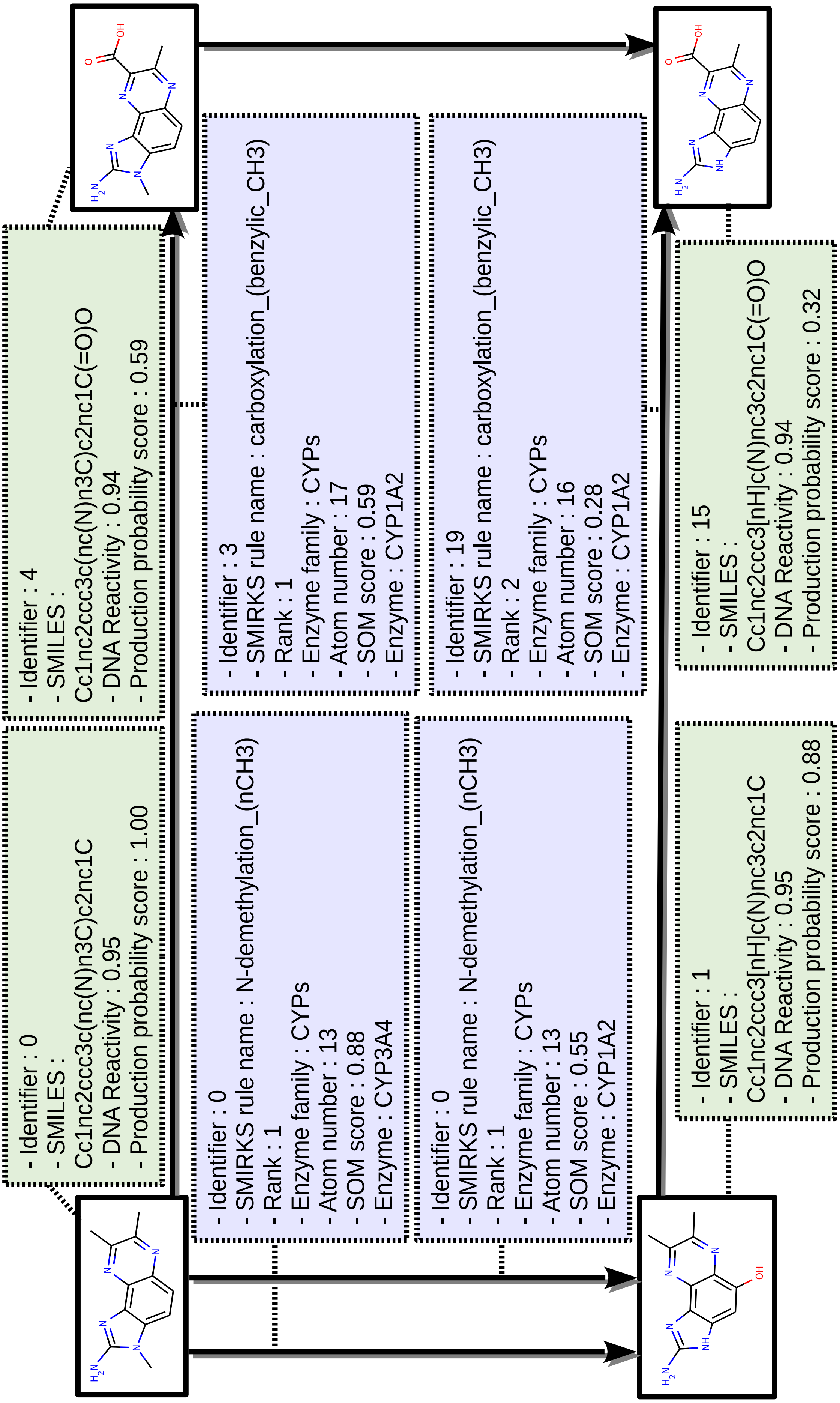

Enlarged version of Figure 2 (b) Examples of values of labels for a part of the above network.
