# Supplementary material for: Constructing xenobiotic maps of metabolism to predict enzymes catalyzing metabolites capable of binding to DNA
Source: BMC Bioinformatics. 2021 Sep 21;22:450. doi: 10.1186/s12859-021-04363-6 (PMC8454073; doi:10.1186/s12859-021-04363-6)
Supplement: Supplementary file 2 — Additional file 2.: Dictionary of SMIRKS rules The file provides a manually constructed catalogue of SMIRK rules required to map SMIRKS rules to enzyme family labels by taking into account the rank of the reaction in the pipeline for building enriched maps of metabolism. [file 12859_2021_4363_MOESM2_ESM.pdf]

Constructing xenobiotic maps of metabolism to predict enzymes catalyzing metabolites capable of binding to DNA

Conan M. , Théret N., Langouet S. and Siegel, A

Supplementary file 1.

The file provides a manually constructed catalogue of SMIRK rules required to map SMIRKS rules to enzyme family labels by taking into account the rank of the reaction in the pipeline for building enriched maps of metabolism.

Label of SyGMa SMIRKS Rule	Annotated Enzyme Family	Tool used	Annotated Enzyme
N-deacetylation	CYPs	Rank I reaction : Way2Drug SOMP	CYP1A2, CYP2C19, CYP2C9, CYP2D6, CYP3A4
		Rank II reaction : FAME 3	CYP1A2, CYP2C19, CYP2C9, CYP2D6, CYP3A4
N-dealkylation_(R-NHCH2-alkyl)	CYPs	Rank I reaction : Way2Drug SOMP	CYP1A2, CYP2C19, CYP2C9, CYP2D6, CYP3A4
		Rank II reaction : FAME 3	CYP1A2, CYP2C19, CYP2C9, CYP2D6, CYP3A4
N-dealkylation_(c-NHCH2-alkyl)	CYPs	Rank I reaction : Way2Drug SOMP	CYP1A2, CYP2C19, CYP2C9, CYP2D6, CYP3A4
		Rank II reaction : FAME 3	CYP1A2, CYP2C19, CYP2C9, CYP2D6, CYP3A4
N-dealkylation_(morpholine)	CYPs	Rank I reaction : Way2Drug SOMP	CYP1A2, CYP2C19, CYP2C9, CYP2D6, CYP3A4
		Rank II reaction : FAME 3	CYP1A2, CYP2C19, CYP2C9, CYP2D6, CYP3A4
N-dealkylation_(nCH2)	CYPs	Rank I reaction : Way2Drug SOMP	CYP1A2, CYP2C19, CYP2C9, CYP2D6, CYP3A4
		Rank II reaction : FAME 3	CYP1A2, CYP2C19, CYP2C9, CYP2D6, CYP3A4
N-dealkylation_(piperazine)	CYPs	Rank I reaction : Way2Drug SOMP	CYP1A2, CYP2C19, CYP2C9, CYP2D6, CYP3A4
		Rank II reaction : FAME 3	CYP1A2, CYP2C19, CYP2C9, CYP2D6, CYP3A4
N-dealkylation_(quarternary_N)	CYPs	Rank I reaction : Way2Drug SOMP	CYP1A2, CYP2C19, CYP2C9, CYP2D6, CYP3A4
		Rank II reaction : FAME 3	CYP1A2, CYP2C19, CYP2C9, CYP2D6, CYP3A4
N-dealkylation_(tertiaryN-CH2-alkyl)	CYPs	Rank I reaction : Way2Drug SOMP	CYP1A2, CYP2C19, CYP2C9, CYP2D6, CYP3A4
		Rank II reaction : FAME 3	CYP1A2, CYP2C19, CYP2C9, CYP2D6, CYP3A4
N-deformylation	CYPs	Rank I reaction : Way2Drug SOMP	CYP1A2, CYP2C19, CYP2C9, CYP2D6, CYP3A4
		Rank II reaction : FAME 3	CYP1A2, CYP2C19, CYP2C9, CYP2D6, CYP3A4
N-deglycosidation	CYPs	Rank I reaction : Way2Drug SOMP	CYP1A2, CYP2C19, CYP2C9, CYP2D6, CYP3A4
		Rank II reaction : FAME 3	CYP1A2, CYP2C19, CYP2C9, CYP2D6, CYP3A4
N-demethylation_(R-N(CH3)2)	CYPs	Rank I reaction : Way2Drug SOMP	CYP1A2, CYP2C19, CYP2C9, CYP2D6, CYP3A4
		Rank II reaction : FAME 3	CYP1A2, CYP2C19, CYP2C9, CYP2D6, CYP3A4
N-demethylation_(R-N(CR)CH3)	CYPs	Rank I reaction : Way2Drug SOMP	CYP1A2, CYP2C19, CYP2C9, CYP2D6, CYP3A4
		Rank II reaction : FAME 3	CYP1A2, CYP2C19, CYP2C9, CYP2D6, CYP3A4
N-demethylation_(R-NHCH3)	CYPs	Rank I reaction : Way2Drug SOMP	CYP1A2, CYP2C19, CYP2C9, CYP2D6, CYP3A4
		Rank II reaction : FAME 3	CYP1A2, CYP2C19, CYP2C9, CYP2D6, CYP3A4
N-demethylation_(c-N(CH3)2)	CYPs	Rank I reaction : Way2Drug SOMP	CYP1A2, CYP2C19, CYP2C9, CYP2D6, CYP3A4
		Rank II reaction : FAME 3	CYP1A2, CYP2C19, CYP2C9, CYP2D6, CYP3A4

N-demethylation_(c-NHCH3)	CYPs	Rank I reaction : Way2Drug SOMP	CYP1A2, CYP2C19, CYP2C9, CYP2D6, CYP3A4
		Rank II reaction : FAME 3	CYP1A2, CYP2C19, CYP2C9, CYP2D6, CYP3A4
N-demethylation_(nCH3)	CYPs	Rank I reaction : Way2Drug SOMP	CYP1A2, CYP2C19, CYP2C9, CYP2D6, CYP3A4
		Rank II reaction : FAME 3	CYP1A2, CYP2C19, CYP2C9, CYP2D6, CYP3A4
N-depropylation	CYPs	Rank I reaction : Way2Drug SOMP	CYP1A2, CYP2C19, CYP2C9, CYP2D6, CYP3A4
		Rank II reaction : FAME 3	CYP1A2, CYP2C19, CYP2C9, CYP2D6, CYP3A4
N-oxidation_(-N=)	CYPs	Rank I reaction : Way2Drug SOMP	CYP1A2, CYP2C19, CYP2C9, CYP2D6, CYP3A4
		Rank II reaction : FAME 3	CYP1A2, CYP2C19, CYP2C9, CYP2D6, CYP3A4
N-oxidation_(RN(CH3)2)	CYPs	Rank I reaction : Way2Drug SOMP	CYP1A2, CYP2C19, CYP2C9, CYP2D6, CYP3A4
		Rank II reaction : FAME 3	CYP1A2, CYP2C19, CYP2C9, CYP2D6, CYP3A4
N-oxidation_(aniline)	CYPs	Rank I reaction : Way2Drug SOMP	CYP1A2, CYP2C19, CYP2C9, CYP2D6, CYP3A4
		Rank II reaction : FAME 3	CYP1A2, CYP2C19, CYP2C9, CYP2D6, CYP3A4
N-oxidation_(tertiary_N)	CYPs	Rank I reaction : Way2Drug SOMP	CYP1A2, CYP2C19, CYP2C9, CYP2D6, CYP3A4
		Rank II reaction : FAME 3	CYP1A2, CYP2C19, CYP2C9, CYP2D6, CYP3A4
N-oxidation_(tertiary_NCH3)	CYPs	Rank I reaction : Way2Drug SOMP	CYP1A2, CYP2C19, CYP2C9, CYP2D6, CYP3A4
		Rank II reaction : FAME 3	CYP1A2, CYP2C19, CYP2C9, CYP2D6, CYP3A4
O-deacetylation	CYPs	Rank I reaction : Way2Drug SOMP	CYP1A2, CYP2C19, CYP2C9, CYP2D6, CYP3A4
		Rank II reaction : FAME 3	CYP1A2, CYP2C19, CYP2C9, CYP2D6, CYP3A4
O-dealkylation_(aliphatic)	CYPs	Rank I reaction : Way2Drug SOMP	CYP1A2, CYP2C19, CYP2C9, CYP2D6, CYP3A4
		Rank II reaction : FAME 3	CYP1A2, CYP2C19, CYP2C9, CYP2D6, CYP3A4
O-dealkylation_(aromatic)	CYPs	Rank I reaction : Way2Drug SOMP	CYP1A2, CYP2C19, CYP2C9, CYP2D6, CYP3A4
		Rank II reaction : FAME 3	CYP1A2, CYP2C19, CYP2C9, CYP2D6, CYP3A4
O-dealkylation_(methylenedioxyphenyl)a	CYPs	Rank I reaction : Way2Drug SOMP	CYP1A2, CYP2C19, CYP2C9, CYP2D6, CYP3A4
		Rank II reaction : FAME 3	CYP1A2, CYP2C19, CYP2C9, CYP2D6, CYP3A4
O-dealkylation_(methylenedioxyphenyl)b	CYPs	Rank I reaction : Way2Drug SOMP	CYP1A2, CYP2C19, CYP2C9, CYP2D6, CYP3A4
		Rank II reaction : FAME 3	CYP1A2, CYP2C19, CYP2C9, CYP2D6, CYP3A4
O-deglycosidation	CYPs	Rank I reaction : Way2Drug SOMP	CYP1A2, CYP2C19, CYP2C9, CYP2D6, CYP3A4
		Rank II reaction : FAME 3	CYP1A2, CYP2C19, CYP2C9, CYP2D6, CYP3A4
O-demethylation	CYPs	Rank I reaction : Way2Drug SOMP	CYP1A2, CYP2C19, CYP2C9, CYP2D6, CYP3A4
		Rank II reaction : FAME 3	CYP1A2, CYP2C19, CYP2C9, CYP2D6, CYP3A4
S-dealkylation_c-SCH2-R	CYPs	Rank I reaction : Way2Drug SOMP	CYP1A2, CYP2C19, CYP2C9, CYP2D6, CYP3A4
		Rank II reaction : FAME 3	CYP1A2, CYP2C19, CYP2C9, CYP2D6, CYP3A4
acetyl_shift	CYPs	Rank I reaction : Way2Drug SOMP	CYP1A2, CYP2C19, CYP2C9, CYP2D6, CYP3A4
		Rank II reaction : FAME 3	CYP1A2, CYP2C19, CYP2C9, CYP2D6, CYP3A4
aldehyde_oxidation_(aliphatic)	CYPs	Rank I reaction : Way2Drug SOMP	CYP1A2, CYP2C19, CYP2C9, CYP2D6, CYP3A4
		Rank II reaction : FAME 3	CYP1A2, CYP2C19, CYP2C9, CYP2D6, CYP3A4
aldehyde_oxidation_(aromatic)	CYPs	Rank I reaction : Way2Drug SOMP	CYP1A2, CYP2C19, CYP2C9, CYP2D6, CYP3A4

		Rank II reaction : FAME 3	CYP1A2, CYP2C19, CYP2C9, CYP2D6, CYP3A4
aldehyde_reduction_(aliphatic)	CYPs	Rank I reaction : Way2Drug SOMP	CYP1A2, CYP2C19, CYP2C9, CYP2D6, CYP3A4
		Rank II reaction : FAME 3	CYP1A2, CYP2C19, CYP2C9, CYP2D6, CYP3A4
aldehyde_reduction_(aromatic)	CYPs	Rank I reaction : Way2Drug SOMP	CYP1A2, CYP2C19, CYP2C9, CYP2D6, CYP3A4
		Rank II reaction : FAME 3	CYP1A2, CYP2C19, CYP2C9, CYP2D6, CYP3A4
aliphatic_dehalogenation	CYPs	Rank I reaction : Way2Drug SOMP	CYP1A2, CYP2C19, CYP2C9, CYP2D6, CYP3A4
		Rank II reaction : FAME 3	CYP1A2, CYP2C19, CYP2C9, CYP2D6, CYP3A4
aliphatic_hydroxylation_(primary_carbon_next_to_SP2_or_SP1)	CYPs	Rank I reaction : Way2Drug SOMP	CYP1A2, CYP2C19, CYP2C9, CYP2D6, CYP3A4
		Rank II reaction : FAME 3	CYP1A2, CYP2C19, CYP2C9, CYP2D6, CYP3A4
aliphatic_hydroxylation_(primary_carbon_next_to_quart_carbon)	CYPs	Rank I reaction : Way2Drug SOMP	CYP1A2, CYP2C19, CYP2C9, CYP2D6, CYP3A4
		Rank II reaction : FAME 3	CYP1A2, CYP2C19, CYP2C9, CYP2D6, CYP3A4
aliphatic_hydroxylation_(primary_carbon_next_to_sec_carbon)	CYPs	Rank I reaction : Way2Drug SOMP	CYP1A2, CYP2C19, CYP2C9, CYP2D6, CYP3A4
		Rank II reaction : FAME 3	CYP1A2, CYP2C19, CYP2C9, CYP2D6, CYP3A4
aliphatic_hydroxylation_(primary_carbon_next_to_tert_carbon)	CYPs	Rank I reaction : Way2Drug SOMP	CYP1A2, CYP2C19, CYP2C9, CYP2D6, CYP3A4
		Rank II reaction : FAME 3	CYP1A2, CYP2C19, CYP2C9, CYP2D6, CYP3A4
aliphatic_hydroxylation_(sec_carbon,next_to_CH3)	CYPs	Rank I reaction : Way2Drug SOMP	CYP1A2, CYP2C19, CYP2C9, CYP2D6, CYP3A4
		Rank II reaction : FAME 3	CYP1A2, CYP2C19, CYP2C9, CYP2D6, CYP3A4
aliphatic_hydroxylation_(sec_carbon_both_sides_next_to_SP2,in_a_ring)	CYPs	Rank I reaction : Way2Drug SOMP	CYP1A2, CYP2C19, CYP2C9, CYP2D6, CYP3A4
		Rank II reaction : FAME 3	CYP1A2, CYP2C19, CYP2C9, CYP2D6, CYP3A4
aliphatic_hydroxylation_(sec_carbon_in_a_ringA)	CYPs	Rank I reaction : Way2Drug SOMP	CYP1A2, CYP2C19, CYP2C9, CYP2D6, CYP3A4
		Rank II reaction : FAME 3	CYP1A2, CYP2C19, CYP2C9, CYP2D6, CYP3A4
aliphatic_hydroxylation_(sec_carbon_in_a_ringB)	CYPs	Rank I reaction : Way2Drug SOMP	CYP1A2, CYP2C19, CYP2C9, CYP2D6, CYP3A4
		Rank II reaction : FAME 3	CYP1A2, CYP2C19, CYP2C9, CYP2D6, CYP3A4
aliphatic_hydroxylation_(sec_carbon_next_to_SP2,in_a_ring)	CYPs	Rank I reaction : Way2Drug SOMP	CYP1A2, CYP2C19, CYP2C9, CYP2D6, CYP3A4
		Rank II reaction : FAME 3	CYP1A2, CYP2C19, CYP2C9, CYP2D6, CYP3A4
aliphatic_hydroxylation_(sec_carbon_next_to_SP2,not_in_a_ring)	CYPs	Rank I reaction : Way2Drug SOMP	CYP1A2, CYP2C19, CYP2C9, CYP2D6, CYP3A4
		Rank II reaction : FAME 3	CYP1A2, CYP2C19, CYP2C9, CYP2D6, CYP3A4
aliphatic_hydroxylation_(tert_carbon_linked_to_two_CH3_groups)	CYPs	Rank I reaction : Way2Drug SOMP	CYP1A2, CYP2C19, CYP2C9, CYP2D6, CYP3A4
		Rank II reaction : FAME 3	CYP1A2, CYP2C19, CYP2C9, CYP2D6, CYP3A4
aliphatic_hydroxylation_(tert_carbon_next_to_SP2)	CYPs	Rank I reaction : Way2Drug SOMP	CYP1A2, CYP2C19, CYP2C9, CYP2D6, CYP3A4
		Rank II reaction : FAME 3	CYP1A2, CYP2C19, CYP2C9, CYP2D6, CYP3A4
all_aliph_hydr	CYPs	Rank I reaction : Way2Drug SOMP	CYP1A2, CYP2C19, CYP2C9, CYP2D6, CYP3A4
		Rank II reaction : FAME 3	CYP1A2, CYP2C19, CYP2C9, CYP2D6, CYP3A4
all_dehydro	CYPs	Rank I reaction : Way2Drug SOMP	CYP1A2, CYP2C19, CYP2C9, CYP2D6, CYP3A4
		Rank II reaction : FAME 3	CYP1A2, CYP2C19, CYP2C9, CYP2D6, CYP3A4
aniline_to_nitro	CYPs	Rank I reaction : Way2Drug SOMP	CYP1A2, CYP2C19, CYP2C9, CYP2D6, CYP3A4
		Rank II reaction : FAME 3	CYP1A2, CYP2C19, CYP2C9, CYP2D6, CYP3A4

aromatic_dechlorination	CYPs	Rank I reaction : Way2Drug SOMP	CYP1A2, CYP2C19, CYP2C9, CYP2D6, CYP3A4
		Rank II reaction : FAME 3	CYP1A2, CYP2C19, CYP2C9, CYP2D6, CYP3A4
aromatic_dehydroxylation	CYPs	Rank I reaction : Way2Drug SOMP	CYP1A2, CYP2C19, CYP2C9, CYP2D6, CYP3A4
		Rank II reaction : FAME 3	CYP1A2, CYP2C19, CYP2C9, CYP2D6, CYP3A4
aromatic_hydroxylation_(meta_to_carbon)	CYPs	Rank I reaction : Way2Drug SOMP	CYP1A2, CYP2C19, CYP2C9, CYP2D6, CYP3A4
		Rank II reaction : FAME 3	CYP1A2, CYP2C19, CYP2C9, CYP2D6, CYP3A4
aromatic_hydroxylation_(ortho_to_2_substituents)	CYPs	Rank I reaction : Way2Drug SOMP	CYP1A2, CYP2C19, CYP2C9, CYP2D6, CYP3A4
		Rank II reaction : FAME 3	CYP1A2, CYP2C19, CYP2C9, CYP2D6, CYP3A4
aromatic_hydroxylation_(ortho_to_nitrogen)	CYPs	Rank I reaction : Way2Drug SOMP	CYP1A2, CYP2C19, CYP2C9, CYP2D6, CYP3A4
		Rank II reaction : FAME 3	CYP1A2, CYP2C19, CYP2C9, CYP2D6, CYP3A4
aromatic_hydroxylation_(ortho_to_oxygen)	CYPs	Rank I reaction : Way2Drug SOMP	CYP1A2, CYP2C19, CYP2C9, CYP2D6, CYP3A4
		Rank II reaction : FAME 3	CYP1A2, CYP2C19, CYP2C9, CYP2D6, CYP3A4
aromatic_hydroxylation_(para_to_carbon)	CYPs	Rank I reaction : Way2Drug SOMP	CYP1A2, CYP2C19, CYP2C9, CYP2D6, CYP3A4
		Rank II reaction : FAME 3	CYP1A2, CYP2C19, CYP2C9, CYP2D6, CYP3A4
aromatic_hydroxylation_(para_to_nitrogen)	CYPs	Rank I reaction : Way2Drug SOMP	CYP1A2, CYP2C19, CYP2C9, CYP2D6, CYP3A4
		Rank II reaction : FAME 3	CYP1A2, CYP2C19, CYP2C9, CYP2D6, CYP3A4
aromatic_hydroxylation_(para_to_oxygen)	CYPs	Rank I reaction : Way2Drug SOMP	CYP1A2, CYP2C19, CYP2C9, CYP2D6, CYP3A4
		Rank II reaction : FAME 3	CYP1A2, CYP2C19, CYP2C9, CYP2D6, CYP3A4
aromatic_hydroxylation_(sulfur_containing_5ring)	CYPs	Rank I reaction : Way2Drug SOMP	CYP1A2, CYP2C19, CYP2C9, CYP2D6, CYP3A4
		Rank II reaction : FAME 3	CYP1A2, CYP2C19, CYP2C9, CYP2D6, CYP3A4
aromatic_oxidation	CYPs	Rank I reaction : Way2Drug SOMP	CYP1A2, CYP2C19, CYP2C9, CYP2D6, CYP3A4
		Rank II reaction : FAME 3	CYP1A2, CYP2C19, CYP2C9, CYP2D6, CYP3A4
aromatic_oxidation_(nitrogen_containing_5ring)	CYPs	Rank I reaction : Way2Drug SOMP	CYP1A2, CYP2C19, CYP2C9, CYP2D6, CYP3A4
		Rank II reaction : FAME 3	CYP1A2, CYP2C19, CYP2C9, CYP2D6, CYP3A4
azide_cleavage	CYPs	Rank I reaction : Way2Drug SOMP	CYP1A2, CYP2C19, CYP2C9, CYP2D6, CYP3A4
		Rank II reaction : FAME 3	CYP1A2, CYP2C19, CYP2C9, CYP2D6, CYP3A4
benzylic_hydroxylation_(c-CH1-CH3)	CYPs	Rank I reaction : Way2Drug SOMP	CYP1A2, CYP2C19, CYP2C9, CYP2D6, CYP3A4
		Rank II reaction : FAME 3	CYP1A2, CYP2C19, CYP2C9, CYP2D6, CYP3A4
benzylic_hydroxylation_(c-CH1-CR)	CYPs	Rank I reaction : Way2Drug SOMP	CYP1A2, CYP2C19, CYP2C9, CYP2D6, CYP3A4
		Rank II reaction : FAME 3	CYP1A2, CYP2C19, CYP2C9, CYP2D6, CYP3A4
benzylic_hydroxylation_(c-CH2-CH3)	CYPs	Rank I reaction : Way2Drug SOMP	CYP1A2, CYP2C19, CYP2C9, CYP2D6, CYP3A4
		Rank II reaction : FAME 3	CYP1A2, CYP2C19, CYP2C9, CYP2D6, CYP3A4
benzylic_hydroxylation_(c-CH2-CR)	CYPs	Rank I reaction : Way2Drug SOMP	CYP1A2, CYP2C19, CYP2C9, CYP2D6, CYP3A4
		Rank II reaction : FAME 3	CYP1A2, CYP2C19, CYP2C9, CYP2D6, CYP3A4
benzylic_hydroxylation_(c-CH2-N)	CYPs	Rank I reaction : Way2Drug SOMP	CYP1A2, CYP2C19, CYP2C9, CYP2D6, CYP3A4
		Rank II reaction : FAME 3	CYP1A2, CYP2C19, CYP2C9, CYP2D6, CYP3A4
benzylic_hydroxylation_(c-CH3)	CYPs	Rank I reaction : Way2Drug SOMP	CYP1A2, CYP2C19, CYP2C9, CYP2D6, CYP3A4

		Rank II reaction : FAME 3	CYP1A2, CYP2C19, CYP2C9, CYP2D6, CYP3A4
beta-oxidation	CYPs	Rank I reaction : Way2Drug SOMP	CYP1A2, CYP2C19, CYP2C9, CYP2D6, CYP3A4
		Rank II reaction : FAME 3	CYP1A2, CYP2C19, CYP2C9, CYP2D6, CYP3A4
carbonyl_reduction_(aliphatic)	CYPs	Rank I reaction : Way2Drug SOMP	CYP1A2, CYP2C19, CYP2C9, CYP2D6, CYP3A4
		Rank II reaction : FAME 3	CYP1A2, CYP2C19, CYP2C9, CYP2D6, CYP3A4
carbonyl_reduction_(both_sides_next_to_aromatic_carbon)	CYPs	Rank I reaction : Way2Drug SOMP	CYP1A2, CYP2C19, CYP2C9, CYP2D6, CYP3A4
		Rank II reaction : FAME 3	CYP1A2, CYP2C19, CYP2C9, CYP2D6, CYP3A4
carbonyl_reduction_(next_to_SP2_carbon)	CYPs	Rank I reaction : Way2Drug SOMP	CYP1A2, CYP2C19, CYP2C9, CYP2D6, CYP3A4
		Rank II reaction : FAME 3	CYP1A2, CYP2C19, CYP2C9, CYP2D6, CYP3A4
carbonyl_reduction_(next_to_aromatic_carbon)	CYPs	Rank I reaction : Way2Drug SOMP	CYP1A2, CYP2C19, CYP2C9, CYP2D6, CYP3A4
		Rank II reaction : FAME 3	CYP1A2, CYP2C19, CYP2C9, CYP2D6, CYP3A4
carboxylation_(benzylic_CH3)	CYPs	Rank I reaction : Way2Drug SOMP	CYP1A2, CYP2C19, CYP2C9, CYP2D6, CYP3A4
		Rank II reaction : FAME 3	CYP1A2, CYP2C19, CYP2C9, CYP2D6, CYP3A4
carboxylation_(primary_carbon_next_to_SP2)	CYPs	Rank I reaction : Way2Drug SOMP	CYP1A2, CYP2C19, CYP2C9, CYP2D6, CYP3A4
		Rank II reaction : FAME 3	CYP1A2, CYP2C19, CYP2C9, CYP2D6, CYP3A4
carboxylation_(primary_carbon_next_to_quart_carbon)	CYPs	Rank I reaction : Way2Drug SOMP	CYP1A2, CYP2C19, CYP2C9, CYP2D6, CYP3A4
		Rank II reaction : FAME 3	CYP1A2, CYP2C19, CYP2C9, CYP2D6, CYP3A4
carboxylation_(primary_carbon_next_to_sec_carbon)	CYPs	Rank I reaction : Way2Drug SOMP	CYP1A2, CYP2C19, CYP2C9, CYP2D6, CYP3A4
		Rank II reaction : FAME 3	CYP1A2, CYP2C19, CYP2C9, CYP2D6, CYP3A4
carboxylation_(primary_carbon_next_to_tert_carbon)	CYPs	Rank I reaction : Way2Drug SOMP	CYP1A2, CYP2C19, CYP2C9, CYP2D6, CYP3A4
		Rank II reaction : FAME 3	CYP1A2, CYP2C19, CYP2C9, CYP2D6, CYP3A4
cyclic_hemiacetal_ring_opening	CYPs	Rank I reaction : Way2Drug SOMP	CYP1A2, CYP2C19, CYP2C9, CYP2D6, CYP3A4
		Rank II reaction : FAME 3	CYP1A2, CYP2C19, CYP2C9, CYP2D6, CYP3A4
decarboxylation	CYPs	Rank I reaction : Way2Drug SOMP	CYP1A2, CYP2C19, CYP2C9, CYP2D6, CYP3A4
		Rank II reaction : FAME 3	CYP1A2, CYP2C19, CYP2C9, CYP2D6, CYP3A4
dehydration_next_to_SP2_a	CYPs	Rank I reaction : Way2Drug SOMP	CYP1A2, CYP2C19, CYP2C9, CYP2D6, CYP3A4
		Rank II reaction : FAME 3	CYP1A2, CYP2C19, CYP2C9, CYP2D6, CYP3A4
dehydration_next_to_SP2_b	CYPs	Rank I reaction : Way2Drug SOMP	CYP1A2, CYP2C19, CYP2C9, CYP2D6, CYP3A4
		Rank II reaction : FAME 3	CYP1A2, CYP2C19, CYP2C9, CYP2D6, CYP3A4
dehydration_next_to_SP2_both_sides	CYPs	Rank I reaction : Way2Drug SOMP	CYP1A2, CYP2C19, CYP2C9, CYP2D6, CYP3A4
		Rank II reaction : FAME 3	CYP1A2, CYP2C19, CYP2C9, CYP2D6, CYP3A4
dehydrogenation_(CH1-CH3->C=CH2)	CYPs	Rank I reaction : Way2Drug SOMP	CYP1A2, CYP2C19, CYP2C9, CYP2D6, CYP3A4
		Rank II reaction : FAME 3	CYP1A2, CYP2C19, CYP2C9, CYP2D6, CYP3A4
dehydrogenation_(CH2-CH3->C=CH2)	CYPs	Rank I reaction : Way2Drug SOMP	CYP1A2, CYP2C19, CYP2C9, CYP2D6, CYP3A4
		Rank II reaction : FAME 3	CYP1A2, CYP2C19, CYP2C9, CYP2D6, CYP3A4
dehydrogenation_(alpha,beta_to_SP2)	CYPs	Rank I reaction : Way2Drug SOMP	CYP1A2, CYP2C19, CYP2C9, CYP2D6, CYP3A4
		Rank II reaction : FAME 3	CYP1A2, CYP2C19, CYP2C9, CYP2D6, CYP3A4

dehydrogenation_(alpha,beta_to_SP2_both_sides)	CYPs	Rank I reaction : Way2Drug SOMP	CYP1A2, CYP2C19, CYP2C9, CYP2D6, CYP3A4
		Rank II reaction : FAME 3	CYP1A2, CYP2C19, CYP2C9, CYP2D6, CYP3A4
dehydrogenation_(amine)	CYPs	Rank I reaction : Way2Drug SOMP	CYP1A2, CYP2C19, CYP2C9, CYP2D6, CYP3A4
		Rank II reaction : FAME 3	CYP1A2, CYP2C19, CYP2C9, CYP2D6, CYP3A4
dehydrogenation_(aromatization_of_1,4-dihydropyridine)	CYPs	Rank I reaction : Way2Drug SOMP	CYP1A2, CYP2C19, CYP2C9, CYP2D6, CYP3A4
		Rank II reaction : FAME 3	CYP1A2, CYP2C19, CYP2C9, CYP2D6, CYP3A4
deiodonidation	CYPs	Rank I reaction : Way2Drug SOMP	CYP1A2, CYP2C19, CYP2C9, CYP2D6, CYP3A4
		Rank II reaction : FAME 3	CYP1A2, CYP2C19, CYP2C9, CYP2D6, CYP3A4
diazene_cleavage	CYPs	Rank I reaction : Way2Drug SOMP	CYP1A2, CYP2C19, CYP2C9, CYP2D6, CYP3A4
		Rank II reaction : FAME 3	CYP1A2, CYP2C19, CYP2C9, CYP2D6, CYP3A4
double_bond_reduction	CYPs	Rank I reaction : Way2Drug SOMP	CYP1A2, CYP2C19, CYP2C9, CYP2D6, CYP3A4
		Rank II reaction : FAME 3	CYP1A2, CYP2C19, CYP2C9, CYP2D6, CYP3A4
double_bond_reduction_(aromatic)	CYPs	Rank I reaction : Way2Drug SOMP	CYP1A2, CYP2C19, CYP2C9, CYP2D6, CYP3A4
		Rank II reaction : FAME 3	CYP1A2, CYP2C19, CYP2C9, CYP2D6, CYP3A4
double_bond_reduction_(benzylic)	CYPs	Rank I reaction : Way2Drug SOMP	CYP1A2, CYP2C19, CYP2C9, CYP2D6, CYP3A4
		Rank II reaction : FAME 3	CYP1A2, CYP2C19, CYP2C9, CYP2D6, CYP3A4
epoxidation	CYPs	Rank I reaction : Way2Drug SOMP	CYP1A2, CYP2C19, CYP2C9, CYP2D6, CYP3A4
		Rank II reaction : FAME 3	CYP1A2, CYP2C19, CYP2C9, CYP2D6, CYP3A4
epoxide_hydrolysis	CYPs	Rank I reaction : Way2Drug SOMP	CYP1A2, CYP2C19, CYP2C9, CYP2D6, CYP3A4
		Rank II reaction : FAME 3	CYP1A2, CYP2C19, CYP2C9, CYP2D6, CYP3A4
haloacid_hydrolysis	CYPs	Rank I reaction : Way2Drug SOMP	CYP1A2, CYP2C19, CYP2C9, CYP2D6, CYP3A4
		Rank II reaction : FAME 3	CYP1A2, CYP2C19, CYP2C9, CYP2D6, CYP3A4
het-O-demethylation	CYPs	Rank I reaction : Way2Drug SOMP	CYP1A2, CYP2C19, CYP2C9, CYP2D6, CYP3A4
		Rank II reaction : FAME 3	CYP1A2, CYP2C19, CYP2C9, CYP2D6, CYP3A4
hydrazone_hydrolysis	CYPs	Rank I reaction : Way2Drug SOMP	CYP1A2, CYP2C19, CYP2C9, CYP2D6, CYP3A4
		Rank II reaction : FAME 3	CYP1A2, CYP2C19, CYP2C9, CYP2D6, CYP3A4
hydrolysis_(CNC(OH)R)	CYPs	Rank I reaction : Way2Drug SOMP	CYP1A2, CYP2C19, CYP2C9, CYP2D6, CYP3A4
		Rank II reaction : FAME 3	CYP1A2, CYP2C19, CYP2C9, CYP2D6, CYP3A4
hydrolysis_(N-substituted-pyridine)	CYPs	Rank I reaction : Way2Drug SOMP	CYP1A2, CYP2C19, CYP2C9, CYP2D6, CYP3A4
		Rank II reaction : FAME 3	CYP1A2, CYP2C19, CYP2C9, CYP2D6, CYP3A4
hydrolysis_(X=X-X_exclude_phosphate)	CYPs	Rank I reaction : Way2Drug SOMP	CYP1A2, CYP2C19, CYP2C9, CYP2D6, CYP3A4
		Rank II reaction : FAME 3	CYP1A2, CYP2C19, CYP2C9, CYP2D6, CYP3A4
hydrolysis_(ester)	CYPs	Rank I reaction : Way2Drug SOMP	CYP1A2, CYP2C19, CYP2C9, CYP2D6, CYP3A4
		Rank II reaction : FAME 3	CYP1A2, CYP2C19, CYP2C9, CYP2D6, CYP3A4
hydrolysis_(heteroatom_bonded_amide)	CYPs	Rank I reaction : Way2Drug SOMP	CYP1A2, CYP2C19, CYP2C9, CYP2D6, CYP3A4
		Rank II reaction : FAME 3	CYP1A2, CYP2C19, CYP2C9, CYP2D6, CYP3A4
hydrolysis_(methoxyester)	CYPs	Rank I reaction : Way2Drug SOMP	CYP1A2, CYP2C19, CYP2C9, CYP2D6, CYP3A4

		Rank II reaction : FAME 3	CYP1A2, CYP2C19, CYP2C9, CYP2D6, CYP3A4
hydrolysis_(primary_amide)	CYPs	Rank I reaction : Way2Drug SOMP	CYP1A2, CYP2C19, CYP2C9, CYP2D6, CYP3A4
		Rank II reaction : FAME 3	CYP1A2, CYP2C19, CYP2C9, CYP2D6, CYP3A4
hydrolysis_(secondary_amide)	CYPs	Rank I reaction : Way2Drug SOMP	CYP1A2, CYP2C19, CYP2C9, CYP2D6, CYP3A4
		Rank II reaction : FAME 3	CYP1A2, CYP2C19, CYP2C9, CYP2D6, CYP3A4
hydrolysis_(tertiary_amide)	CYPs	Rank I reaction : Way2Drug SOMP	CYP1A2, CYP2C19, CYP2C9, CYP2D6, CYP3A4
		Rank II reaction : FAME 3	CYP1A2, CYP2C19, CYP2C9, CYP2D6, CYP3A4
hydrolysis_(urea_or_carbonate)	CYPs	Rank I reaction : Way2Drug SOMP	CYP1A2, CYP2C19, CYP2C9, CYP2D6, CYP3A4
		Rank II reaction : FAME 3	CYP1A2, CYP2C19, CYP2C9, CYP2D6, CYP3A4
hydroxyl-amide_5ring_closure	CYPs	Rank I reaction : Way2Drug SOMP	CYP1A2, CYP2C19, CYP2C9, CYP2D6, CYP3A4
		Rank II reaction : FAME 3	CYP1A2, CYP2C19, CYP2C9, CYP2D6, CYP3A4
hydroxyl-amide_5ring_rearr	CYPs	Rank I reaction : Way2Drug SOMP	CYP1A2, CYP2C19, CYP2C9, CYP2D6, CYP3A4
		Rank II reaction : FAME 3	CYP1A2, CYP2C19, CYP2C9, CYP2D6, CYP3A4
hydroxyl-amide_6ring_rearr	CYPs	Rank I reaction : Way2Drug SOMP	CYP1A2, CYP2C19, CYP2C9, CYP2D6, CYP3A4
		Rank II reaction : FAME 3	CYP1A2, CYP2C19, CYP2C9, CYP2D6, CYP3A4
imine_hydrolysis	CYPs	Rank I reaction : Way2Drug SOMP	CYP1A2, CYP2C19, CYP2C9, CYP2D6, CYP3A4
		Rank II reaction : FAME 3	CYP1A2, CYP2C19, CYP2C9, CYP2D6, CYP3A4
isopropenyl_oxidation	CYPs	Rank I reaction : Way2Drug SOMP	CYP1A2, CYP2C19, CYP2C9, CYP2D6, CYP3A4
		Rank II reaction : FAME 3	CYP1A2, CYP2C19, CYP2C9, CYP2D6, CYP3A4
n-deglycosidation	CYPs	Rank I reaction : Way2Drug SOMP	CYP1A2, CYP2C19, CYP2C9, CYP2D6, CYP3A4
		Rank II reaction : FAME 3	CYP1A2, CYP2C19, CYP2C9, CYP2D6, CYP3A4
nitrile_to_amide	CYPs	Rank I reaction : Way2Drug SOMP	CYP1A2, CYP2C19, CYP2C9, CYP2D6, CYP3A4
		Rank II reaction : FAME 3	CYP1A2, CYP2C19, CYP2C9, CYP2D6, CYP3A4
nitro_to_aniline	CYPs	Rank I reaction : Way2Drug SOMP	CYP1A2, CYP2C19, CYP2C9, CYP2D6, CYP3A4
		Rank II reaction : FAME 3	CYP1A2, CYP2C19, CYP2C9, CYP2D6, CYP3A4
nitro_to_nitroso	CYPs	Rank I reaction : Way2Drug SOMP	CYP1A2, CYP2C19, CYP2C9, CYP2D6, CYP3A4
		Rank II reaction : FAME 3	CYP1A2, CYP2C19, CYP2C9, CYP2D6, CYP3A4
oxidation_(C=N)	CYPs	Rank I reaction : Way2Drug SOMP	CYP1A2, CYP2C19, CYP2C9, CYP2D6, CYP3A4
		Rank II reaction : FAME 3	CYP1A2, CYP2C19, CYP2C9, CYP2D6, CYP3A4
oxidation_(amine_in_a_ring)	CYPs	Rank I reaction : Way2Drug SOMP	CYP1A2, CYP2C19, CYP2C9, CYP2D6, CYP3A4
		Rank II reaction : FAME 3	CYP1A2, CYP2C19, CYP2C9, CYP2D6, CYP3A4
oxidation_to_quinone	CYPs	Rank I reaction : Way2Drug SOMP	CYP1A2, CYP2C19, CYP2C9, CYP2D6, CYP3A4
		Rank II reaction : FAME 3	CYP1A2, CYP2C19, CYP2C9, CYP2D6, CYP3A4
oxidative_deamination_(amidine)	CYPs	Rank I reaction : Way2Drug SOMP	CYP1A2, CYP2C19, CYP2C9, CYP2D6, CYP3A4
		Rank II reaction : FAME 3	CYP1A2, CYP2C19, CYP2C9, CYP2D6, CYP3A4
oxidative_deamination_(aromatic)	CYPs	Rank I reaction : Way2Drug SOMP	CYP1A2, CYP2C19, CYP2C9, CYP2D6, CYP3A4
		Rank II reaction : FAME 3	CYP1A2, CYP2C19, CYP2C9, CYP2D6, CYP3A4

oxidative_deamination_(on_primary_carbon)	CYPs	Rank I reaction : Way2Drug SOMP	CYP1A2, CYP2C19, CYP2C9, CYP2D6, CYP3A4
		Rank II reaction : FAME 3	CYP1A2, CYP2C19, CYP2C9, CYP2D6, CYP3A4
oxidative_deamination_(on_secondary_carbon)	CYPs	Rank I reaction : Way2Drug SOMP	CYP1A2, CYP2C19, CYP2C9, CYP2D6, CYP3A4
		Rank II reaction : FAME 3	CYP1A2, CYP2C19, CYP2C9, CYP2D6, CYP3A4
oxidative_decarboxylation	CYPs	Rank I reaction : Way2Drug SOMP	CYP1A2, CYP2C19, CYP2C9, CYP2D6, CYP3A4
		Rank II reaction : FAME 3	CYP1A2, CYP2C19, CYP2C9, CYP2D6, CYP3A4
oxidative_dehalogenation	CYPs	Rank I reaction : Way2Drug SOMP	CYP1A2, CYP2C19, CYP2C9, CYP2D6, CYP3A4
		Rank II reaction : FAME 3	CYP1A2, CYP2C19, CYP2C9, CYP2D6, CYP3A4
phosphine_sulphide_hydrolysis	CYPs	Rank I reaction : Way2Drug SOMP	CYP1A2, CYP2C19, CYP2C9, CYP2D6, CYP3A4
		Rank II reaction : FAME 3	CYP1A2, CYP2C19, CYP2C9, CYP2D6, CYP3A4
primary_alcohol_oxidation_(aliphatic)	CYPs	Rank I reaction : Way2Drug SOMP	CYP1A2, CYP2C19, CYP2C9, CYP2D6, CYP3A4
		Rank II reaction : FAME 3	CYP1A2, CYP2C19, CYP2C9, CYP2D6, CYP3A4
primary_alcohol_oxidation_(benzylic)	CYPs	Rank I reaction : Way2Drug SOMP	CYP1A2, CYP2C19, CYP2C9, CYP2D6, CYP3A4
		Rank II reaction : FAME 3	CYP1A2, CYP2C19, CYP2C9, CYP2D6, CYP3A4
ring_closure_(NH1-5bonds-carboxyl)2	CYPs	Rank I reaction : Way2Drug SOMP	CYP1A2, CYP2C19, CYP2C9, CYP2D6, CYP3A4
		Rank II reaction : FAME 3	CYP1A2, CYP2C19, CYP2C9, CYP2D6, CYP3A4
ring_closure_(NH1-6bonds-carboxyl)	CYPs	Rank I reaction : Way2Drug SOMP	CYP1A2, CYP2C19, CYP2C9, CYP2D6, CYP3A4
		Rank II reaction : FAME 3	CYP1A2, CYP2C19, CYP2C9, CYP2D6, CYP3A4
ring_closure_(hydroxyl-5bonds-carboxyl)	CYPs	Rank I reaction : Way2Drug SOMP	CYP1A2, CYP2C19, CYP2C9, CYP2D6, CYP3A4
		Rank II reaction : FAME 3	CYP1A2, CYP2C19, CYP2C9, CYP2D6, CYP3A4
ring_closure_(hydroxyl-6bonds-carboxyl)	CYPs	Rank I reaction : Way2Drug SOMP	CYP1A2, CYP2C19, CYP2C9, CYP2D6, CYP3A4
		Rank II reaction : FAME 3	CYP1A2, CYP2C19, CYP2C9, CYP2D6, CYP3A4
secondary_N-depropylation	CYPs	Rank I reaction : Way2Drug SOMP	CYP1A2, CYP2C19, CYP2C9, CYP2D6, CYP3A4
		Rank II reaction : FAME 3	CYP1A2, CYP2C19, CYP2C9, CYP2D6, CYP3A4
secondary_alcohol_oxidation_(aliphatic)	CYPs	Rank I reaction : Way2Drug SOMP	CYP1A2, CYP2C19, CYP2C9, CYP2D6, CYP3A4
		Rank II reaction : FAME 3	CYP1A2, CYP2C19, CYP2C9, CYP2D6, CYP3A4
secondary_alcohol_oxidation_(benzylic)	CYPs	Rank I reaction : Way2Drug SOMP	CYP1A2, CYP2C19, CYP2C9, CYP2D6, CYP3A4
		Rank II reaction : FAME 3	CYP1A2, CYP2C19, CYP2C9, CYP2D6, CYP3A4
secondary_aliphatic_carbon_hydroxylation	CYPs	Rank I reaction : Way2Drug SOMP	CYP1A2, CYP2C19, CYP2C9, CYP2D6, CYP3A4
		Rank II reaction : FAME 3	CYP1A2, CYP2C19, CYP2C9, CYP2D6, CYP3A4
steroid_17hydroxy_to_keto	CYPs	Rank I reaction : Way2Drug SOMP	CYP1A2, CYP2C19, CYP2C9, CYP2D6, CYP3A4
		Rank II reaction : FAME 3	CYP1A2, CYP2C19, CYP2C9, CYP2D6, CYP3A4
steroid_d5d4	CYPs	Rank I reaction : Way2Drug SOMP	CYP1A2, CYP2C19, CYP2C9, CYP2D6, CYP3A4
		Rank II reaction : FAME 3	CYP1A2, CYP2C19, CYP2C9, CYP2D6, CYP3A4
sulfide_oxidation_(C-S-C)	CYPs	Rank I reaction : Way2Drug SOMP	CYP1A2, CYP2C19, CYP2C9, CYP2D6, CYP3A4
		Rank II reaction : FAME 3	CYP1A2, CYP2C19, CYP2C9, CYP2D6, CYP3A4
sulfide_oxidation_(c-S-C)	CYPs	Rank I reaction : Way2Drug SOMP	CYP1A2, CYP2C19, CYP2C9, CYP2D6, CYP3A4

		Rank II reaction : FAME 3	CYP1A2, CYP2C19, CYP2C9, CYP2D6, CYP3A4
sulfide_oxidation_(c-S-c)	CYPs	Rank I reaction : Way2Drug SOMP	CYP1A2, CYP2C19, CYP2C9, CYP2D6, CYP3A4
		Rank II reaction : FAME 3	CYP1A2, CYP2C19, CYP2C9, CYP2D6, CYP3A4
sulfoxide_oxidation_(C-S-C)	CYPs	Rank I reaction : Way2Drug SOMP	CYP1A2, CYP2C19, CYP2C9, CYP2D6, CYP3A4
		Rank II reaction : FAME 3	CYP1A2, CYP2C19, CYP2C9, CYP2D6, CYP3A4
sulfoxide_oxidation_(c-S-C)	CYPs	Rank I reaction : Way2Drug SOMP	CYP1A2, CYP2C19, CYP2C9, CYP2D6, CYP3A4
		Rank II reaction : FAME 3	CYP1A2, CYP2C19, CYP2C9, CYP2D6, CYP3A4
sulfoxide_oxidation_(c-S-c)	CYPs	Rank I reaction : Way2Drug SOMP	CYP1A2, CYP2C19, CYP2C9, CYP2D6, CYP3A4
		Rank II reaction : FAME 3	CYP1A2, CYP2C19, CYP2C9, CYP2D6, CYP3A4
sulfoxide_reduction	CYPs	Rank I reaction : Way2Drug SOMP	CYP1A2, CYP2C19, CYP2C9, CYP2D6, CYP3A4
		Rank II reaction : FAME 3	CYP1A2, CYP2C19, CYP2C9, CYP2D6, CYP3A4
tautomerisation_(keto->enol)	CYPs	Rank I reaction : Way2Drug SOMP	CYP1A2, CYP2C19, CYP2C9, CYP2D6, CYP3A4
		Rank II reaction : FAME 3	CYP1A2, CYP2C19, CYP2C9, CYP2D6, CYP3A4
tertiary_N-depropylation	CYPs	Rank I reaction : Way2Drug SOMP	CYP1A2, CYP2C19, CYP2C9, CYP2D6, CYP3A4
		Rank II reaction : FAME 3	CYP1A2, CYP2C19, CYP2C9, CYP2D6, CYP3A4
thiophene_oxidation	CYPs	Rank I reaction : Way2Drug SOMP	CYP1A2, CYP2C19, CYP2C9, CYP2D6, CYP3A4
		Rank II reaction : FAME 3	CYP1A2, CYP2C19, CYP2C9, CYP2D6, CYP3A4
try	CYPs	Rank I reaction : Way2Drug SOMP	CYP1A2, CYP2C19, CYP2C9, CYP2D6, CYP3A4
		Rank II reaction : FAME 3	CYP1A2, CYP2C19, CYP2C9, CYP2D6, CYP3A4
vinyl_oxidation	CYPs	Rank I reaction : Way2Drug SOMP	CYP1A2, CYP2C19, CYP2C9, CYP2D6, CYP3A4
		Rank II reaction : FAME 3	CYP1A2, CYP2C19, CYP2C9, CYP2D6, CYP3A4
xanthine_oxidation	CYPs	Rank I reaction : Way2Drug SOMP	CYP1A2, CYP2C19, CYP2C9, CYP2D6, CYP3A4
		Rank II reaction : FAME 3	CYP1A2, CYP2C19, CYP2C9, CYP2D6, CYP3A4
N-acetylation_(NH1)	NATs	Rank I reaction : No Tool	No Enzyme Annotated
		Rank II reaction : FAME 3	NATs
N-acetylation_(NH1-CH3)	NATs	Rank I reaction : No Tool	No Enzyme Annotated
		Rank II reaction : FAME 3	NATs
N-acetylation_(aniline)	NATs	Rank I reaction : No Tool	No Enzyme Annotated
		Rank II reaction : FAME 3	NATs
N-acetylation_(aromatic_-nH-)	NATs	Rank I reaction : No Tool	No Enzyme Annotated
		Rank II reaction : FAME 3	NATs
N-acetylation_(heteroatom_bonded_NH2)	NATs	Rank I reaction : No Tool	No Enzyme Annotated
		Rank II reaction : FAME 3	NATs
N-glucuronidation_(N(CH3)2)	UGTs	Rank I reaction : Way2Drug SOMP	UGTs
		Rank II reaction : FAME 3	UGTs
N-glucuronidation_(NCH3_in_a_ring)	UGTs	Rank I reaction : Way2Drug SOMP	UGTs
		Rank II reaction : FAME 3	UGTs

N-glucuronidation_(NH_in_a_ring)	UGTs	Rank I reaction : Way2Drug SOMP	UGTs
		Rank II reaction : FAME 3	UGTs
N-glucuronidation_(aliphatic_NH2)	UGTs	Rank I reaction : Way2Drug SOMP	UGTs
		Rank II reaction : FAME 3	UGTs
N-glucuronidation_(aniline)	UGTs	Rank I reaction : Way2Drug SOMP	UGTs
		Rank II reaction : FAME 3	UGTs
N-glucuronidation_(aniline_NH1-R)	UGTs	Rank I reaction : Way2Drug SOMP	UGTs
		Rank II reaction : FAME 3	UGTs
N-glucuronidation_(aromatic_-nH-)	UGTs	Rank I reaction : Way2Drug SOMP	UGTs
		Rank II reaction : FAME 3	UGTs
N-glucuronidation_(aromatic_=n-)	UGTs	Rank I reaction : Way2Drug SOMP	UGTs
		Rank II reaction : FAME 3	UGTs
O-glucuronidation_(N-hydroxyl)	UGTs	Rank I reaction : Way2Drug SOMP	UGTs
		Rank II reaction : FAME 3	UGTs
O-glucuronidation_(aliphatic_carboxyl)	UGTs	Rank I reaction : Way2Drug SOMP	UGTs
		Rank II reaction : FAME 3	UGTs
O-glucuronidation_(aliphatic_hydroxyl)	UGTs	Rank I reaction : Way2Drug SOMP	UGTs
		Rank II reaction : FAME 3	UGTs
O-glucuronidation_(aromatic_carboxyl)	UGTs	Rank I reaction : Way2Drug SOMP	UGTs
		Rank II reaction : FAME 3	UGTs
O-glucuronidation_(aromatic_hydroxyl)	UGTs	Rank I reaction : Way2Drug SOMP	UGTs
		Rank II reaction : FAME 3	UGTs
aliphatic_N_sulfation1	SULTs	Rank I reaction : No Tool	No Enzyme Annotated
		Rank II reaction : FAME 3	SULTs
aliphatic_N_sulfation2	SULTs	Rank I reaction : No Tool	No Enzyme Annotated
		Rank II reaction : FAME 3	SULTs
aromatic_N_sulfation2	SULTs	Rank I reaction : No Tool	No Enzyme Annotated
		Rank II reaction : FAME 3	SULTs
sulfation_(aliphatic_hydroxyl)	SULTs	Rank I reaction : No Tool	No Enzyme Annotated
		Rank II reaction : FAME 3	SULTs
sulfation_(aniline)	SULTs	Rank I reaction : No Tool	No Enzyme Annotated
		Rank II reaction : FAME 3	SULTs
sulfation_(aromatic_hydroxyl)	SULTs	Rank I reaction : No Tool	No Enzyme Annotated
		Rank II reaction : FAME 3	SULTs
Glutathionation(+SX)	GSTs	Rank I reaction : No Tool	No Enzyme Annotated
		Rank II reaction : FAME 3	GSTs
