# Supplementary material for: Constructing xenobiotic maps of metabolism to predict enzymes catalyzing metabolites capable of binding to DNA
Source: BMC Bioinformatics. 2021 Sep 21;22:450. doi: 10.1186/s12859-021-04363-6 (PMC8454073; doi:10.1186/s12859-021-04363-6)
Supplement: Supplementary file 3 — Additional file 3.: Extended Figure 4 The file provides the figure 4 in a plain page to make structures and text more readable. [file 12859_2021_4363_MOESM3_ESM.pdf]

Constructing xenobiotic maps of metabolism to predict enzymes catalyzing metabolites capable of binding to DNA.

Conan M., Théret N., Langouet S. and Siegel, A

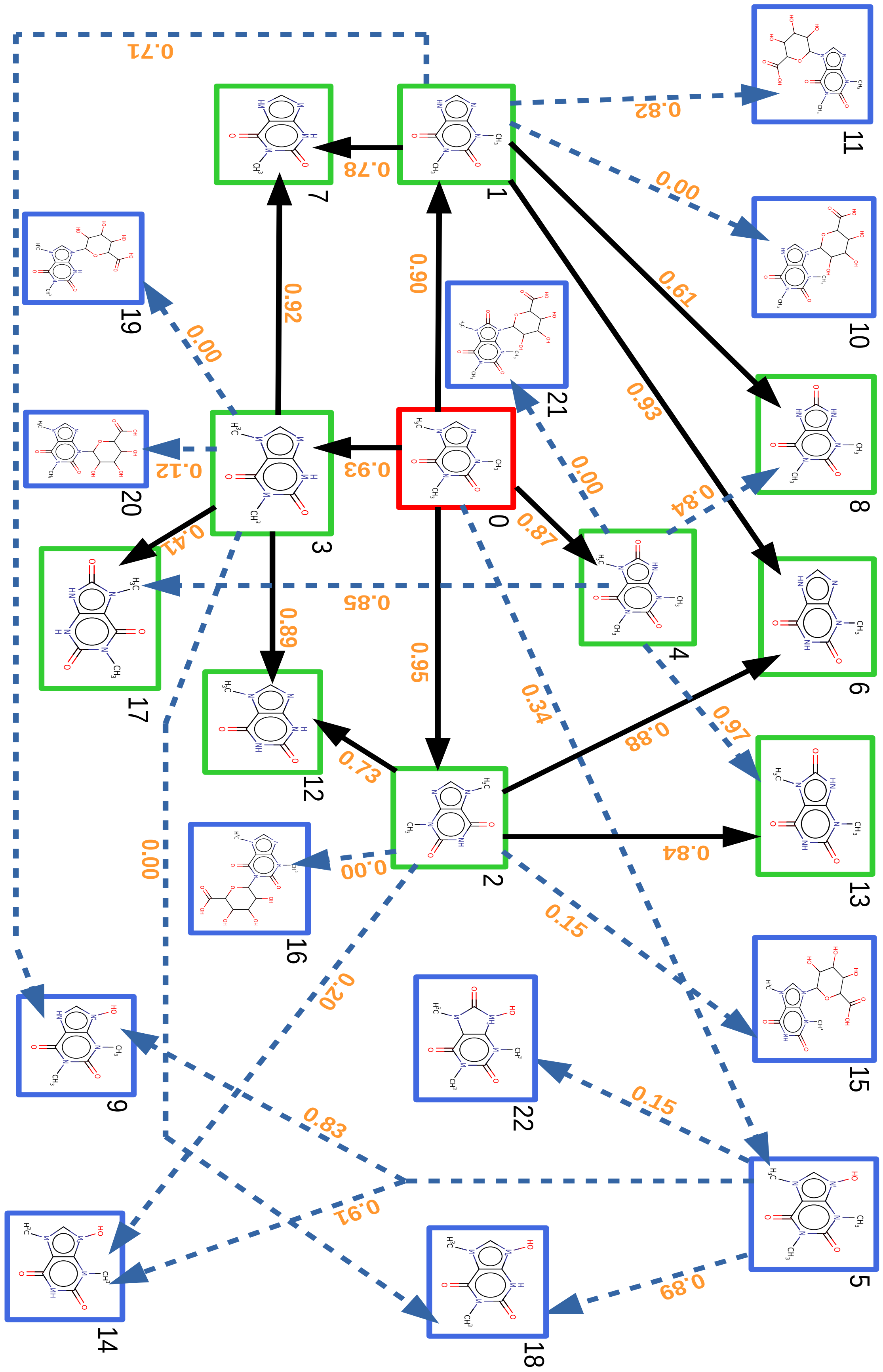

Enlarged version of Figure 4 Enlarged version of figure 4. Caffeine node is shown in red and metabolites which have been experimentally observed are shown in green. Reactions depicted by black arrows are transformations with literature-based evidence. Site of metabolism (SOM) score annotating reactions are shown in orange bolt on arrows.
