# Supplementary material for: Constructing xenobiotic maps of metabolism to predict enzymes catalyzing metabolites capable of binding to DNA
Source: BMC Bioinformatics. 2021 Sep 21;22:450. doi: 10.1186/s12859-021-04363-6 (PMC8454073; doi:10.1186/s12859-021-04363-6)
Supplement: Supplementary file 4 — Additional file 4.: Extended Figure 5 The file provides the figure 5 in a plain page to make structures and text more readable. [file 12859_2021_4363_MOESM4_ESM.pdf]

Constructing xenobiotic maps of metabolism to predict enzymes catalyzing metabolites capable of binding to DNA.

Conan M., Théret N., Langouet S. and Siegel, A

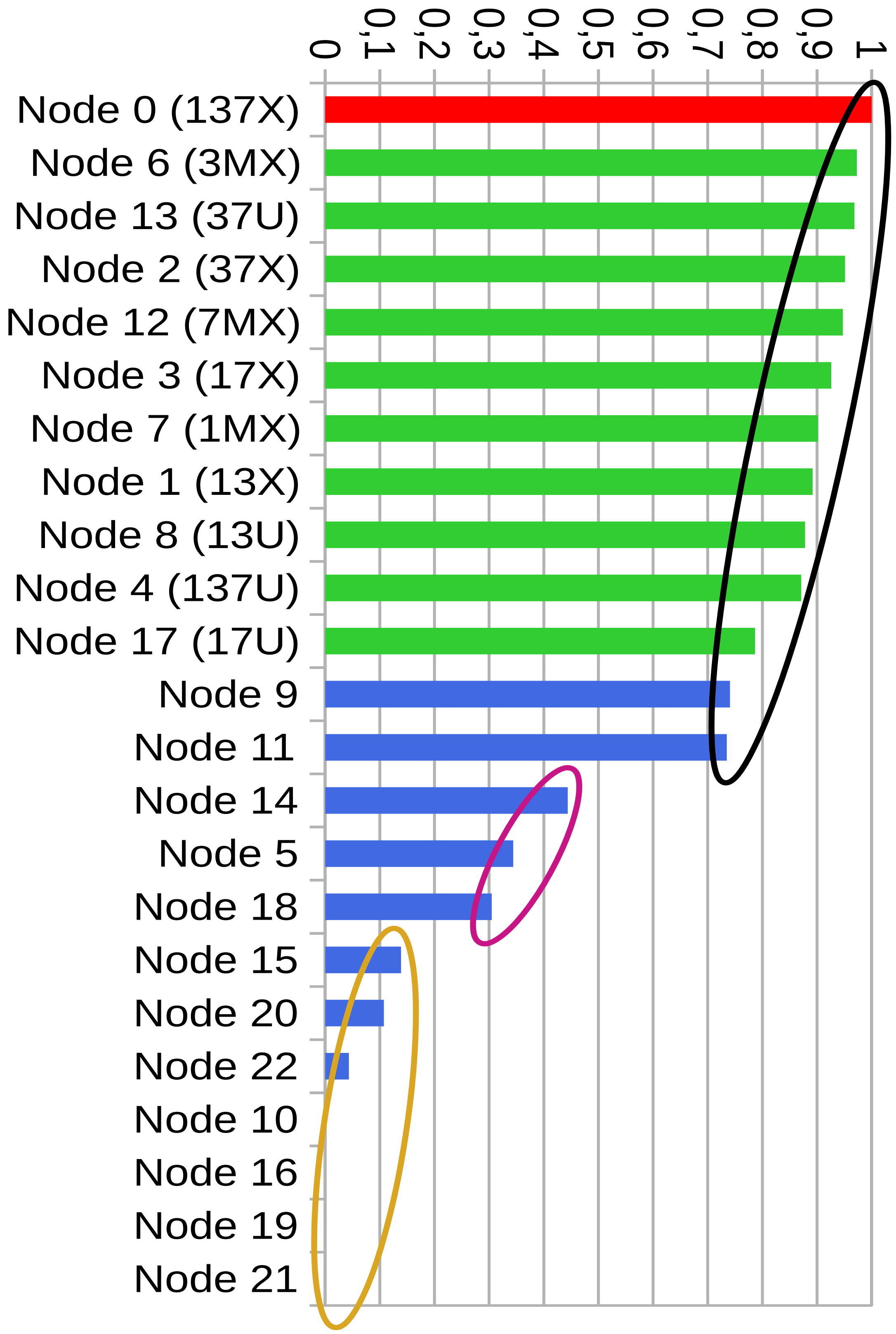

Enlarged version of Figure 5 (a) Distribution of production probability score of each metabolite. Red, green and blue bars are associated with caffeine, known and unknown metabolites, respectively. Metabolites are grouped into three groups according to their range of scores (black, red and yellow ellipses).

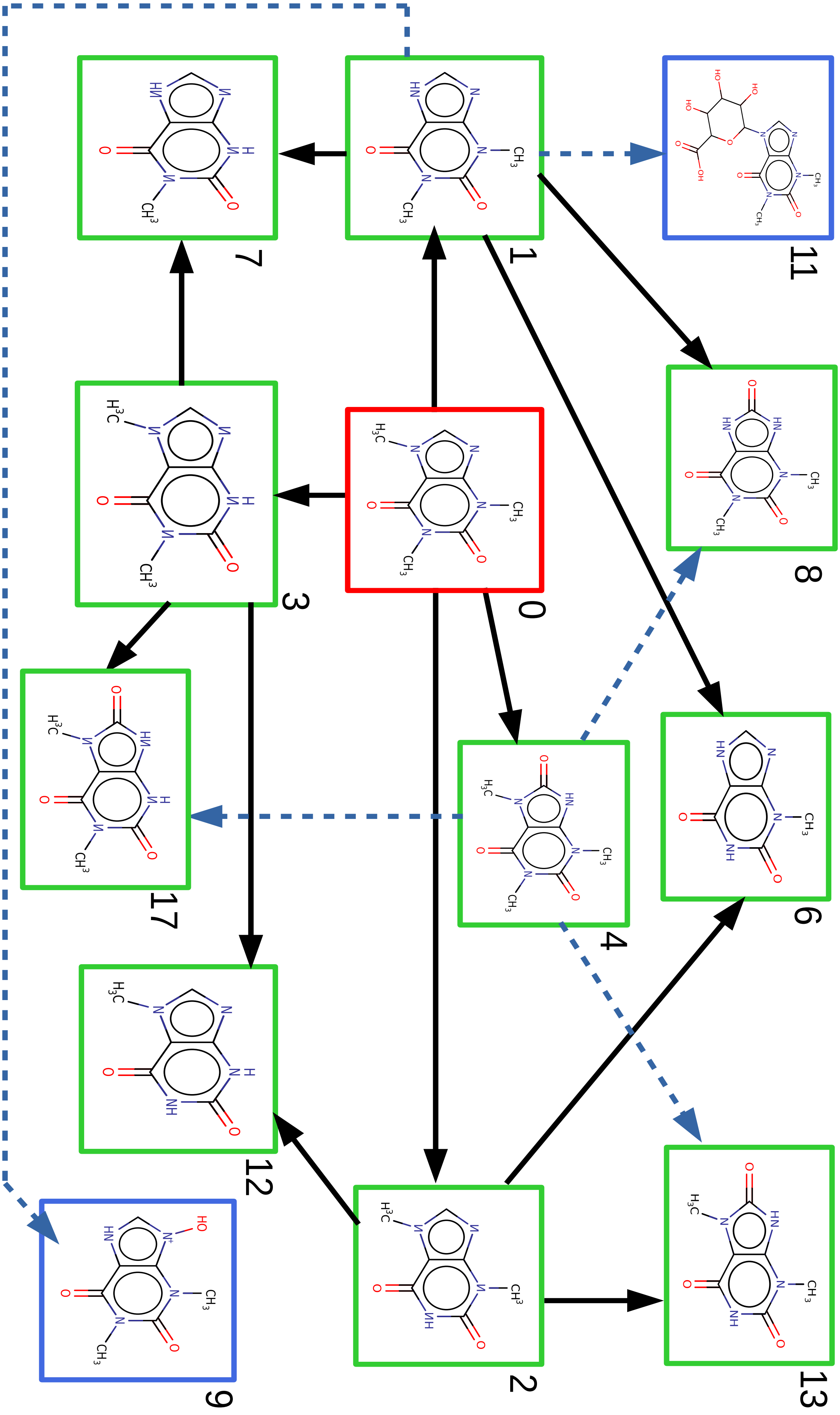

Enlarged version of Figure 5 (b) This map is the filtration of the predicted caffeine metabolism map. The map contains 13 metabolites and 17 reactions. Two unknown metabolites (nodes 9 and 11) are predicted to be nearly as likely to be produced as the known metabolites according to the production probability scores.
