# Supplementary material for: Constructing xenobiotic maps of metabolism to predict enzymes catalyzing metabolites capable of binding to DNA
Source: BMC Bioinformatics. 2021 Sep 21;22:450. doi: 10.1186/s12859-021-04363-6 (PMC8454073; doi:10.1186/s12859-021-04363-6)
Supplement: Supplementary file 5 — Additional file 5.: Description of thirty HAA and Caffeine structure used as pipeline input This pdf file describes the SMILES formula and 2D-structure of each of the thirty HAA and the Caffeine studied in the paper. [file 12859_2021_4363_MOESM5_ESM.pdf]

Constructing xenobiotic maps of metabolism to predict enzymes catalyzing metabolites capable of binding to DNA.

Conan M., Théret N., Langouet S. and Siegel, A

Additional file 5. **Description of thirty HAAs and Caffeine structure used as pipeline input**. This pdf file describes the SMILES formula and 2D-structure of each of the thirty HAAs and the Caffeine studied.

Xenobiotic Name	SMILES formula	2D Structure
1,5,6-TMIP	N=1C=2C=C(C(=CC2N(C1N)C)C)C	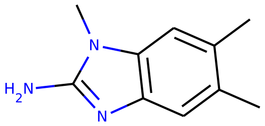
1,6-DMIP	N=1C=2C=CC(=CC2N(C1N)C)C	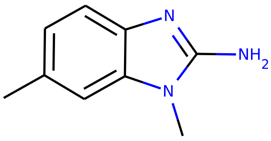
3,5,6-TMIP	N=1C=2C=C(C(=NC2N(C1N)C)C)C	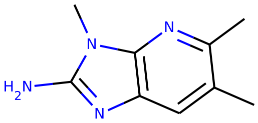
4-CH$_2$OH-8-MeIQx	OCC1=CC=2N=CC(=NC2C=3N=C(N)N(C31)C)C	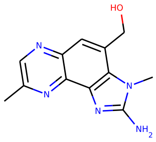
4,7,8-TriMeIQx	N=1C=2C=C(C3=C(N=C(N)N3C)C2N=C(C1C)C)C	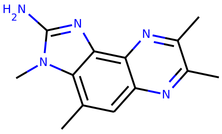
4,8-DiMeIQx	N1=CC(=NC2=C1C=C(C3=C2N=C(N)N3C)C)C	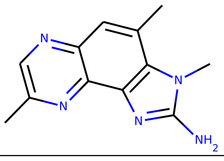
4'-OH-PhIP	OC=1C=CC(=CC1)C=2C=NC=3N=C(N)N(C3C2)C	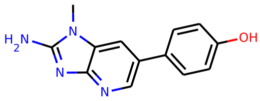
6,7-DiMeIgQx	N=1C=2C=C3N=C(C(=NC3=CC2N(C1N)C)C)C	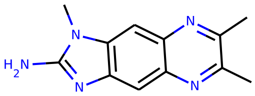

7-MeIgQx	N1=CC(=NC2=CC3=C(N=C(N)N3C)C=C12)C	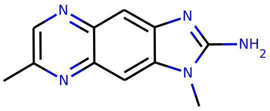
7,8-DiMeIQx	N=1C=2C=CC3=C(N=C(N)N3C)C2N=C(C1C)C	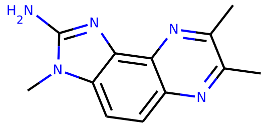
7,9-DiMeIgQx	N1=CC(=NC=2C1=CC=3N=C(N)N(C3C2C)C)C	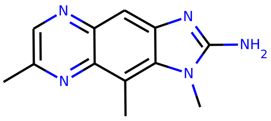
AαC	N=1C(N)=CC=C2C1NC=3C=CC=CC32	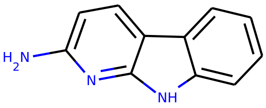
AMPNH	N=1C=CC=2C=3C=CC=CC3N(C4=CC=C(N)C(=C4)C)C2C1	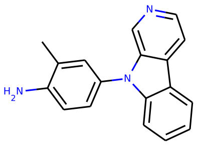
APNH	N=1C=CC=2C=3C=CC=CC3N(C4=CC=C(N)C=C4)C2C1	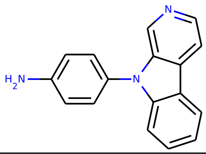
GluP1	N1=C(N)C=CC=2N=C3C(=CC=CN3C12)C	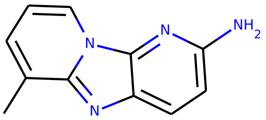
GluP2	N1=C(N)C=CC=2N=C3C=CC=CN3C12	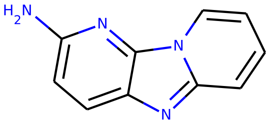
Harman	N=1C=CC=2C=3C=CC=CC3NC2C1C	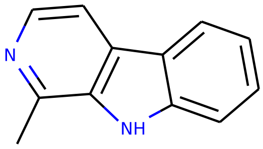
IFP	N1=C2N=C(N)N(C2=CC=3OC(=CC13)C)C	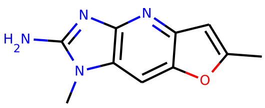

IgQx	N1=CC=NC2=CC3=C(N=C(N)N3C)C=C12	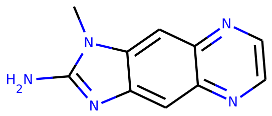
IQ	N1=CC=CC2=C1C=CC3=C2N=C(N)N3C	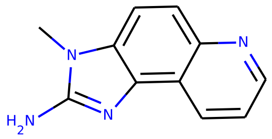
IQ[4,5-b]	N1=C2N=C(N)N(C2=CC3=CC=CC=C13)C	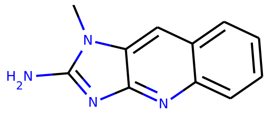
IQx	N1=CC=NC2=C1C=CC3=C2N=C(N)N3C	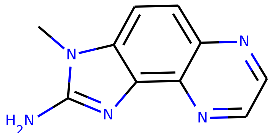
MeAαC	N=1C(N)=C(C=C2C1NC=3C=CC=CC32)C	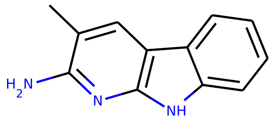
MeIQ	N1=CC=CC2=C1C=C(C3=C2N=C(N)N3C)C	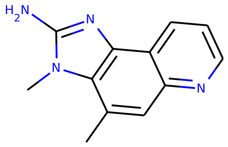
MeIQx	N1=CC(=NC2=C1C=CC3=C2N=C(N)N3C)C	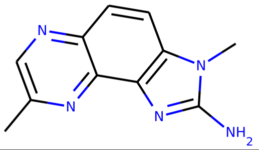
Norharman	N=1C=CC2=C(C1)NC=3C=CC=CC32	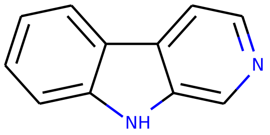
PheP1	N1=CC(=CC=C1N)C2=CC=CC=C2	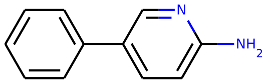
PhIP	N1=CC(=CC2=C1N=C(N)N2C)C=3C=CC=CC3	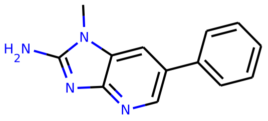

TrP1	N=1C(N)=C(C=2NC=3C=CC=CC3C2C1C)C	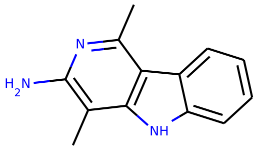
TrP2	N1=C(N)C=C2NC=3C=CC=CC3C2=C1C	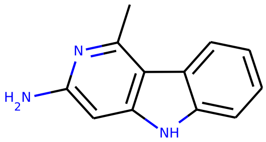
Caffeine	CN1C=NC2=C1C(=O)N(C(=O)N2C)C	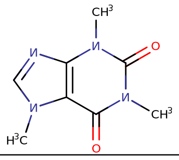
