# Supplementary material for: Constructing xenobiotic maps of metabolism to predict enzymes catalyzing metabolites capable of binding to DNA
Source: BMC Bioinformatics. 2021 Sep 21;22:450. doi: 10.1186/s12859-021-04363-6 (PMC8454073; doi:10.1186/s12859-021-04363-6)
Supplement: Supplementary file 6 — Additional file 6.: Metabolites of AαC, PhIP and MeIQx founded in predicted metabolism maps The file provides a table describing for each metabolite experimentally described if they are predicted by SyGMa and if they are known to produce DNA adduct. [file 12859_2021_4363_MOESM6_ESM.pdf]

Constructing xenobiotic maps of metabolism to predict enzymes catalyzing metabolites capable of binding to DNA

Conan M. , Théret N., Langouet S. and Siegel, A

Additional file 6. **Description of six HAA maps of metabolism**

The file provides a detailed description of metabolites for the six HAAs filtered maps of metabolism built in the paper. For each metabolite of each map, the file provides the identifier of the metabolite, its SMILES formula, its production probability score, its reactivity to DNA and the score of XenoSite Reactivity.

4,7,8-TriMeIQx

Metabolites_ID	SMILES_Formula	Production probability score	Reactive_to_DNA (>=0.85)	XenoSite Reactivity score
0	Cc1nc2cc(C)c3c(nc(N)n3C)c2nc1C	1.0000	True	0.9519193669740386
1	Cc1nc2cc(C)c3[nH]c(N)nc3c2nc1C	0.8170	True	0.952519771988244
3	Cc1nc2cc(C(=O)O)c3c(nc(N)n3C)c2nc1C	0.3929	True	0.9364105737792748
4	Cc1nc2cc(C)c3c(nc(N)n3C)c2nc1C(=O)O	0.3578	True	0.936508784540186
5	Cc1nc2c(cc(C)c3c2nc(N)n3C)nc1C(=O)O	0.3727	True	0.936508784540186
6	Cc1nc2cc(CO)c3c(nc(N)n3C)c2nc1C	0.6010	True	0.9449386351114564
7	Cc1nc2cc(C)c3c(nc(N)n3C)c2nc1CO	0.5520	True	0.9450602682223948
8	Cc1nc2c(cc(C)c3c2nc(N)n3C)nc1CO	0.5520	True	0.9450602682223948
9	Cc1nc2cc(C)c3c([nH]c(=O)n3C)c2nc1C	0.5771	False	0.3856827655849179
13	Cc1nc2cc(C)c3c(nc(NO)n3C)c2nc1C	0.6900	True	0.9688695242313216
15	Cc1nc2cc(C(=O)O)c3[nH]c(N)nc3c2nc1C	0.1255	True	0.9370681306254384
17	Cc1nc2c(cc(C)c3[nH]c(N)nc32)nc1C(=O)O	0.3557	True	0.9371990578502958
18	Cc1nc2cc(CO)c3[nH]c(N)nc3c2nc1C	0.1425	True	0.945475586892378
20	Cc1nc2c(cc(C)c3[nH]c(N)nc32)nc1CO	0.3500	True	0.9456217987811018
21	Cc1nc2cc(C)c3[nH]c(=O)[nH]c3c2nc1C	0.2919	False	0.4254405686966013
25	Cc1nc2cc(C)c3[nH]c(NO)nc3c2nc1C	0.2148	True	0.966448534166162
37	Cc1nc2c(cc(C(=O)O)c3c2nc(N)n3C)nc1C(=O)O	0.2944	True	0.9138368184981132
38	Cc1nc2cc(C(=O)O)c3c(nc(N)n3C)c2nc1C(=O)O	0.2987	True	0.9022678720097804
39	Cc1nc2c(cc(C(=O)O)c3c2nc(N)n3C)nc1CO	0.2868	True	0.926430934305395

40	Cc1nc2cc(C(=O)O)c3c(nc(N)n3C)c2nc1CO	0.2900	True	0.9166227608850012
42	Cc1nc2cc(C(=O)O)c3c([nH]c(=O)n3C)c2nc1C	0.5734	False	0.2955217825470901
46	Cc1nc2cc(C(=O)O)c3c(nc(NO)n3C)c2nc1C	0.5589	True	0.9622959616671906
47	Cc1cc2nc(C(=O)O)c(C(=O)O)nc2c2nc(N)n(C)c12	0.3176	True	0.9138368143328124
48	Cc1cc2nc(CO)c(C(=O)O)nc2c2nc(N)n(C)c12	0.2942	True	0.9264309308723871
49	Cc1nc2cc(CO)c3c(nc(N)n3C)c2nc1C(=O)O	0.2920	True	0.916622757113866
51	Cc1nc2cc(C)c3c([nH]c(=O)n3C)c2nc1C(=O)O	0.5059	False	0.3472195794616843
55	Cc1nc2cc(C)c3c(nc(NO)n3C)c2nc1C(=O)O	0.5997	True	0.9622959606347984
56	Cc1cc2nc(C(=O)O)c(CO)nc2c2nc(N)n(C)c12	0.3050	True	0.9264309308723871
57	Cc1nc2c(cc(CO)c3c2nc(N)n3C)nc1C(=O)O	0.2819	True	0.9264309309932304
58	Cc1cnc2cc(C)c3c(nc(N)n3C)c2n1	0.2136	True	0.9527193370927972
59	Cc1nc2c(cc(C)c3c2[nH]c(=O)n3C)nc1C(=O)O	0.6618	False	0.2793007631212143
62	Cc1nc2c(cc(C)c3c2[n+]([O-])c(N)n3C)nc1C(=O)O	0.1133	True	0.9332082812204421
63	Cc1nc2c(cc(C)c3c2nc(NO)n3C)nc1C(=O)O	0.6399	True	0.962971720228064
64	Cc1nc2c(cc(CO)c3c2nc(N)n3C)nc1CO	0.2742	True	0.9366974574009704
65	Cc1nc2cc(CO)c3c(nc(N)n3C)c2nc1CO	0.2833	True	0.9283426870883372
66	Cc1nc2cc(CO)c3c([nH]c(=O)n3C)c2nc1C	0.5930	False	0.7455365925176584
70	Cc1nc2cc(CO)c3c(nc(NO)n3C)c2nc1C	0.5792	True	0.9657085043560121
71	Cc1cc2nc(CO)c(CO)nc2c2nc(N)n(C)c12	0.2812	True	0.936697457301072
72	Cc1nc2cc(C)c3c([nH]c(=O)n3C)c2nc1CO	0.4615	False	0.8193547082416229
76	Cc1nc2cc(C)c3c(nc(NO)n3C)c2nc1CO	0.5638	True	0.9657085043252516
77	Cc1nc2c(cc(C)c3c2[nH]c(=O)n3C)nc1CO	0.6215	False	0.8185217294546052
80	Cc1nc2c(cc(C)c3c2[n+]([O-])c(N)n3C)nc1CO	0.1009	True	0.941109202007716
81	Cc1nc2c(cc(C)c3c2nc(NO)n3C)nc1CO	0.5969	True	0.9663466347214794
82	Cc1nc2cc(C)c3c([nH]c(=O)n3C)c2[n+]([O-])c1C	0.1329	False	0.5036994480756635
83	Cc1nc2c3[nH]c(=O)n(C)c3c(C)cc2[n+]([O-])c1C	0.1200	False	0.538418109175139
90	Cc1nc2cc(C)c3c(nc(NC4OC(C(=O)O)C(O)C(O)C4O)n3C)c2nc1C	0.7340	False	0.13837005958543425
96	Cc1nc2cc(C)c3[nH]c(NC4OC(C(=O)O)C(O)C(O)C4O)nc3c2nc1C	0.1732	False	0.1387371709508488

99	Cc1nc2c3nc(N)[nH]c3c(C)cc2[n+](C2OC(C(=O)O)C(O)C(O)C2O)c1C	0.1961	True	0.8510328768395194
111	Cc1nc2cc(C(=O)OC3OC(C(=O)O)C(O)C(O)C3O)c3c(nc(N)n3C)c2nc1C	0.6175	False	0.7457106445531515
112	Cc1nc2cc(C(=O)O)c3c(nc(NC4OC(C(=O)O)C(O)C(O)C4O)n3C)c2nc1C	0.5593	False	0.09681831803413032
114	Cc1nc2cc(C(=O)O)c3c(c2nc1C)[n+](C1OC(C(=O)O)C(O)C(O)C1O)c(N)n3C	0.1139	False	0.7888191176469449
116	Cc1nc2cc(C(=O)O)c3c(nc(NS(=O)(=O)O)n3C)c2nc1C	0.3593	False	0.17689654946988007
117	CC(=O)Nc1nc2c3nc(C)c(C)nc3cc(C(=O)O)c2n1C	0.4884	False	0.28902633542034223
118	Cc1nc2cc(C)c3c(nc(N)n3C)c2nc1C(=O)OC1OC(C(=O)O)C(O)C(O)C1O	0.4647	False	0.7851814314058655
119	Cc1nc2cc(C)c3c(nc(NC4OC(C(=O)O)C(O)C(O)C4O)n3C)c2nc1C(=O)O	0.5010	False	0.0968183140947095
122	Cc1nc2cc(C)c3c(c2nc1C(=O)O)[n+](C1OC(C(=O)O)C(O)C(O)C1O)c(N)n3C	0.1452	False	0.7888191098064813
123	Cc1nc2cc(C)c3c(nc(NS(=O)(=O)O)n3C)c2nc1C(=O)O	0.3461	False	0.2249407154685828
124	CC(=O)Nc1nc2c3nc(C(=O)O)c(C)nc3cc(C)c2n1C	0.4671	False	0.28976940727666495
125	Cc1nc2c(cc(C)c3c2nc(N)n3C)nc1C(=O)OC1OC(C(=O)O)C(O)C(O)C1O	0.4861	False	0.7852704398378394
126	Cc1nc2c(cc(C)c3c2nc(NC2OC(C(=O)O)C(O)C(O)C2O)n3C)nc1C(=O)O	0.5208	False	0.10589644229212053
129	Cc1nc2c(cc(C)c3c2[n+](C2OC(C(=O)O)C(O)C(O)C2O)c(N)n3C)nc1C(=O)O	0.1562	False	0.8017170182277169
130	Cc1nc2c(cc(C)c3c2nc(NS(=O)(=O)O)n3C)nc1C(=O)O	0.3646	False	0.18364682721014144
131	CC(=O)Nc1nc2c3nc(C)c(C(=O)O)nc3cc(C)c2n1C	0.4787	False	0.2890263250501654
132	Cc1nc2cc(COC3OC(C(=O)O)C(O)C(O)C3O)c3c(nc(N)n3C)c2nc1C	0.5313	False	0.7909614324400446
133	Cc1nc2cc(CO)c3c(nc(NC4OC(C(=O)O)C(O)C(O)C4O)n3C)c2nc1C	0.5193	False	0.4532113173412775
134	Cc1nc2c3nc(N)n(C)c3c(CO)cc2[n+](C2OC(C(=O)O)C(O)C(O)C2O)c1C	0.1010	False	0.822721280524058
135	Cc1nc2cc(CO)c3c(c2nc1C)[n+](C1OC(C(=O)O)C(O)C(O)C1O)c(N)n3C	0.1058	False	0.8185963707547755
137	Cc1nc2cc(CO)c3c(nc(NS(=O)(=O)O)n3C)c2nc1C	0.3462	False	0.6353760737667271
138	Cc1nc2cc(COS(=O)(=O)O)c3c(nc(N)n3C)c2nc1C	0.5577	True	0.9362816567048096
139	CC(=O)Nc1nc2c3nc(C)c(C)nc3cc(CO)c2n1C	0.4640	False	0.6882510334487474
140	Cc1nc2cc(C)c3c(nc(N)n3C)c2nc1COC1OC(C(=O)O)C(O)C(O)C1O	0.4107	False	0.8242326161539762
141	Cc1nc2cc(C)c3c(nc(NC4OC(C(=O)O)C(O)C(O)C4O)n3C)c2nc1CO	0.4571	False	0.6104779437776809
143	Cc1cc2c(nc(CO)c(C)[n+]2C2OC(C(=O)O)C(O)C(O)C2O)c2nc(N)n(C)c12	0.1148	False	0.8227212802089878
144	Cc1nc2cc(C)c3c(c2nc1CO)[n+](C1OC(C(=O)O)C(O)C(O)C1O)c(N)n3C	0.1281	False	0.8185963705156074
145	Cc1nc2cc(C)c3c(nc(NS(=O)(=O)O)n3C)c2nc1CO	0.3157	False	0.7492928735688927

146	Cc1nc2cc(C)c3c(nc(N)n3C)c2nc1COS(=O)(=O)O	0.4681	True	0.9579709478002996
147	CC(=O)Nc1nc2c3nc(CO)c(C)nc3cc(C)c2n1C	0.4261	False	0.7879146520038296
148	Cc1nc2c(cc(C)c3c2nc(N)n3C)nc1COC1OC(C(=O)O)C(O)C(O)C1O	0.4217	False	0.8243413148736591
149	Cc1nc2c(cc(C)c3c2nc(NC2OC(C(=O)O)C(O)C(O)C2O)n3C)nc1CO	0.4637	False	0.6089069790986317
152	Cc1nc2c(cc(C)c3c2[n+](C2OC(C(=O)O)C(O)C(O)C2O)c(N)n3C)nc1CO	0.1413	False	0.8299753219232127
153	Cc1nc2c(cc(C)c3c2nc(NS(=O)(=O)O)n3C)nc1CO	0.3246	False	0.7482482571695571
154	Cc1nc2c(cc(C)c3c2nc(N)n3C)nc1COS(=O)(=O)O	0.4725	True	0.957809790047806
155	CC(=O)Nc1nc2c3nc(C)c(CO)nc3cc(C)c2n1C	0.4261	False	0.7869881850369813
157	Cc1nc2c3[nH]c(=O)n(C)c3c(C)cc2[n+](C2OC(C(=O)O)C(O)C(O)C2O)c1C	0.1677	False	0.12733594423837818
174	Cc1nc2cc(C)c3c(nc(NOC4OC(C(=O)O)C(O)C(O)C4O)n3C)c2nc1C	0.2981	True	0.9394393402141152
175	Cc1nc2cc(C)c3c(nc(N(O)C4OC(C(=O)O)C(O)C(O)C4O)n3C)c2nc1C	0.2042	False	0.4351992592542831
178	Cc1nc2c3nc(NO)n(C)c3c(C)cc2[n+](C2OC(C(=O)O)C(O)C(O)C2O)c1C	0.1380	True	0.9298838804031396

4-CH2OH-8-MeIQx

Metabolites_ID	SMILES_Formula	Production probability score	Reactive_to_DNA (>=0.85)	XenoSite Reactivity score
0	Cc1cnc2cc(CO)c3c(nc(N)n3C)c2n1	1.0000	True	0.939246545782168
1	Cc1cnc2cc(CO)c3[nH]c(N)nc3c2n1	0.6940	True	0.9399486976355284
4	Cn1c(N)nc2c3nc(C(=O)O)cnc3cc(CO)c21	0.7416	True	0.9183886343549283
5	Cn1c(N)nc2c3nc(CO)cnc3cc(CO)c21	0.8330	True	0.9297950666666231
6	Cc1cnc2cc(C(=O)O)c3c(nc(N)n3C)c2n1	0.7970	True	0.9297634019142198
7	Cc1cnc2cc(CO)c3c([nH]c(=O)n3C)c2n1	0.2681	False	0.7525994388021336
11	Cc1cnc2cc(CO)c3c(nc(NO)n3C)c2n1	0.4870	True	0.9661762365971523
12	Cc1nc2c(cc(CO)c3[nH]c(N)nc32)nc1O	0.1915	True	0.9396626815064018
14	Nc1nc2c([nH]1)c(CO)cc1ncc(C(=O)O)nc12	0.3061	True	0.9192069137181936
15	Nc1nc2c([nH]1)c(CO)cc1ncc(CO)nc12	0.3022	True	0.9304529094585507
16	Cc1cnc2cc(C(=O)O)c3[nH]c(N)nc3c2n1	0.1206	True	0.9306062036714235
17	Cc1cnc2cc(CO)c3[nH]c(=O)[nH]c3c2n1	0.1314	False	0.7344730497861981

21	Cc1cnc2cc(CO)c3[nH]c(NO)nc3c2n1	0.1632	True	0.9633861582056056
24	Cn1c(N)nc2c3nc(CO)c(O)nc3cc(CO)c21	0.1466	True	0.9329028728863292
25	Cc1nc2c(cc(C(=O)O)c3c2nc(N)n3C)nc1O	0.1782	True	0.9295012404074886
26	Cc1nc2c(cc(CO)c3c2[nH]c(=O)n3C)nc1O	0.1388	False	0.7367474363970398
30	Cc1nc2c(cc(CO)c3c2nc(NO)n3C)nc1O	0.1500	True	0.9631115497659792
39	Cn1c(N)nc2c3nccnc3cc(CO)c21	0.3137	True	0.9470328096060072
40	Cn1c(N)nc2c3nc(C(=O)O)cnc3cc(C(=O)O)c21	0.3607	True	0.9044177826890109
41	Cn1c(=O)[nH]c2c3nc(C(=O)O)cnc3cc(CO)c21	0.5381	False	0.6968627824778062
44	Cn1c(N)[n+]([O-])c2c3nc(C(=O)O)cnc3cc(CO)c21	0.1470	True	0.9150345060270454
45	Cn1c(NO)nc2c3nc(C(=O)O)cnc3cc(CO)c21	0.5346	True	0.9585368267450812
46	Cn1c(N)nc2c3nc(CO)cnc3cc(C(=O)O)c21	0.3556	True	0.9183886380336036
47	Cn1c(=O)[nH]c2c3nc(CO)cnc3cc(CO)c21	0.5181	False	0.813710764792595
50	Cn1c(N)[n+]([O-])c2c3nc(CO)cnc3cc(CO)c21	0.1224	True	0.9252476842839548
51	Cn1c(NO)nc2c3nc(CO)cnc3cc(CO)c21	0.5145	True	0.9624737595749632
53	Cc1cnc2cc(C(=O)O)c3c([nH]c(=O)n3C)c2n1	0.4601	False	0.3076820818757737
54	Cc1c[n+]([O-])c2cc(C(=O)O)c3c(nc(N)n3C)c2n1	0.1782	True	0.9214313394054104
57	Cc1cnc2cc(C(=O)O)c3c(nc(NO)n3C)c2n1	0.1829	True	0.9628554874324928
66	Cc1cnc2cc(COC3OC(C(=O)O)C(O)C(O)C3O)c3c(nc(N)n3C)c2n1	0.1430	False	0.7959041892790614
67	Cc1cnc2cc(CO)c3c(nc(NC4OC(C(=O)O)C(O)C(O)C4O)n3C)c2n1	0.4470	False	0.4781837291519712
74	Cc1cnc2cc(COC3OC(C(=O)O)C(O)C(O)C3O)c3[nH]c(N)nc3c2n1	0.6190	False	0.7974615695700105
78	Cc1c[n+](C2OC(C(=O)O)C(O)C(O)C2O)c2cc(CO)c3[nH]c(N)nc3c2n1	0.1277	False	0.8281445139036444
81	Cc1cnc2cc(COS(=O)(=O)O)c3[nH]c(N)nc3c2n1	0.5996	True	0.9281737677695644
103	Cn1c(N)nc2c3nc(C(=O)O)cnc3cc(COC3OC(C(=O)O)C(O)C(O)C3O)c21	0.7856	False	0.7034278531573016
104	Cn1c(N)nc2c3nc(C(=O)OC4OC(C(=O)O)C(O)C(O)C4O)cnc3cc(CO)c21	0.6940	False	0.7361105200113731
105	Cn1c(NC2OC(C(=O)O)C(O)C(O)C2O)nc2c3nc(C(=O)O)cnc3cc(CO)c21	0.6482	False	0.3648961922925914
108	Cn1c(N)[n+](C2OC(C(=O)O)C(O)C(O)C2O)c2c3nc(C(=O)O)cnc3cc(CO)c21	0.1409	False	0.7562250686735003
109	Cn1c(NS(=O)(=O)O)nc2c3nc(C(=O)O)cnc3cc(CO)c21	0.4509	False	0.5801416341311411
110	Cn1c(N)nc2c3nc(C(=O)O)cnc3cc(COS(=O)(=O)O)c21	0.7187	True	0.9200551274244104

111	CC(=O)Nc1nc2c3nc(C(=O)O)cnc3cc(CO)c2n1C	0.6341	False	0.6402471927586828
112	Cn1c(N)nc2c3nc(COC4OC(C(=O)O)C(O)C(O)C4O)cnc3cc(CO)c21	0.6497	False	0.7826073222968977
113	Cn1c(N)nc2c3nc(CO)cnc3cc(COC3OC(C(=O)O)C(O)C(O)C3O)c21	0.7430	False	0.7474417380900512
114	Cn1c(NC2OC(C(=O)O)C(O)C(O)C2O)nc2c3nc(CO)cnc3cc(CO)c21	0.6131	False	0.5840922307976594
116	Cn1c(N)nc2c3nc(CO)c[n+](C4OC(C(=O)O)C(O)C(O)C4O)c3cc(CO)c21	0.1433	False	0.7957213472607796
117	Cn1c(N)[n+](C2OC(C(=O)O)C(O)C(O)C2O)c2c3nc(CO)cnc3cc(CO)c21	0.1266	False	0.7899565344739715
118	Cn1c(NS(=O)(=O)O)nc2c3nc(CO)cnc3cc(CO)c21	0.4265	False	0.7389240175157654
119	Cn1c(N)nc2c3nc(COS(=O)(=O)O)cnc3cc(CO)c21	0.7031	True	0.9513218613965329
120	Cn1c(N)nc2c3nc(CO)cnc3cc(COS(=O)(=O)O)c21	0.6797	True	0.9301629974208848
121	CC(=O)Nc1nc2c3nc(CO)cnc3cc(CO)c2n1C	0.5998	False	0.780440363453988
122	Cc1cnc2cc(C(=O)OC3OC(C(=O)O)C(O)C(O)C3O)c3c(nc(N)n3C)c2n1	0.7715	False	0.7516321470738067
123	Cc1cnc2cc(C(=O)O)c3c(nc(NC4OC(C(=O)O)C(O)C(O)C4O)n3C)c2n1	0.6025	False	0.09934650446541436
125	Cc1cnc2cc(C(=O)O)c3c(c2n1)[n+](C1OC(C(=O)O)C(O)C(O)C1O)c(N)n3C	0.1307	False	0.7929786868238702
127	Cc1cnc2cc(C(=O)O)c3c(nc(NS(=O)(=O)O)n3C)c2n1	0.3762	False	0.187152862044287
128	CC(=O)Nc1nc2c3nc(C)cnc3cc(C(=O)O)c2n1C	0.5675	False	0.2787233326697437
129	Cc1cnc2cc(COC3OC(C(=O)O)C(O)C(O)C3O)c3c([nH]c(=O)n3C)c2n1	0.4165	False	0.6179684916244721
133	Cc1cnc2cc(COS(=O)(=O)O)c3c([nH]c(=O)n3C)c2n1	0.4683	True	0.9331380220338998
155	Cc1cnc2cc(COC3OC(C(=O)O)C(O)C(O)C3O)c3c(nc(NO)n3C)c2n1	0.4325	True	0.9186227958903936
156	Cc1cnc2cc(CO)c3c(nc(NOC4OC(C(=O)O)C(O)C(O)C4O)n3C)c2n1	0.2104	True	0.9187100465128704
157	Cc1cnc2cc(CO)c3c(nc(N(O)C4OC(C(=O)O)C(O)C(O)C4O)n3C)c2n1	0.1442	False	0.4397655038488933
161	Cc1cnc2cc(COS(=O)(=O)O)c3c(nc(NO)n3C)c2n1	0.4539	True	0.9528983217648508
162	Cc1cnc2cc(COC3OC(C(=O)O)C(O)C(O)C3O)c3c(nc(NC4OC(C(=O)O)C(O)C(O)C4O)n3C)c2n1	0.4305	False	0.2674484837322748
172	Cc1cnc2cc(COS(=O)(=O)O)c3c(nc(NC4OC(C(=O)O)C(O)C(O)C4O)n3C)c2n1	0.4327	False	0.8236623799216444

7,8-DiMeIQx

Metabolites_ID	SMILES_Formula	Production probability score	Reactive_to_DNA (>=0.85)	XenoSite Reactivity score
0	Cc1nc2ccc3c(nc(N)n3C)c2nc1C	1.0000	True	0.9526303225571204

1	Cc1nc2ccc3[nH]c(N)nc3c2nc1C	0.9130	True	0.9532412905725546
3	Cc1nc2cc(O)c3c(nc(N)n3C)c2nc1C	0.3300	True	0.947257793228356
4	Cc1nc2ccc3c(nc(N)n3C)c2nc1C(=O)O	0.3974	True	0.937552266604346
5	Cc1nc2c(ccc3c2nc(N)n3C)nc1C(=O)O	0.4111	True	0.937552266604346
6	Cc1nc2ccc3c(nc(N)n3C)c2nc1CO	0.5860	True	0.945917356926128
7	Cc1nc2c(ccc3c2nc(N)n3C)nc1CO	0.5860	True	0.945917356926128
8	Cc1nc2ccc3c([nH]c(=O)n3C)c2nc1C	0.6472	False	0.3931118524235226
12	Cc1nc2ccc3c(nc(NO)n3C)c2nc1C	0.7390	True	0.9692538972925264
13	Cc1nc2c(O)cc3[nH]c(N)nc3c2nc1C	0.1132	True	0.947622997351468
14	Cc1nc2cc(O)c3[nH]c(N)nc3c2nc1C	0.1413	True	0.947813590358718
15	Cc1nc2ccc3[nH]c(N)nc3c2nc1C(=O)O	0.3195	True	0.9382566407991466
16	Cc1nc2c(ccc3[nH]c(N)nc32)nc1C(=O)O	0.4326	True	0.9382566407991466
18	Cc1nc2c(ccc3[nH]c(N)nc32)nc1CO	0.4233	True	0.946491592095027
19	Cc1nc2ccc3[nH]c(=O)[nH]c3c2nc1C	0.7965	False	0.433071014730353
23	Cc1nc2ccc3[nH]c(NO)nc3c2nc1C	0.2631	True	0.9668948123415207
26	Cc1nc2c(O)cc3c(nc(N)n3C)c2nc1C(=O)O	0.0922	True	0.9293640973340352
29	Cc1nc2c(O)cc3c([nH]c(=O)n3C)c2nc1C	0.0370	False	0.3642730234787643
33	Cc1nc2c(O)cc3c(nc(NO)n3C)c2nc1C	0.0562	True	0.966706651341194
34	Cc1nc2cc(O)c3c(nc(N)n3C)c2nc1C(=O)O	0.1149	True	0.9296412848269409
35	Cc1nc2c(cc(O)c3c2nc(N)n3C)nc1C(=O)O	0.1676	True	0.9296412848269409
36	Cc1nc2cc(O)c3c(nc(N)n3C)c2nc1CO	0.1147	True	0.9393408281727236
37	Cc1nc2c(cc(O)c3c2nc(N)n3C)nc1CO	0.1549	True	0.9393408281727236
38	Cc1nc2cc(O)c3c([nH]c(=O)n3C)c2nc1C	0.1882	False	0.36207412502176894
42	Cc1nc2cc(O)c3c(nc(NO)n3C)c2nc1C	0.1891	True	0.9667196195161194
43	Cn1c(N)nc2c3nc(C(=O)O)c(C(=O)O)nc3ccc21	0.3400	True	0.9153814781071824
44	Cn1c(N)nc2c3nc(C(=O)O)c(CO)nc3ccc21	0.3142	True	0.9276954450903158
45	Cc1cnc2c(ccc3c2nc(N)n3C)n1	0.2077	True	0.9534974792514564
46	Cc1nc2ccc3c([nH]c(=O)n3C)c2nc1C(=O)O	0.6144	False	0.3537918781352531

49	Cc1nc2ccc3c(c2nc1C(=O)O)[n+]([O-])c(N)n3C	0.1818	True	0.9340630817091758
50	Cc1nc2ccc3c(nc(NO)n3C)c2nc1C(=O)O	0.6963	True	0.9628312566982632
51	Cn1c(N)nc2c3nc(CO)c(C(=O)O)nc3ccc21	0.3291	True	0.927695445090316
52	Cc1cnc2ccc3c(nc(N)n3C)c2n1	0.2147	True	0.9020011272283596
53	Cc1nc2c(ccc3c2[nH]c(=O)n3C)nc1C(=O)O	0.3320	False	0.28502616028699546
56	Cc1nc2c(ccc3c2[n+]([O-])c(N)n3C)nc1C(=O)O	0.1504	True	0.9340630817091758
57	Cc1nc2c(ccc3c2nc(NO)n3C)nc1C(=O)O	0.7151	True	0.9635006209839014
58	Cn1c(N)nc2c3nc(CO)c(CO)nc3ccc21	0.3029	True	0.9377346761986114
59	Cc1nc2ccc3c([nH]c(=O)n3C)c2nc1CO	0.5626	False	0.8229736559749736
62	Cc1nc2ccc3c(c2nc1CO)[n+]([O-])c(N)n3C	0.1231	True	0.9418315309031982
63	Cc1nc2ccc3c(nc(NO)n3C)c2nc1CO	0.6554	True	0.9661630273872972
64	Cc1nc2c(ccc3c2[nH]c(=O)n3C)nc1CO	0.6911	False	0.8221506428125687
67	Cc1nc2c(ccc3c2[n+]([O-])c(N)n3C)nc1CO	0.1349	True	0.9418315309031982
68	Cc1nc2c(ccc3c2nc(NO)n3C)nc1CO	0.6686	True	0.9667957493222371
69	Cc1nc2ccc3c([nH]c(=O)n3C)c2[n+]([O-])c1C	0.1411	False	0.5106579759858405
70	Cc1nc2c3[nH]c(=O)n(C)c3ccc2[n+]([O-])c1C	0.1579	False	0.545304641006959
77	Cc1nc2ccc3c(nc(NC4OC(C(=O)O)C(O)C(O)C4O)n3C)c2nc1C	0.7900	False	0.14128924533621762
83	Cc1nc2ccc3[nH]c(NC4OC(C(=O)O)C(O)C(O)C4O)nc3c2nc1C	0.1972	False	0.14163223897622915
84	Cc1nc2ccc3[nH]c(N)[n+](C4OC(C(=O)O)C(O)C(O)C4O)c3c2nc1C	0.1972	False	0.8496759833252281
85	Cc1nc2ccc3[nH]c(N)nc3c2[n+](C2OC(C(=O)O)C(O)C(O)C2O)c1C	0.1570	True	0.8541000543492518
86	Cc1nc2c3nc(N)[nH]c3ccc2[n+](C2OC(C(=O)O)C(O)C(O)C2O)c1C	0.2337	True	0.8540999219182439
87	Cc1nc2ccc3c(nc(N)n3C3OC(C(=O)O)C(O)C(O)C3O)c2nc1C	0.3177	False	0.8335984380668678
88	Cc1nc2ccc3[nH]c(NS(=O)(=O)O)nc3c2nc1C	0.5405	False	0.29014961211242624
89	CC(=O)Nc1nc2c(ccc3nc(C)c(C)nc32)[nH]1	0.7231	False	0.39624710208252295
98	Cc1nc2cc(OC3OC(C(=O)O)C(O)C(O)C3O)c3c(nc(N)n3C)c2nc1C	0.2086	False	0.7975230911352285
99	Cc1nc2cc(O)c3c(nc(NC4OC(C(=O)O)C(O)C(O)C4O)n3C)c2nc1C	0.1964	False	0.11980117236897055
103	Cc1nc2cc(OS(=O)(=O)O)c3c(nc(N)n3C)c2nc1C	0.2732	True	0.906323873862639
104	Cc1nc2cc(O)c3c(nc(NS(=O)(=O)O)n3C)c2nc1C	0.1214	False	0.23067120891923004

105	CC(=O)Nc1nc2c3nc(C)c(C)nc3cc(O)c2n1C	0.2442	False	0.3488387270156466
106	Cc1nc2ccc3c(nc(N)n3C)c2nc1C(=O)OC1OC(C(=O)O)C(O)C(O)C1O	0.4915	False	0.7895415567149885
107	Cc1nc2ccc3c(nc(NC4OC(C(=O)O)C(O)C(O)C4O)n3C)c2nc1C(=O)O	0.5735	False	0.09902060770467656
110	Cc1nc2ccc3c(c2nc1C(=O)O)[n+](C1OC(C(=O)O)C(O)C(O)C1O)c(N)n3C	0.2278	False	0.7922689419572471
111	Cc1nc2ccc3c(nc(NS(=O)(=O)O)n3C)c2nc1C(=O)O	0.3866	False	0.2303419735567399
112	CC(=O)Nc1nc2c3nc(C(=O)O)c(C)nc3ccc2n1C	0.4967	False	0.2959348465199415
113	Cc1nc2c(ccc3c2nc(N)n3C)nc1C(=O)OC1OC(C(=O)O)C(O)C(O)C1O	0.5125	False	0.7896259199915192
114	Cc1nc2c(ccc3c2nc(NC2OC(C(=O)O)C(O)C(O)C2O)n3C)nc1C(=O)O	0.5857	False	0.10831323730539713
117	Cc1nc2c(ccc3c2[n+](C2OC(C(=O)O)C(O)C(O)C2O)c(N)n3C)nc1C(=O)O	0.2013	False	0.804986177950973
118	Cc1nc2c(ccc3c2nc(NS(=O)(=O)O)n3C)nc1C(=O)O	0.4027	False	0.18819526740987594
119	CC(=O)Nc1nc2c3nc(C)c(C(=O)O)nc3ccc2n1C	0.5073	False	0.2951971305132569
120	Cc1nc2ccc3c(nc(N)n3C)c2nc1COC1OC(C(=O)O)C(O)C(O)C1O	0.4360	False	0.8278196414052801
121	Cc1nc2ccc3c(nc(NC4OC(C(=O)O)C(O)C(O)C4O)n3C)c2nc1CO	0.5251	False	0.6177843899021765
123	Cc1c(CO)nc2c3nc(N)n(C)c3ccc2[n+]1C1OC(C(=O)O)C(O)C(O)C1O	0.1406	False	0.8263333282328998
124	Cc1nc2ccc3c(c2nc1CO)[n+](C1OC(C(=O)O)C(O)C(O)C1O)c(N)n3C	0.1641	False	0.8215309603633622
125	Cc1nc2ccc3c(nc(NS(=O)(=O)O)n3C)c2nc1CO	0.3539	False	0.7544096199052549
126	Cc1nc2ccc3c(nc(N)n3C)c2nc1COS(=O)(=O)O	0.4969	True	0.958565947543656
127	CC(=O)Nc1nc2c3nc(CO)c(C)nc3ccc2n1C	0.4547	False	0.7922359747966528
128	Cc1nc2c(ccc3c2nc(N)n3C)nc1COC1OC(C(=O)O)C(O)C(O)C1O	0.4477	False	0.8279253001213381
129	Cc1nc2c(ccc3c2nc(NC2OC(C(=O)O)C(O)C(O)C2O)n3C)nc1CO	0.5251	False	0.6162198140862697
130	Cc1nc2c3nc(N)n(C)c3ccc2[n+](C2OC(C(=O)O)C(O)C(O)C2O)c1CO	0.1078	False	0.8263333282328998
132	Cc1nc2c(ccc3c2[n+](C2OC(C(=O)O)C(O)C(O)C2O)c(N)n3C)nc1CO	0.1805	False	0.8327448843846529
133	Cc1nc2c(ccc3c2nc(NS(=O)(=O)O)n3C)nc1CO	0.3586	False	0.7533741708342333
134	Cc1nc2c(ccc3c2nc(N)n3C)nc1COS(=O)(=O)O	0.5016	True	0.9584058263601922
135	CC(=O)Nc1nc2c3nc(C)c(CO)nc3ccc2n1C	0.4547	False	0.7913194225854521
136	Cc1nc2ccc3c([nH]c(=O)n3C)c2[n+](C2OC(C(=O)O)C(O)C(O)C2O)c1C	0.1378	False	0.13163256054954975
137	Cc1nc2c3[nH]c(=O)n(C)c3ccc2[n+](C2OC(C(=O)O)C(O)C(O)C2O)c1C	0.2016	False	0.1306499178923529
138	Cc1nc2ccc3c(c2nc1C)n(C1OC(C(=O)O)C(O)C(O)C1O)c(=O)n3C	0.1546	False	0.09426602764544464

154	Cc1nc2ccc3c(nc(NOC4OC(C(=O)O)C(O)C(O)C4O)n3C)c2nc1C	0.3163	True	0.940142849022794
155	Cc1nc2ccc3c(nc(N(O)C4OC(C(=O)O)C(O)C(O)C4O)n3C)c2nc1C	0.2187	False	0.4418016730104229
156	Cc1nc2ccc3c(c2nc1C)[n+](C1OC(C(=O)O)C(O)C(O)C1O)c(NO)n3C	0.1301	True	0.9479245326875604
158	Cc1nc2c3nc(NO)n(C)c3ccc2[n+](C2OC(C(=O)O)C(O)C(O)C2O)c1C	0.1803	True	0.9312877406288887
162	Cc1nc2c3nc(NC4OC(C(=O)O)C(O)C(O)C4O)n(C)c3ccc2[n+](C2OC(C(=O)O)C(O)C(O)C2O)c1C	0.1264	False	0.04152612811541051

AalphaC

Metabolites_ID	SMILES_Formula	Production probability score	Reactive_to_DNA (>=0.85)	XenoSite Reactivity score
0	Nc1ccc2c(n1)[nH]c1ccccc12	1.0000	True	0.8594642870288121
1	Nc1ccc2c(n1)[nH]c1cc(O)ccc12	0.3620	True	0.9178122922308036
2	Nc1nc2[nH]c3ccccc3c2cc1O	0.3850	True	0.9104374669419212
3	Nc1ccc2c(n1)[nH]c1ccc(O)cc12	0.4980	True	0.9178122922308036
4	Nc1ccc2c(n1)[nH]c1c(O)cccc12	0.2050	True	0.9173389807704077
5	O=c1ccc2c([nH]1)[nH]c1ccccc12	0.3483	False	0.07521168888614659
7	ONc1ccc2c(n1)[nH]c1ccccc12	0.4700	True	0.9320796713748204
8	Nc1ccc2c(n1)[nH]c1cc(O)c(O)cc12	0.1246	True	0.9054297852222036
9	Nc1nc2[nH]c3cc(O)ccc3c2cc1O	0.3144	True	0.8971425081962023
11	O=c1ccc2c([nH]1)[nH]c1cc(O)ccc12	0.3786	False	0.04921904388986637
12	Nc1ccc2c3ccc(O)cc3[nH]c2n1O	0.1796	False	0.21362896295664915
13	ONc1ccc2c(n1)[nH]c1cc(O)ccc12	0.3743	True	0.9557483537545116
14	Nc1nc2[nH]c3ccc(O)cc3c2cc1O	0.4256	True	0.8977844487769387
15	Nc1nc2[nH]c3c(O)cccc3c2cc1O	0.1480	True	0.8971425081962023
17	O=c1[nH]c2[nH]c3ccccc3c2cc1O	0.3958	False	0.061405818683500576
19	ONc1nc2[nH]c3ccccc3c2cc1O	0.2380	True	0.9657290904460372
22	O=c1ccc2c([nH]1)[nH]c1ccc(O)cc12	0.6273	False	0.1004533015347112
23	Nc1ccc2c3cc(O)ccc3[nH]c2n1O	0.2550	False	0.21362896295664915
24	ONc1ccc2c(n1)[nH]c1ccc(O)cc12	0.5408	True	0.9557483537545116

25	Nc1ccc2c(n1)N=C1C=CC(=O)C=C12	0.2868	False	0.8409736893124711
27	O=c1ccc2c([nH]1)[nH]c1c(O)cccc12	0.2344	False	0.08511848795086216
29	ONc1ccc2c(n1)[nH]c1c(O)cccc12	0.2490	True	0.9557483537545116
32	O=C(O)C1OC(Nc2ccc3c(n2)[nH]c2ccccc23)C(O)C(O)C1O	0.6880	False	0.06958169896151717
34	Nc1ccc2c3ccccc3n(C3OC(C(=O)O)C(O)C(O)C3O)c2n1	0.1280	False	0.7506542647056539
37	Nc1ccc2c(n1)[nH]c1cc(OC3OC(C(=O)O)C(O)C(O)C3O)ccc12	0.3606	False	0.7259178798306849
38	O=C(O)C1OC(Nc2ccc3c(n2)[nH]c2cc(O)ccc23)C(O)C(O)C1O	0.3446	False	0.05942302710234624
39	Nc1ccc2c3ccc(O)cc3[nH]c2n1C1OC(C(=O)O)C(O)C(O)C1O	0.2766	False	0.017567897784690138
40	Nc1ccc2c3ccc(O)cc3n(C3OC(C(=O)O)C(O)C(O)C3O)c2n1	0.1520	False	0.7110168267507706
41	Nc1ccc2c(n1)[nH]c1cc(OS(=O)(=O)O)ccc12	0.3548	True	0.8725659998255538
42	O=S(=O)(O)Nc1ccc2c(n1)[nH]c1cc(O)ccc12	0.2664	False	0.08317776219627293
43	CC(=O)Nc1ccc2c(n1)[nH]c1cc(O)ccc12	0.3446	False	0.1868702590928962
44	Nc1nc2[nH]c3ccccc3c2cc1OC1OC(C(=O)O)C(O)C(O)C1O	0.2988	False	0.6744863352644157
45	O=C(O)C1OC(Nc2nc3[nH]c4ccccc4c3cc2O)C(O)C(O)C1O	0.3203	False	0.06661743405262767
46	Nc1c(O)cc2c3ccccc3[nH]c2n1C1OC(C(=O)O)C(O)C(O)C1O	0.2402	False	0.050903698198718726
47	Nc1nc2c(cc1O)c1ccccc1n2C1OC(C(=O)O)C(O)C(O)C1O	0.1586	False	0.6904836807414154
48	Nc1nc2[nH]c3ccccc3c2cc1OS(=O)(=O)O	0.3696	True	0.8976396777784863
49	O=S(=O)(O)Nc1nc2[nH]c3ccccc3c2cc1O	0.2880	False	0.07463840074684512
50	CC(=O)Nc1nc2[nH]c3ccccc3c2cc1O	0.3634	False	0.1850721970410205
51	Nc1ccc2c(n1)[nH]c1ccc(OC3OC(C(=O)O)C(O)C(O)C3O)cc12	0.4940	False	0.7259178798306849
52	O=C(O)C1OC(Nc2ccc3c(n2)[nH]c2ccc(O)cc23)C(O)C(O)C1O	0.4602	False	0.05942302710234624
53	Nc1ccc2c3cc(O)ccc3[nH]c2n1C1OC(C(=O)O)C(O)C(O)C1O	0.3745	False	0.017567897784690138
54	Nc1ccc2c3cc(O)ccc3n(C3OC(C(=O)O)C(O)C(O)C3O)c2n1	0.2171	False	0.6807681397450976
55	Nc1ccc2c(n1)[nH]c1ccc(OS(=O)(=O)O)cc12	0.4582	True	0.8725659998255538
56	O=S(=O)(O)Nc1ccc2c(n1)[nH]c1ccc(O)cc12	0.3625	False	0.07219347334092874
57	CC(=O)Nc1ccc2c(n1)[nH]c1ccc(O)cc12	0.4741	False	0.1868702590928962
58	Nc1ccc2c(n1)[nH]c1c(OC3OC(C(=O)O)C(O)C(O)C3O)cccc12	0.2009	False	0.7259178798306849
59	O=C(O)C1OC(Nc2ccc3c(n2)[nH]c2c(O)cccc23)C(O)C(O)C1O	0.1960	False	0.05942302710234624

60	Nc1ccc2c3cccc(O)c3[nH]c2n1C1OC(C(=O)O)C(O)C(O)C1O	0.1205	False	0.017567897784690138
62	Nc1ccc2c(n1)[nH]c1c(OS(=O)(=O)O)cccc12	0.1960	True	0.8721892283792341
63	O=S(=O)(O)Nc1ccc2c(n1)[nH]c1c(O)cccc12	0.1501	False	0.07528770637472898
64	CC(=O)Nc1ccc2c(n1)[nH]c1c(O)cccc12	0.1952	False	0.17505455742338466
65	O=C(O)C1OC(n2c(=O)ccc3c4ccccc4[nH]c32)C(O)C(O)C1O	0.1768	False	0.05114923693441379
66	O=C(O)C1OC(n2c3ccccc3c3ccc(=O)[nH]c32)C(O)C(O)C1O	0.1434	False	0.036301666725559366
71	O=C(O)C1OC(ONc2ccc3c(n2)[nH]c2ccccc23)C(O)C(O)C1O	0.1861	True	0.9004352701152516
72	O=C(O)C1OC(N(O)c2ccc3c(n2)[nH]c2ccccc23)C(O)C(O)C1O	0.2275	False	0.27448964713364665
73	O=C(O)C1OC(n2c(NO)ccc3c4ccccc4[nH]c32)C(O)C(O)C1O	0.2106	False	0.34194329742413737
74	O=C(O)C1OC(n2c3ccccc3c3ccc(NO)nc32)C(O)C(O)C1O	0.2425	True	0.9100635799042868
77	O=C(O)C1OC(Nc2ccc3c4ccccc4n(C4OC(C(=O)O)C(O)C(O)C4O)c3n2)C(O)C(O)C1O	0.3735	False	0.02091430158696055
82	CC(=O)Nc1ccc2c3ccccc3n(C3OC(C(=O)O)C(O)C(O)C3O)c2n1	0.1224	False	0.04508733890936721

MeIQx

Metabolites_ID	SMILES_Formula	Production probability score	Reactive_to_DNA (>=0.85)	XenoSite Reactivity score
0	Cc1cnc2ccc3c(nc(N)n3C)c2n1	1.0000	True	0.9020011272283596
1	Cc1cnc2ccc3[nH]c(N)nc3c2n1	0.8660	True	0.9540024480766478
3	Cc1cnc2cc(O)c3c(nc(N)n3C)c2n1	0.3290	True	0.9481434830784878
4	Cc1cnc2c(O)cc3c(nc(N)n3C)c2n1	0.1080	True	0.9479487042622924
5	Cn1c(N)nc2c3nc(C(=O)O)cnc3ccc21	0.8479	True	0.9388094727833032
6	Cn1c(N)nc2c3nc(CO)cnc3ccc21	0.9070	True	0.9469520865325473
7	Cc1cnc2ccc3c([nH]c(=O)n3C)c2n1	0.5417	False	0.418435141090156
11	Cc1cnc2ccc3c(nc(NO)n3C)c2n1	0.6500	True	0.9498784008521852
12	Cc1nc2c(ccc3[nH]c(N)nc32)nc1O	0.2425	True	0.9486334206121112
14	Cc1cnc2cc(O)c3[nH]c(N)nc3c2n1	0.1131	True	0.9486803877383232
15	Nc1nc2c(ccc3ncc(C(=O)O)nc32)[nH]1	0.4157	True	0.9395171969398016
16	Nc1nc2c(ccc3ncc(CO)nc32)[nH]1	0.4176	True	0.9475300341434734

17	Cc1cnc2ccc3[nH]c(=O)[nH]c3c2n1	0.7647	False	0.4592935267484921
18	Cc1cnc2ccc3[nH]c(N)n(O)c3c2n1	0.1760	False	0.5843451083119229
19	Cc1cnc2ccc3[nH]c(N)nc3c2n1O	0.1178	False	0.40200726318413016
21	Cc1cnc2ccc3[nH]c(NO)nc3c2n1	0.7134	True	0.9673234151486991
25	Cn1c(N)nc2c3nc(CO)c(O)nc3ccc21	0.1270	True	0.9403177904594091
26	Cc1nc2c(ccc3c2[nH]c(=O)n3C)nc1O	0.2154	False	0.5632902743933411
30	Cc1nc2c(ccc3c2nc(NO)n3C)nc1O	0.1607	True	0.9671489112527334
32	Cn1c(N)nc2c3nc(C(=O)O)cnc3cc(O)c21	0.1462	True	0.931103786031207
33	Cn1c(N)nc2c3nc(CO)cnc3cc(O)c21	0.1453	True	0.9405427932760242
34	Cc1cnc2cc(O)c3c([nH]c(=O)n3C)c2n1	0.2293	False	0.3854993100237397
38	Cc1cnc2cc(O)c3c(nc(NO)n3C)c2n1	0.1997	True	0.967161448656272
39	Cn1c(N)nc2c3nc(C(=O)O)cnc3c(O)cc21	0.1728	True	0.9308316163365276
40	Cn1c(N)nc2c3nc(CO)cnc3c(O)cc21	0.1482	True	0.940317790580855
41	Cc1cnc2c(O)cc3c([nH]c(=O)n3C)c2n1	0.1525	False	0.3854993128551255
45	Cc1cnc2c(O)cc3c(nc(NO)n3C)c2n1	0.1588	True	0.9671489112903668
46	Cn1c(N)nc2c3nccnc3ccc21	0.2840	True	0.9542529809532858
47	Cn1c(=O)[nH]c2c3nc(C(=O)O)cnc3ccc21	0.9197	False	0.1992342790924088
50	Cn1c(N)n(O)c2c3nc(C(=O)O)cnc3ccc21	0.2685	False	0.4093439372469093
51	Cn1c(NO)nc2c3nc(C(=O)O)cnc3ccc21	0.9144	True	0.9633731331898584
52	Cn1c(=O)[nH]c2c3nc(CO)cnc3ccc21	0.9019	False	0.8300962026492659
55	Cn1c(N)n(O)c2c3nc(CO)cnc3ccc21	0.1906	False	0.4799896955080949
56	Cn1c(NO)nc2c3nc(CO)cnc3ccc21	0.8954	True	0.9666158494872537
58	Cc1cn(O)c2ccc3c([nH]c(=O)n3C)c2n1	0.1566	False	0.0765082336780704
61	Cc1cn(O)c2ccc3c(nc(NO)n3C)c2n1	0.0650	False	0.7917549020978537
65	Cc1cnc2ccc3c(nc(NC4OC(C(=O)O)C(O)C(O)C4O)n3C)c2n1	0.6790	False	0.1446962821904339
71	Cc1cnc2ccc3[nH]c(NC4OC(C(=O)O)C(O)C(O)C4O)nc3c2n1	0.7690	False	0.14497195402967128
72	Cc1cnc2ccc3[nH]c(N)n(C4OC(C(=O)O)C(O)C(O)C4O)c3c2n1	0.1974	False	0.09406177705658296
73	Cc1cnc2ccc3[nH]c(N)nc3c2n1C1OC(C(=O)O)C(O)C(O)C1O	0.1316	False	0.09715657659495487

74	Cc1cn(C2OC(C(=O)O)C(O)C(O)C2O)c2ccc3[nH]c(N)nc3c2n1	0.2598	False	0.09715657680399216
75	Cc1cnc2ccc3c(nc(N)n3C3OC(C(=O)O)C(O)C(O)C3O)c2n1	0.3187	False	0.8204868913526789
76	Cc1cnc2ccc3[nH]c(NS(=O)(=O)O)nc3c2n1	0.5542	False	0.3086755846244513
77	CC(=O)Nc1nc2c(ccc3ncc(C)nc32)[nH]1	0.6859	False	0.3890355639166726
86	Cc1cnc2cc(OC3OC(C(=O)O)C(O)C(O)C3O)c3c(nc(N)n3C)c2n1	0.2250	False	0.8023074573739809
87	Cc1cnc2cc(O)c3c(nc(NC4OC(C(=O)O)C(O)C(O)C4O)n3C)c2n1	0.2023	False	0.12277015897840465
91	Cc1cnc2cc(OS(=O)(=O)O)c3c(nc(N)n3C)c2n1	0.3014	True	0.8951855339987089
92	Cc1cnc2cc(O)c3c(nc(NS(=O)(=O)O)n3C)c2n1	0.1290	False	0.2451431906838536
93	CC(=O)Nc1nc2c3nc(C)cnc3cc(O)c2n1C	0.2421	False	0.3375214971698557
99	Cc1cnc2c(OS(=O)(=O)O)cc3c(nc(N)n3C)c2n1	0.1037	True	0.9186650779421685
102	Cn1c(N)nc2c3nc(C(=O)OC4OC(C(=O)O)C(O)C(O)C4O)cnc3ccc21	0.7197	False	0.7946026634538524
103	Cn1c(NC2OC(C(=O)O)C(O)C(O)C2O)nc2c3nc(C(=O)O)cnc3ccc21	0.8353	False	0.10158491150115644
105	Cn1c(N)nc2c3nc(C(=O)O)cn(C4OC(C(=O)O)C(O)C(O)C4O)c3ccc21	0.1007	False	0.0554217411453125
106	Cn1c(N)n(C2OC(C(=O)O)C(O)C(O)C2O)c2c3nc(C(=O)O)cnc3ccc21	0.3244	False	0.0552739976181928
107	Cn1c(NS(=O)(=O)O)nc2c3nc(C(=O)O)cnc3ccc21	0.5631	False	0.14500233148252945
108	CC(=O)Nc1nc2c3nc(C(=O)O)cnc3ccc2n1C	0.7234	False	0.3020083644432914
109	Cn1c(N)nc2c3nc(COC4OC(C(=O)O)C(O)C(O)C4O)cnc3ccc21	0.6966	False	0.8319718029748999
110	Cn1c(NC2OC(C(=O)O)C(O)C(O)C2O)nc2c3nc(CO)cnc3ccc21	0.8127	False	0.6232642624468664
111	Cn1c(N)nc2c1ccc1ncc(CO)n(C3OC(C(=O)O)C(O)C(O)C3O)c12	0.1052	False	0.2974558001724153
112	Cn1c(N)nc2c3nc(CO)cn(C4OC(C(=O)O)C(O)C(O)C4O)c3ccc21	0.2576	False	0.2421130103601805
113	Cn1c(N)n(C2OC(C(=O)O)C(O)C(O)C2O)c2c3nc(CO)cnc3ccc21	0.2467	False	0.19825894633489416
114	Cn1c(NS(=O)(=O)O)nc2c3nc(CO)cnc3ccc21	0.5478	False	0.7633624995670347
115	Cn1c(N)nc2c3nc(COS(=O)(=O)O)cnc3ccc21	0.7437	True	0.9566845806231064
116	CC(=O)Nc1nc2c3nc(CO)cnc3ccc2n1C	0.7038	False	0.8001797422224037
117	Cc1cnc2ccc3c([nH]c(=O)n3C)c2n1C1OC(C(=O)O)C(O)C(O)C1O	0.1015	False	0.02553909311788967
118	Cc1cn(C2OC(C(=O)O)C(O)C(O)C2O)c2ccc3c([nH]c(=O)n3C)c2n1	0.2246	False	0.02158353560208915
119	Cc1cnc2ccc3c(c2n1)n(C1OC(C(=O)O)C(O)C(O)C1O)c(=O)n3C	0.1385	False	0.09763457340287646
135	Cc1cnc2ccc3c(nc(NOC4OC(C(=O)O)C(O)C(O)C4O)n3C)c2n1	0.2782	True	0.9299795372322708

136	Cc1cnc2ccc3c(nc(N(O)C4OC(C(=O)O)C(O)C(O)C4O)n3C)c2n1	0.1950	False	0.4486358508635255
137	Cc1cnc2ccc3c(c2n1)n(C1OC(C(=O)O)C(O)C(O)C1O)c(NO)n3C	0.1222	False	0.6092029312434818
139	Cc1cn(C2OC(C(=O)O)C(O)C(O)C2O)c2ccc3c(nc(NO)n3C)c2n1	0.1742	False	0.5345948478603479
143	Cc1cn(C2OC(C(=O)O)C(O)C(O)C2O)c2ccc3c(nc(NC4OC(C(=O)O)C(O)C(O)C4O)n3C)c2n1	0.1141	False	0.00757452767551932

PhIP

Metabolites_ID	SMILES_Formula	Production probability score	Reactive_to_DNA (>=0.85)	XenoSite Reactivity score
0	Cn1c(N)nc2ncc(-c3ccccc3)cc21	1.0000	True	0.9057459333311574
1	Nc1nc2ncc(-c3ccccc3)cc2[nH]1	0.8410	True	0.959387637670526
2	Cn1c(N)nc2ncc(-c3ccc(O)cc3)cc21	0.5050	True	0.9537529526142764
4	Cn1c(N)nc2ncc(-c3cccc(O)c3)cc21	0.5480	True	0.9537195442905044
6	Cn1c(=O)[nH]c2ncc(-c3ccccc3)cc21	0.6931	False	0.0642376242299052
9	Cn1c(NO)nc2ncc(-c3ccccc3)cc21	0.7880	True	0.9669464022397948
10	COc1cc(-c2cnc3nc(N)n(C)c3c2)ccc1O	0.5480	True	0.9480287919697864
12	Nc1nc2nc(O)c(-c3ccccc3)cc2[nH]1	0.4340	True	0.9544928470545476
15	O=c1[nH]c2cc(-c3ccccc3)cnc2[nH]1	0.7144	False	0.06579856824366599
18	ONc1nc2ncc(-c3ccccc3)cc2[nH]1	0.6741	True	0.971709627243492
20	Cn1c(N)nc2nc(O)c(-c3ccc(O)cc3)cc21	0.3010	True	0.9479677590433104
21	Cn1c(N)nc2ncc(-c3ccc(O)c(O)c3)cc21	0.3027	True	0.9479677589401496
23	Cn1c(=O)[nH]c2ncc(-c3ccc(O)cc3)cc21	0.4801	False	0.05810069832844205
25	Cn1c(N)n(O)c2ncc(-c3ccc(O)cc3)cc21	0.2464	False	0.5520313639664687
26	Cn1c(NO)nc2ncc(-c3ccc(O)cc3)cc21	0.4893	True	0.9713002167285292
27	Cn1c(N)nc2nc(O)c(-c3cccc(O)c3)cc21	0.3039	True	0.9479075231870252
29	Cn1c(=O)[nH]c2nc(O)c(-c3ccccc3)cc21	0.2264	False	0.07095711372210785
32	Cn1c(NO)nc2nc(O)c(-c3ccccc3)cc21	0.4035	True	0.9713002167592756
33	COc1cc(-c2cc3c(nc2O)nc(N)n3C)ccc1O	0.2537	True	0.9412249892655148
36	Cn1c(N)nc2ncc(-c3cccc(O)c3O)cc21	0.1137	True	0.9479075230839712

38	Cn1c(=O)[nH]c2ncc(-c3cccc(O)c3)cc21	0.5191	False	0.05752792224046956
40	Cn1c(N)n(O)c2ncc(-c3cccc(O)c3)cc21	0.3507	False	0.5530915186655545
41	Cn1c(NO)nc2ncc(-c3cccc(O)c3)cc21	0.5219	True	0.9713002167285292
42	Cn1c(=O)[nH]c2ncc(-c3ccccc3)c(O)c21	0.0826	False	0.07484085325393733
47	Cn1c(=O)[nH]c2c1cc(-c1ccccc1)cn2O	0.0886	False	0.1845209476878811
48	COc1cc(-c2cnc3[nH]c(=O)n(C)c3c2)ccc1O	0.4753	False	0.10366246742473892
52	Cn1c(NO)n(O)c2ncc(-c3ccccc3)cc21	0.1478	True	0.9262429526232714
53	COc1cc(-c2cnc3c(c2)n(C)c(N)n3O)ccc1O	0.2652	False	0.5007644859889226
54	COc1cc(-c2cnc3nc(NO)n(C)c3c2)ccc1O	0.4783	True	0.96897099789443
58	Cn1c(NC2OC(C(=O)O)C(O)C(O)C2O)nc2ncc(-c3ccccc3)cc21	0.7530	False	0.12145374210618748
60	Cn1c(N)n(C2OC(C(=O)O)C(O)C(O)C2O)c2ncc(-c3ccccc3)cc21	0.3640	False	0.09464373107451456
63	O=C(O)C1OC(Nc2nc3ncc(-c4ccccc4)cc3[nH]2)C(O)C(O)C1O	0.7300	False	0.12252888605716475
64	Nc1[nH]c2cc(-c3ccccc3)cnc2n1C1OC(C(=O)O)C(O)C(O)C1O	0.4037	False	0.0972211591231239
65	Nc1nc2c(cc(-c3ccccc3)cn2C2OC(C(=O)O)C(O)C(O)C2O)[nH]1	0.2220	False	0.09169036538536668
66	Nc1nc2ncc(-c3ccccc3)cc2n1C1OC(C(=O)O)C(O)C(O)C1O	0.2388	False	0.8412783598134883
67	O=S(=O)(O)Nc1nc2ncc(-c3ccccc3)cc2[nH]1	0.4878	False	0.08766060423839672
68	CC(=O)Nc1nc2ncc(-c3ccccc3)cc2[nH]1	0.6493	False	0.358261565747931
69	Cn1c(N)nc2ncc(-c3ccc(OC4OC(C(=O)O)C(O)C(O)C4O)cc3)cc21	0.5050	False	0.8492078425506253
70	Cn1c(NC2OC(C(=O)O)C(O)C(O)C2O)nc2ncc(-c3ccc(O)cc3)cc21	0.4423	False	0.10225086050154618
72	Cn1c(N)n(C2OC(C(=O)O)C(O)C(O)C2O)c2ncc(-c3ccc(O)cc3)cc21	0.3434	False	0.08401986383673721
73	Cn1c(N)nc2ncc(-c3ccc(OS(=O)(=O)O)cc3)cc21	0.4808	True	0.9283077736841796
74	Cn1c(NS(=O)(=O)O)nc2ncc(-c3ccc(O)cc3)cc21	0.2969	False	0.08032209556006872
75	CC(=O)Nc1nc2ncc(-c3ccc(O)cc3)cc2n1C	0.3818	False	0.3209659080057035
83	Cn1c(N)nc2ncc(-c3cccc(OC4OC(C(=O)O)C(O)C(O)C4O)c3)cc21	0.5436	False	0.8492078425506252
84	Cn1c(NC2OC(C(=O)O)C(O)C(O)C2O)nc2ncc(-c3cccc(O)c3)cc21	0.4954	False	0.10225086050154618
86	Cn1c(N)n(C2OC(C(=O)O)C(O)C(O)C2O)c2ncc(-c3cccc(O)c3)cc21	0.4099	False	0.07908372490666958
87	Cn1c(N)nc2ncc(-c3cccc(OS(=O)(=O)O)c3)cc21	0.5042	True	0.928371131062364
88	Cn1c(NS(=O)(=O)O)nc2ncc(-c3cccc(O)c3)cc21	0.3222	False	0.07681060739882067

89	CC(=O)Nc1nc2ncc(-c3cccc(O)c3)cc2n1C	0.4143	False	0.3089632602528161
98	Cn1c(=O)n(C2OC(C(=O)O)C(O)C(O)C2O)c2ncc(-c3ccccc3)cc21	0.3785	False	0.041588007492785815
107	Cn1c(NOC2OC(C(=O)O)C(O)C(O)C2O)nc2ncc(-c3ccccc3)cc21	0.3436	True	0.9398086493004248
108	Cn1c(N(O)C2OC(C(=O)O)C(O)C(O)C2O)nc2ncc(-c3ccccc3)cc21	0.2238	False	0.4115043471698325
109	Cn1c(NO)n(C2OC(C(=O)O)C(O)C(O)C2O)c2ncc(-c3ccccc3)cc21	0.3215	False	0.6370610518756213
111	COc1cc(-c2cnc3nc(N)n(C)c3c2)ccc1OC1OC(C(=O)O)C(O)C(O)C1O	0.5414	False	0.824970717275429
112	COc1cc(-c2cnc3nc(NC4OC(C(=O)O)C(O)C(O)C4O)n(C)c3c2)ccc1O	0.4799	False	0.08754793690023116
113	COc1cc(-c2cc3c(nc(N)n3C)n(C3OC(C(=O)O)C(O)C(O)C3O)c2)ccc1O	0.1184	False	0.062141228656356774
114	COc1cc(-c2cnc3c(c2)n(C)c(N)n3C2OC(C(=O)O)C(O)C(O)C2O)ccc1O	0.3726	False	0.06837664241127442
115	COc1cc(-c2cnc3nc(N)n(C)c3c2)ccc1OS(=O)(=O)O	0.5042	True	0.9179597233568728
116	COc1cc(-c2cnc3nc(NS(=O)(=O)O)n(C)c3c2)ccc1O	0.3222	False	0.08307232175487678
117	COc1cc(-c2cnc3nc(NC(C)=O)n(C)c3c2)ccc1O	0.4143	False	0.27830314455341865
119	Cn1c(NC2OC(C(=O)O)C(O)C(O)C2O)n(C2OC(C(=O)O)C(O)C(O)C2O)c2ncc(-c3ccccc3)cc21	0.1321	False	0.010254377675017844
120	Cn1c(NC2OC(C(=O)O)C(O)C(O)C2O)nc2c1cc(-c1ccccc1)cn2C1OC(C(=O)O)C(O)C(O)C1O	0.1265	False	0.007643960286787444
125	CC(=O)Nc1n(C)c2cc(-c3ccccc3)cnc2n1C1OC(C(=O)O)C(O)C(O)C1O	0.1966	False	0.05268674209226962
