# Supplementary material for: Constructing xenobiotic maps of metabolism to predict enzymes catalyzing metabolites capable of binding to DNA
Source: BMC Bioinformatics. 2021 Sep 21;22:450. doi: 10.1186/s12859-021-04363-6 (PMC8454073; doi:10.1186/s12859-021-04363-6)
Supplement: Supplementary file 7 — Additional file 7.: Distribution of 3 HAA production probability score The file provides the distribution of production probability scores used in Fig. 6 but in full page format. [file 12859_2021_4363_MOESM7_ESM.pdf]

Constructing xenobiotic maps of metabolism to predict enzymes catalyzing metabolites capable of binding to DNA.

Conan M., Théret N., Langouet S. and Siegel, A

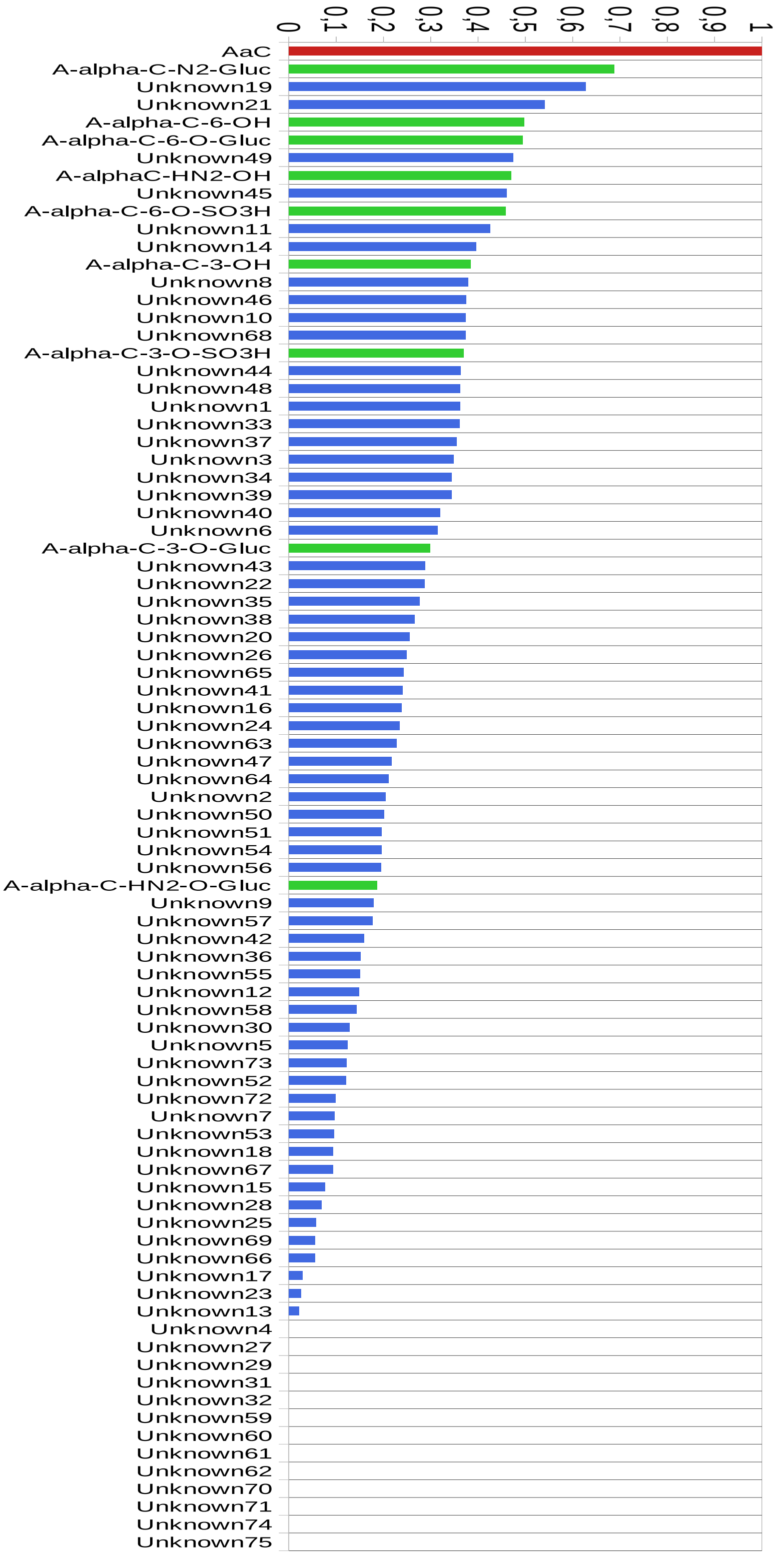

Enlarged version of Figure 6 (a) Distribution of prediction probability score of AαC. The original compound is shown with a red bar. Metabolites which have been experimentally observed are indicated by a green bar and other metabolites are indicated with a blue bar. X-axis :names of metabolites, they are either the name found for known metabolites or a two parts name : *Unknown* + *X* where X describes the identifier of the node in the map associated with this metabolite.

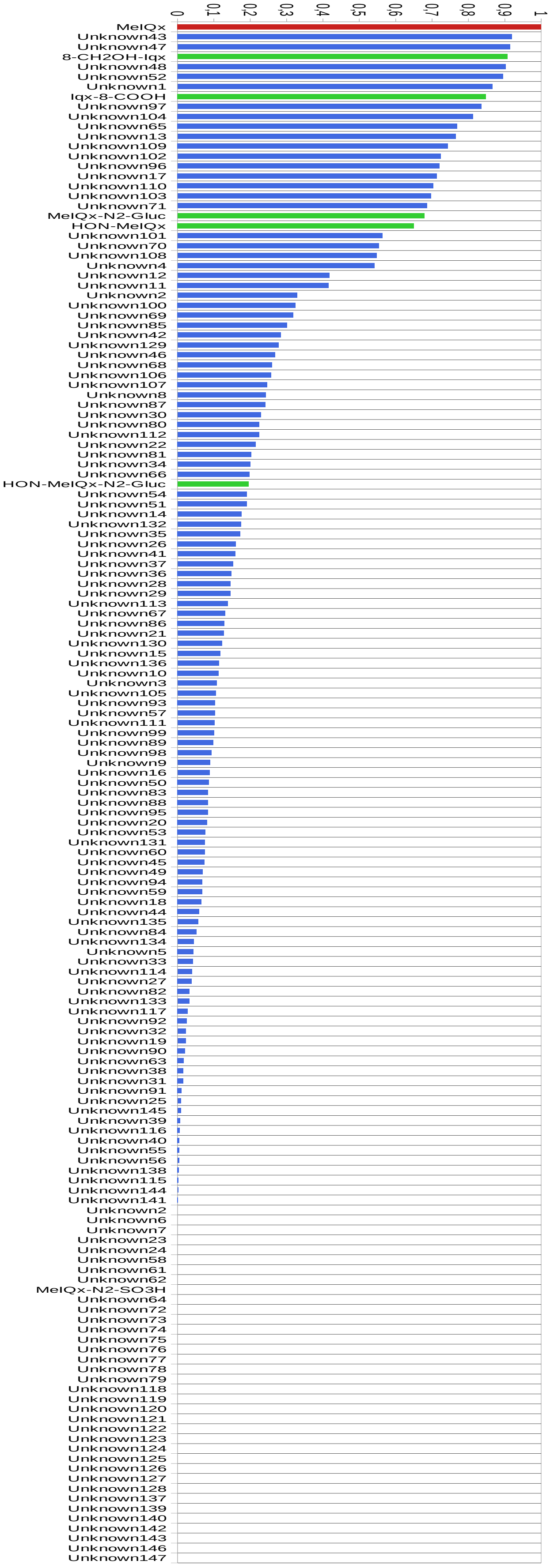

Enlarged version of Figure 6 (b) Distribution of prediction probability score of MeIQx. The original compound is shown with a red bar. Metabolites which have been experimentally observed are indicated by a green bar and other metabolites are indicated with a blue bar. X-axis :names of metabolites, they are either the name found for known metabolites or a two parts name : *Unknown* + *X* where X describes the identifier of the node in the map associated with this metabolite.

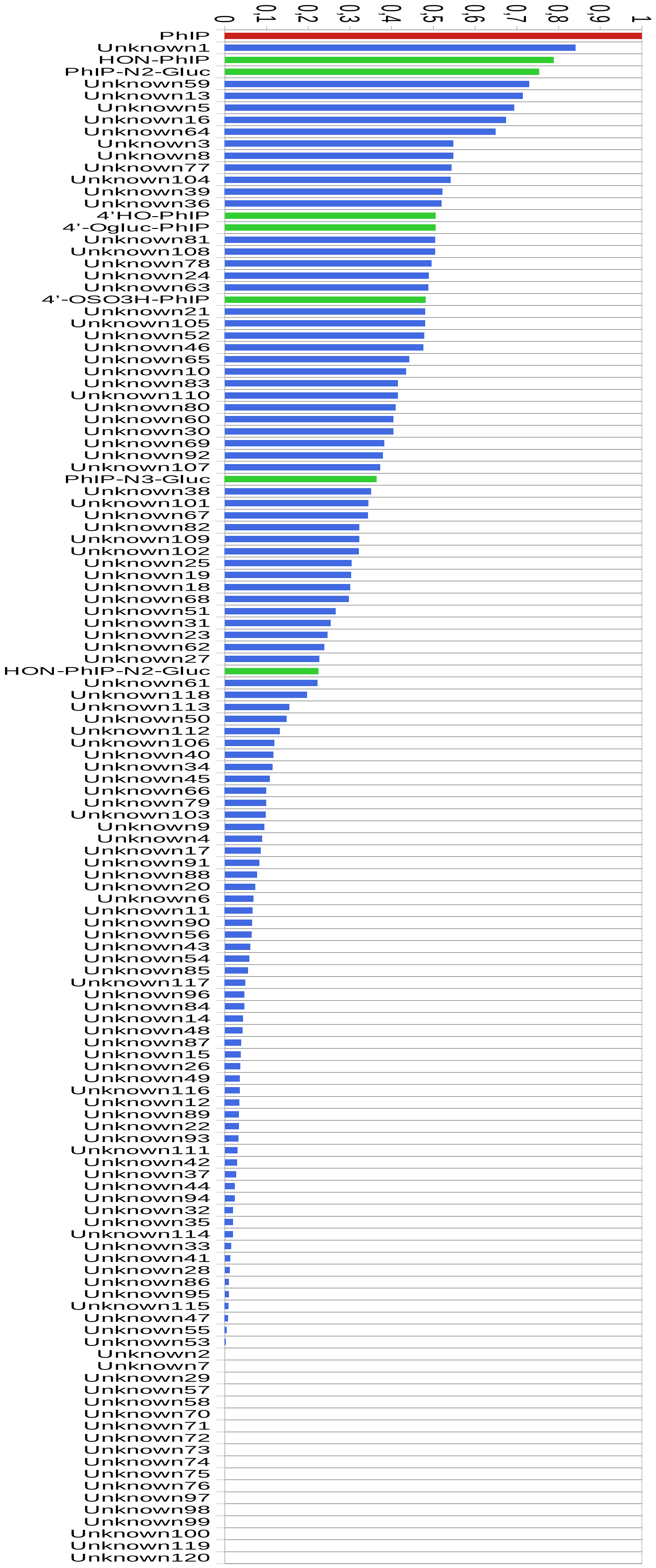

Enlarged version of Figure 6 (c) Distribution of prediction probability score of PhIP. The original compound is shown with a red bar. Metabolites which have been experimentally observed are indicated by a green bar and other metabolites are indicated with a blue bar. X-axis :names of metabolites, they are either the name found for known metabolites or a two parts name : *Unknown* + *X* where X describes the identifier of the node in the map associated with this metabolite.
