# Supplementary material for: Constructing xenobiotic maps of metabolism to predict enzymes catalyzing metabolites capable of binding to DNA
Source: BMC Bioinformatics. 2021 Sep 21;22:450. doi: 10.1186/s12859-021-04363-6 (PMC8454073; doi:10.1186/s12859-021-04363-6)
Supplement: Supplementary file 8 — Additional file 8.: Description of six HAA maps of metabolism The file provides a detailed description of metabolites for the six HAA filtered maps of metabolism built in the paper. For each metabolite of each map, the file provides the identifier of the metabolite, its SMILES formula, its production probability score, its reactivity to DNA and the score of XenoSite Reactivity. [file 12859_2021_4363_MOESM8_ESM.pdf]

Constructing xenobiotic maps of metabolism to predict enzymes catalyzing metabolites capable of binding to DNA

Conan M., Théret N., Langouet S. and Siegel, A

Metabolite	Found in predicted metabolism map
AαC	
AαC-3-O-Gluc	
AαC-3-OH	
AαC-3-O-SO3H	
AαC-6-O-Gluc	
AαC-6-OH	
AαC-6-O-SO3H	
AαC-HN^2-O-Gluc *	
AαC-HN^2-OH *	
AαC-N^2-Gluc	
N-Acetoxy-AαC *	
N-Sulfonyloxy-AαC *	
MeIQx	
7-oxo-MeIQx	
8-CH_2OH-Iqx	
HON-MeIQx *	
HON-MeIQx-N^2-Gluc	
Iqx-8-COOH	
MeIQx-N^2-Gluc	
MeIQx-N^2-SO3H	
N-Acetoxy-MeIQx	
N-desmethyl-7-oxo-MeIQx *	
N-Sulfonyloxy-MeIQx *	
PhIP	
4'HO-PhIP	
4'-Ogluc-PhIP	
4'-OSO3H-PhIP	
HON-PhIP *	
HON-PhIP-N^2-Gluc	
PhIP-N^2-Gluc	
PhIP-N^3-Gluc	
N-Acetoxy-PhIP *	
N-Sulfonyloxy-PhIP *	

Experimentally identified metabolites of AαC, PhIP and MeIQx founded in metabolism map predicted by SyGMa. Metabolites with an asterisk are metabolites identified as precursor of DNA adducts and comming from Delannée et al. 2019
