# Supplementary material for: Constructing xenobiotic maps of metabolism to predict enzymes catalyzing metabolites capable of binding to DNA
Source: BMC Bioinformatics. 2021 Sep 21;22:450. doi: 10.1186/s12859-021-04363-6 (PMC8454073; doi:10.1186/s12859-021-04363-6)
Supplement: Supplementary file 12 — Additional file 12.: Metabolism map of 4,7,8-TriMeIQx A representation of the filtered metabolism map of 4,7,8-TriMeIQx with chemical structures. [file 12859_2021_4363_MOESM12_ESM.pdf]

Constructing xenobiotic maps of metabolism to predict enzymes catalyzing metabolites capable of binding to DNA.

Conan M., Théret N., Langouet S. and Siegel, A

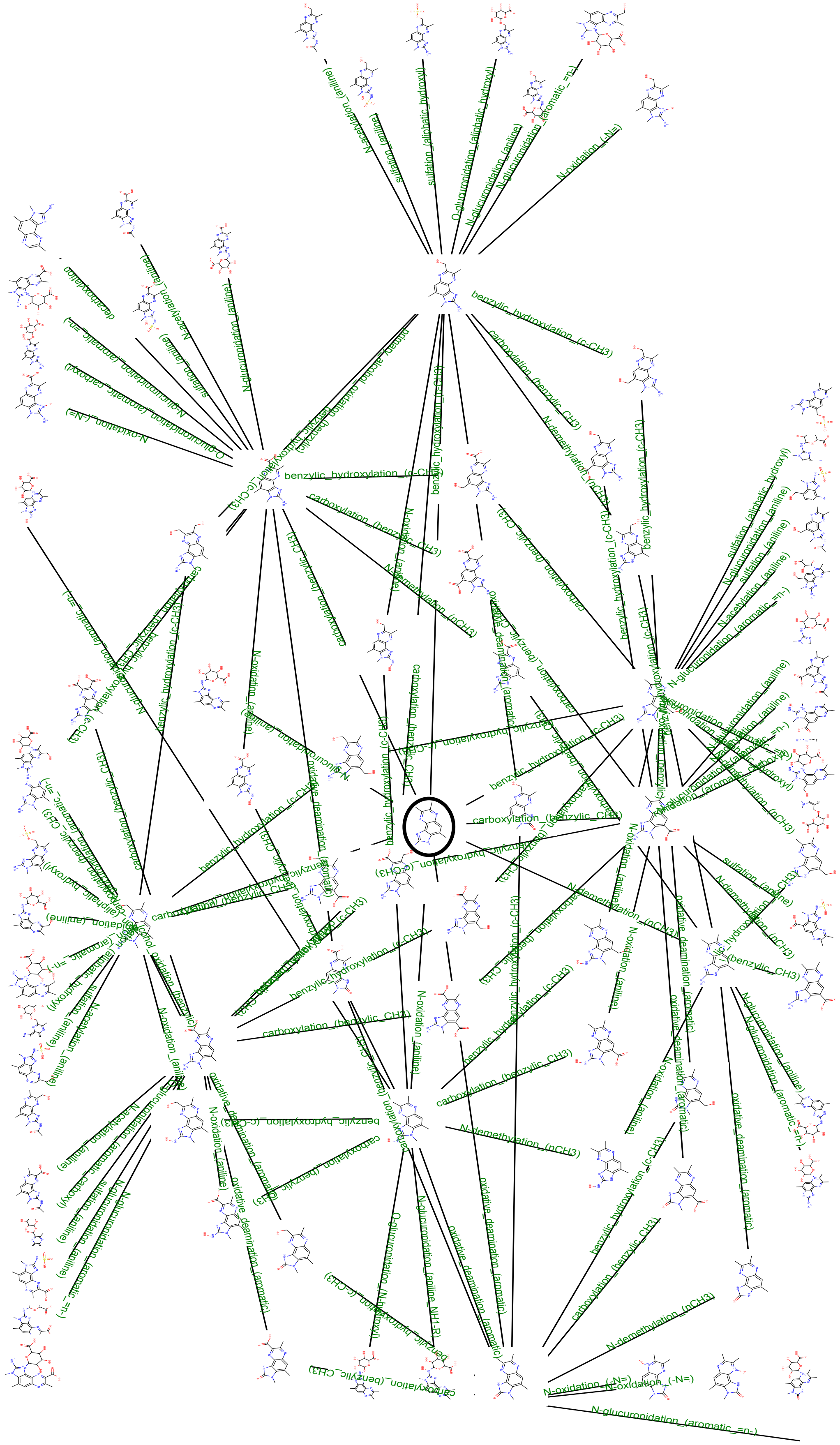

Additional file 12 Filtered map of metabolism of 4,7,8-TriMeIQx. Metabolites are represented by their 2D structures and text on edges is the SMIRKS rule label leading to the production of associated metabolite. The circled metabolite is the original compound.
