# Supplementary figures and images for: Constructing xenobiotic maps of metabolism to predict enzymes catalyzing metabolites capable of binding to DNA
Source: BMC Bioinformatics. 2021 Sep 21;22:450. doi: 10.1186/s12859-021-04363-6 (PMC8454073; doi:10.1186/s12859-021-04363-6)

Conan M., Th  ret N., Langouet S. and Siegel, A

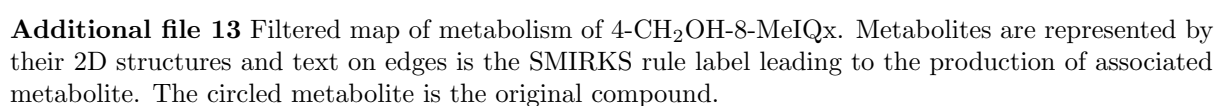

Supplement: Supplementary file 13 — Additional file 13.: Metabolism map of 4-CH2OH-8-MeIQx A representation of the filtered metabolism map of 4-CH2OH-8-MeIQx with chemical structures. [file 12859_2021_4363_MOESM13_ESM.pdf]
