# Supplementary material for: Constructing xenobiotic maps of metabolism to predict enzymes catalyzing metabolites capable of binding to DNA
Source: BMC Bioinformatics. 2021 Sep 21;22:450. doi: 10.1186/s12859-021-04363-6 (PMC8454073; doi:10.1186/s12859-021-04363-6)
Supplement: Supplementary file 11 — Additional file 11.: Metabolism map of PhIP A representation of the filtered metabolism map of PhIP with chemical structures. [file 12859_2021_4363_MOESM11_ESM.pdf]

Constructing xenobiotic maps of metabolism to predict enzymes catalyzing metabolites capable of binding to DNA.

Conan M., Théret N., Langouet S. and Siegel, A

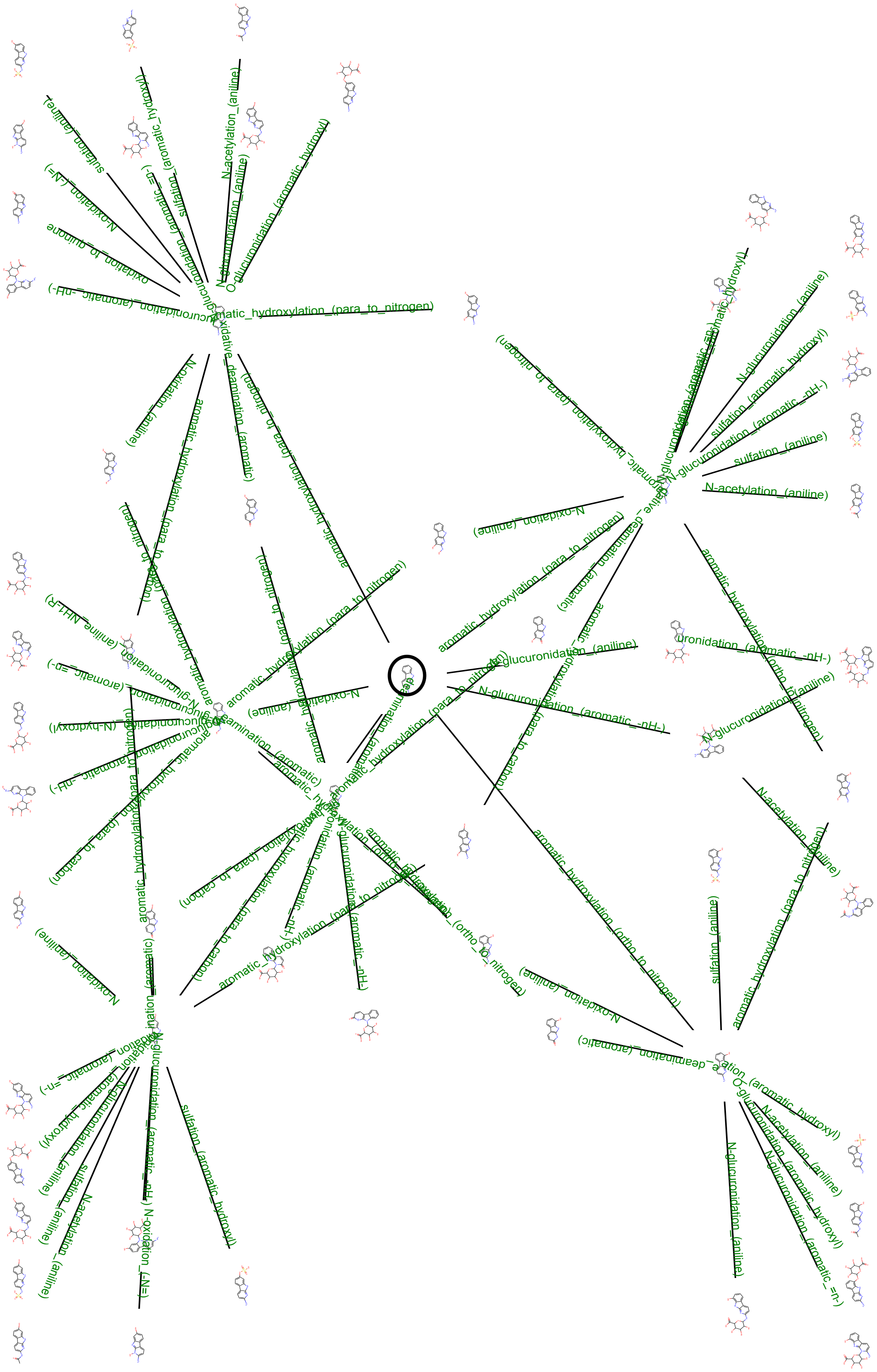

Additional file 11 Filtered map of metabolism of PhIP. Metabolites are represented by their 2D structures and text on edges is the SMIRKS rule label leading to the production of associated metabolite. The circled metabolite is the original compound.
